# SPATA2 Links CYLD to LUBAC, Activates CYLD, and Controls LUBAC Signaling

**DOI:** 10.1016/j.molcel.2016.08.001

**Published:** 2016-09-15

**Authors:** Paul R. Elliott, Derek Leske, Matous Hrdinka, Katrin Bagola, Berthe K. Fiil, Stephen H. McLaughlin, Jane Wagstaff, Norbert Volkmar, John C. Christianson, Benedikt M. Kessler, Stefan M.V. Freund, David Komander, Mads Gyrd-Hansen

**Affiliations:** 1 Division of Protein and Nucleic Acid Chemistry, MRC Laboratory of Molecular Biology, Francis Crick Avenue, Cambridge CB2 0QH, UK; 2 Ludwig Institute for Cancer Research, Nuffield Department of Medicine, University of Oxford, Old Road Campus Research Building, Oxford OX3 7DQ, UK; 3 TDI Mass Spectrometry Laboratory, Target Discovery Institute, Nuffield Department of Medicine, University of Oxford, Roosevelt Drive, Oxford OX3 7FZ, UK

## Abstract

The linear ubiquitin chain assembly complex (LUBAC) regulates immune signaling, and its function is regulated by the deubiquitinases OTULIN and CYLD, which associate with the catalytic subunit HOIP. However, the mechanism through which CYLD interacts with HOIP is unclear. We here show that CYLD interacts with HOIP via spermatogenesis-associated protein 2 (SPATA2). SPATA2 interacts with CYLD through its non-canonical PUB domain, which binds the catalytic CYLD USP domain in a CYLD B-box-dependent manner. Significantly, SPATA2 binding activates CYLD-mediated hydrolysis of ubiquitin chains. SPATA2 also harbors a conserved PUB-interacting motif that selectively docks into the HOIP PUB domain. In cells, SPATA2 is recruited to the TNF receptor 1 signaling complex and is required for CYLD recruitment. Loss of SPATA2 increases ubiquitination of LUBAC substrates and results in enhanced NOD2 signaling. Our data reveal SPATA2 as a high-affinity binding partner of CYLD and HOIP, and a regulatory component of LUBAC-mediated NF-κB signaling.

## Highlights

• CYLD recruitment to LUBAC and the TNF receptor 1 complex is mediated by SPATA2
• SPATA2 forms a high-affinity complex with CYLD and stimulates CYLD’s activity
• SPATA2, like OTULIN, uses a conserved PIM to dock to the HOIP PUB domain
• SPATA2 limits ubiquitination of LUBAC substrates to regulate inflammatory signaling

## Graphical Abstract

**Figure:**
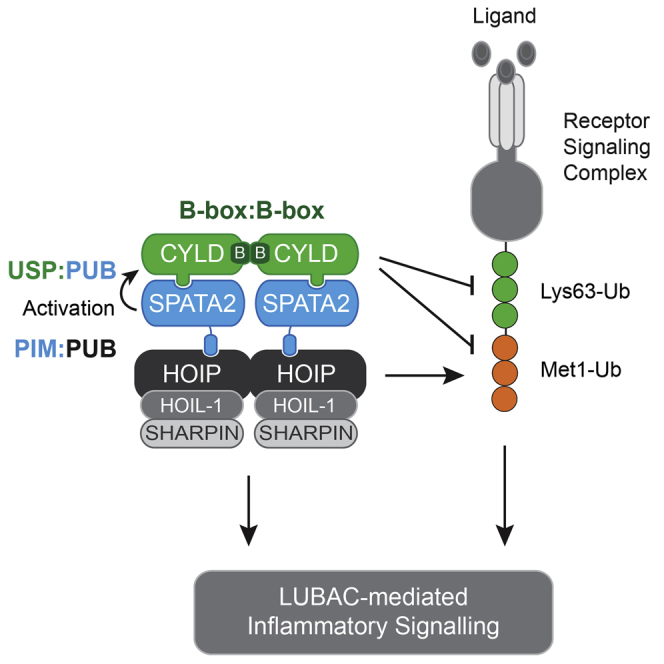

## Teaser

Elliott et al. show that SPATA2 bridges CYLD with LUBAC to regulate substrate ubiquitination and inflammatory signaling. Structural and biochemical work defines SPATA2-CYLD and SPATA2-HOIP interfaces, reveals SPATA2-mediated CYLD activation, and provides first insights into stoichiometry of LUBAC complexes.

## Introduction

Modification of proteins with ubiquitin (Ub) constitutes a versatile posttranslational modification that regulates a variety of cellular processes, including receptor signaling, cell cycle progression, and DNA damage responses. Ub signaling controls activation of nuclear factor-κB (NF-κB) and innate immune responses downstream of pattern recognition receptors (PRRs) such as Toll-like receptors (TLRs), nucleotide-oligomerization domain (NOD)-like receptors, and cytokine receptors, such as tumor necrosis factor (TNF) receptor 1 (TNFR1) (Fiil and Gyrd-Hansen, 2014, Jiang and Chen, 2011).

Stimulation of these receptors triggers assembly of multi-protein signaling complexes where Ub ligases and deubiquitinases (DUBs) coordinate the deposition of Ub chains linked via lysine 63 (Lys63-Ub) and methionine 1 (Met1-Ub) on protein substrates to orchestrate activation of the TAB-TAK1 and NEMO-IKKα/β kinase complexes, respectively. Activation of IKK is required for productive signaling and NF-κB-mediated transcriptional responses, and its activation depends on the binding of Met1-Ub by the IKK subunit NEMO (also known as IKKγ) (Fiil and Gyrd-Hansen, 2014, Jiang and Chen, 2011).

Met1-Ub is conjugated by the linear ubiquitin chain assembly complex (LUBAC), composed of HOIP, HOIL-1, and SHARPIN, which has emerged as an important Ub ligase activity in innate immune signaling and immune regulation (Boisson et al., 2012, Boisson et al., 2015, Damgaard et al., 2012, Gerlach et al., 2011, Ikeda et al., 2011, Kirisako et al., 2006, Tokunaga et al., 2011). In cells, LUBAC function is regulated by at least two associated DUBs, OTULIN and CYLD, which serve both overlapping and unique roles. OTULIN exclusively hydrolyzes Met1-Ub, prevents spurious accumulation of Met1-Ub on LUBAC components under basal conditions, and restricts ubiquitination of LUBAC substrates such as RIPK2 after NOD2 stimulation (Fiil et al., 2013, Keusekotten et al., 2013). CYLD, a bona fide tumor suppressor and negative regulator of NF-κB signaling (Harhaj and Dixit, 2012), disassembles both Met1-Ub and Lys63-Ub (Komander et al., 2009, Ritorto et al., 2014, Sato et al., 2015). CYLD is recruited with LUBAC to TNFR1 and NOD2 signaling complexes and trims Ub chains on LUBAC substrates (Draber et al., 2015, Hrdinka et al., 2016, Takiuchi et al., 2014).

Both CYLD and OTULIN associate with LUBAC via an N-terminal peptide:N-glycanase/UBA- or UBX-containing protein (PUB) domain in the catalytic subunit HOIP (Draber et al., 2015, Elliott et al., 2014, Hrdinka et al., 2016, Schaeffer et al., 2014, Takiuchi et al., 2014). OTULIN harbors a PUB-interacting motif (PIM) that inserts into a PIM binding pocket in the HOIP PUB domain to create a high-affinity interaction important for its ability to counteract LUBAC auto-ubiquitination (Elliott et al., 2014, Schaeffer et al., 2014). The association of CYLD with LUBAC and its recruitment to receptor complexes also involves the PIM binding pocket in the HOIP PUB domain (Draber et al., 2015, Hrdinka et al., 2016, Takiuchi et al., 2014), but the molecular basis for the interaction is not understood.

Here, we show that CYLD does not interact directly with HOIP and identify the uncharacterized protein spermatogenesis-associated protein 2 (SPATA2) as the factor that bridges CYLD and HOIP. SPATA2 contains a PIM that binds the PUB domain in HOIP, but not other PUB domains. SPATA2 binds the USP domain of CYLD via its PUB domain, but in a PIM-independent manner. Interestingly, this interaction also activates CYLD. Functionally, SPATA2 mediates the recruitment of CYLD to the TNFR1 signaling complex and supports CYLD-dependent regulation of LUBAC-mediated NF-κB signaling.

## Results

### SPATA2 Binds CYLD in a B-box-Dependent Manner

**Figure 1:**
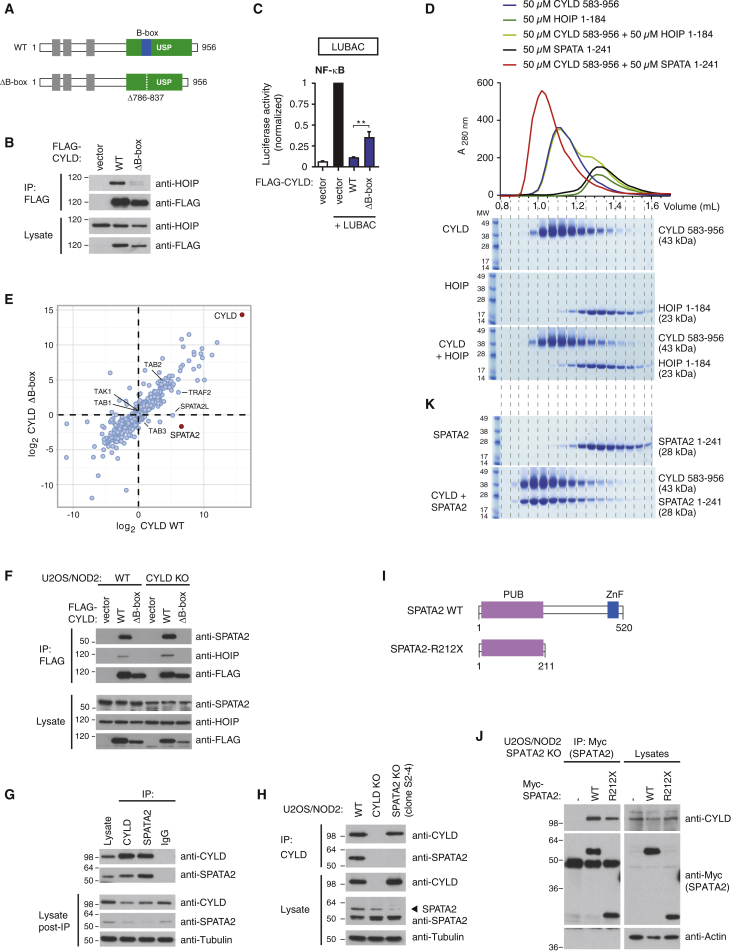
Identification of SPATA2 as a B-box-Dependent CYLD Interactor (A) Schematic representation of full-length and ΔB-box (deletion of aa residues 786–837) CYLD. (B) Immunoprecipitation and western blot analysis of transiently expressed FLAG-CYLD variants from CYLD KO U2OS/NOD2 cells. (C) NF-κB activity in U2OS/NOD2 cell lysates transfected with dual luciferase reporters and co-expressed with vector, LUBAC (HOIL-1/HOIP), and/or FLAG-CYLD variants as indicated. Luciferase activity is shown relative to the activity of LUBAC in transfected cells. Data represent the mean ± SEM of six independent experiments, each performed in duplicate. ^∗∗^p < 0.01. (D) Analytical SEC profile of CYLD USP (583–956) (blue), HOIP PUB domain (1–184) (green), and HOIP and CYLD at equimolar ratio (light green). Coomassie-stained SDS-PAGE gels below show protein-containing fractions. Also included are SPATA2 PUB domain (1–241) (black) and CYLD and SPATA2 at equimolar ratio (red) (see K). (E) Mass spectrometry analysis of FLAG-CYLD interactomes purified with anti-FLAG from CYLD KO U2OS cell lysates. The scatterplot shows enrichment of proteins co-purified with CYLD WT (x axis) and CYLD ΔB-box (y axis). Dots below the diagonal indicate B-box-dependent interactors. (F) Immunoprecipitation and western blot analysis of transiently expressed FLAG-CYLD variants from WT or CYLD KO U2OS/NOD2 cells. (G) Immunoprecipitation and western blot analysis of endogenous CYLD and SPATA2 from U2OS/NOD2 cells. Control IgG served as negative control. (H) Immunoprecipitation and western blot analysis of transiently expressed FLAG-CYLD from WT, CYLD KO, or SPATA2 KO (clone S2-4) U2OS/NOD2 cells. (I) Schematic representation of SPATA2 WT and SPATA2 R212X (SPATA2 aa 1–211). (J) Immunoprecipitation and western blot analysis of transiently expressed Myc-SPATA2 variants from SPATA2 KO U2OS/NOD2 cells. (K) Coomassie-stained SDS-PAGE gels (linked to profile in D) of SPATA2 PUB and CYLD USP-SPATA2 PUB complex. Also see Figure S1.

In cells, CYLD interaction with HOIP depends on the PIM-binding pocket within the HOIP PUB domain (Draber et al., 2015, Hrdinka et al., 2016, Takiuchi et al., 2014). Mutational analysis of CYLD showed that the interaction is mediated by the CYLD USP domain and depends on the CYLD B-box (Takiuchi et al., 2014) (Figures 1A and 1B). Deletion of the CYLD B-box impaired the ability of CYLD to antagonize LUBAC-mediated NF-κB activity, suggesting that this region in CYLD regulates LUBAC function (Figures 1C and S1A, available online). However, CYLD does not contain a discernible PIM within this region and there was no obvious binding between the CYLD USP and HOIP PUB domain, as determined by size-exclusion chromatography (SEC), where CYLD and HOIP eluted in separate fractions (Figure 1D), in vitro pull-downs (Figure S1B), or nuclear magnetic resonance (NMR) spectroscopy (data not shown) using purified proteins. This prompted us to search for a protein that would mediate the interaction between CYLD and HOIP. For this, we purified FLAG-tagged wild-type (WT) CYLD and CYLD with deletion of the B-box (ΔB-box) from CYLD knockout (KO) U2OS/NOD2 cells (Figure 1B) and subjected the purified material to liquid chromatography-tandem mass spectrometry (LC-MS/MS). Among the detected proteins were previously described CYLD interactors such as TAK1 (Reiley et al., 2007), TNF receptor-associated factor 2 (TRAF2) (Kovalenko et al., 2003), and also the known CYLD interactors SPATA2 and SPATA2-like (SPATA2L) (Sowa et al., 2009). Strikingly, SPATA2 was the most highly enriched protein in the CYLD WT sample relative to the CYLD ΔB-box sample, indicating that the interaction depends on the CYLD B-box (Figure 1E; Table S1). Also, SPATA2L was preferentially enriched by CYLD WT whereas TAK1-TAB components and TRAF2 were co-purified similarly with CYLD WT and CYLD ΔB-box (Figure 1E; Table S1). The interaction between SPATA2 and CYLD was confirmed in cells by co-immunoprecipitation of ectopic or endogenous proteins (Figures 1F and 1G). To ensure that SPATA2 was detected by the SPATA2 antibody in the CYLD immunoprecipitation, the specificity of the antibody was carefully characterized in cells where SPATA2 was depleted by RNAi-mediated silencing and in cells where SPATA2 had been genetically deleted by CRISPR/Cas9 genome editing (Figures 1H and S1C–S1E). This confirmed that the antibody detected SPATA2 in cell lysates and in CYLD immunoprecipitation experiments, but it also showed that the antibody detected several unrelated proteins in lysates, some of which migrated at a similar molecular weight (MW) as SPATA2 (Figures 1H and S1C–S1F). We then analyzed which region of SPATA2 was responsible for CYLD binding. This showed that the SPATA2 N-terminal PUB domain mediates CYLD interaction in cells (Figures 1I and 1J). Indeed, the CYLD USP domain (aa 583–956) and SPATA2 PUB domain (aa 1–241) formed an SEC-stable complex (Figure 1K), confirming a direct interaction.

### Characterization of the CYLD-SPATA2 Interaction

**Figure 2:**
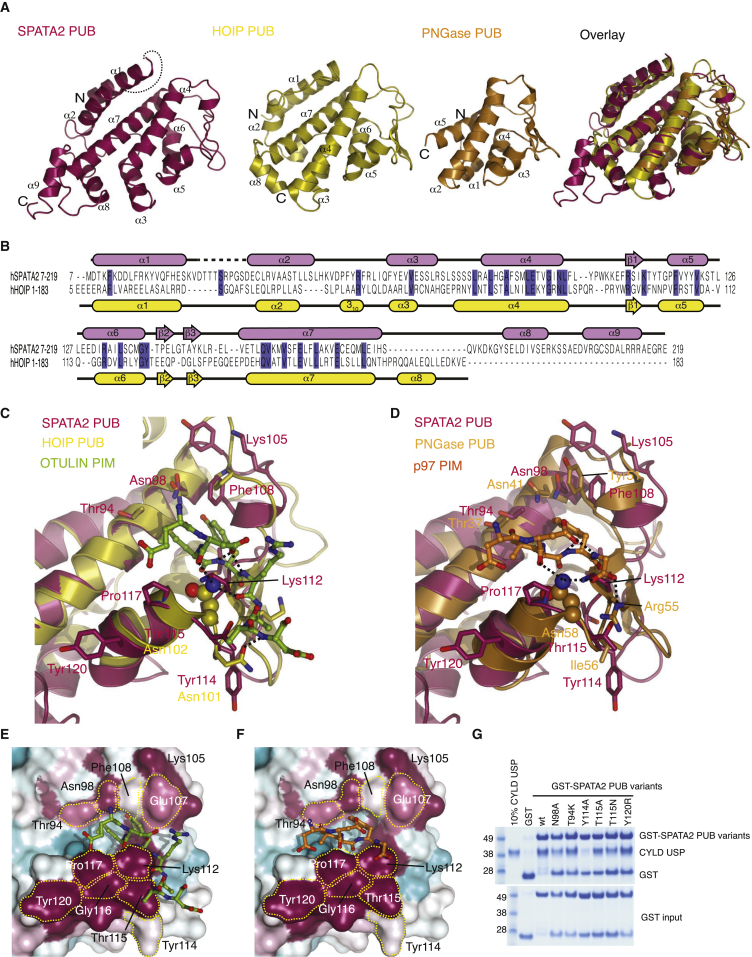
Structure of the SPATA2 PUB Domain (A) Far left, structure of the SPATA2 PUB domain (residues 7–219). Left, structure of the HOIP PUB domain (residues 1–184, PDB: 40YJ). Right, structure of the PNGase PUB domain (residues 12–110, PDB: 2HPJ). Far right, superimposition of all PUB domains on the α4, α7 core helices. (B) Structure-based sequence alignment of SPATA2 and HOIP PUB domains. (C) Superposition of SPATA2 onto HOIP PUB bound to OTULIN PIM (PDB: 40YK, PIM in green). Asn102, important for OTULIN PIM binding, is shown in a ball-and-stick model. (D) Superposition of SPATA2 and PNGase PUB bound to p97 PIM (PDB: 2HPL). (E and F) Conservation analysis of the SPATA2 PIM pocket (purple, conserved; white/cyan, not conserved). PIM peptides from OTULIN (E) and p97 (F) are modeled, revealing significant clashes. (G) Pull-down using GST-SPATA2 PUB domain and mutants within the PIM pocket against the CYLD USP domain. Also see Figure S2.

It was striking that while CYLD was unable to form a stable complex with the PUB domain of HOIP, it instead interacted with the PUB domain in SPATA2 (Figures 1D, 1K, and S1B). A crystal structure of the SPATA2 PUB domain (aa 7–219) at 1.45 Å resolution (Figures 2A–2D, S2A, and S2B; Table 1) revealed a fold most similar to that of the extended PUB domain in HOIP (root-mean-square deviation [RMSD] 2.4 Å) (Elliott et al., 2014) (Figures 2A–2C) and the smaller PUB domain of PNGase (RMSD 3.2 Å) (Zhao et al., 2007) (Figure 2D). Species conservation of SPATA2 mapped onto the surface of the PUB domain reveals that while the PIM pocket is highly conserved (Figures 2E, 2F, S2A, and S2C), this interaction site is also very different from canonical PUB domains. The previously mapped PUB-PIM interactions include a conserved Asp-Leu/Met-Tyr (see below), in which Leu and Tyr occupy a deep, hydrophobic gorge on the PUB surface, the PIM pocket. In SPATA2, this pocket is significantly different from both HOIP as well as PNGase structures, and modeling of interactions with PIM peptides derived from OTULIN or p97 would generate steric clashes (Figures 2E and 2F). Consistently, the SPATA2 PUB domain does not bind PIM peptides (see below), suggesting an interaction motif in CYLD may need to display distinct properties. Nonetheless, the high conservation in this area did suggest that this surface may mediate CYLD interactions, and single amino acid mutations in or near the SPATA2 PIM pocket interfered with CYLD binding (Figure 2G). In particular, mutations in the “lower wall” of the SPATA2 PIM pocket (Y114A, T115N, and T115A) decreased CYLD interactions, while mutation of residues in the “upper wall” of the pocket (N98A and T94K) did not have strong effects on CYLD binding. The strongest effect on CYLD binding was observed when we mutated Tyr114, which points away from the PIM pocket (Figures 2E and 2G), supporting that CYLD binds the SPATA2 PUB domain in a PIM-independent manner.

### The CYLD B-box Mediates CYLD Dimerization and Is Essential for SPATA2 Complex Formation

The B-box dependence of the CYLD-SPATA2 interaction (Figures 1B, 1C, and 1F) could suggest a direct interaction between the B-box and the SPATA2 PUB domain. Surprisingly, NMR titration experiments with an isolated ^15^N-labeled B-box domain (aa 778–855) and unlabeled SPATA2 PUB domain revealed no signs of an interaction (Figure S3A). This contrasts the formation of a stable complex on gel filtration between the SPATA PUB domain and the CYLD USP domain (Figure 1K).

**Figure 3:**
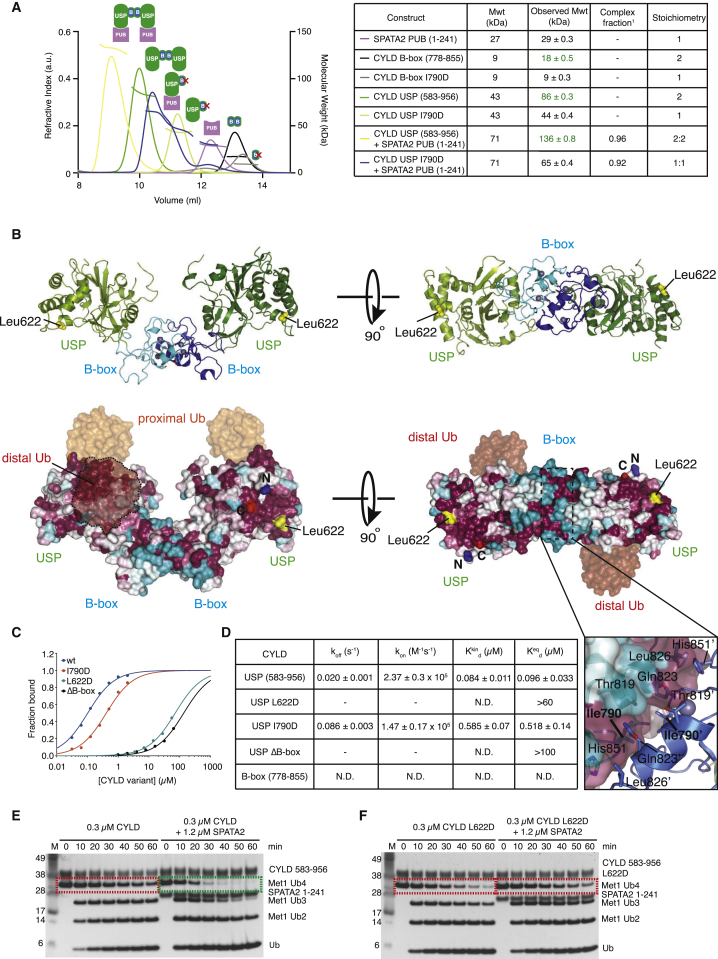
Mapping the SPATA2 Interaction Site on CYLD (A) SEC-MALS analysis of CYLD and SPATA2 PUB variants using a Superdex 75 size-exclusion column. Right, table listing monomeric and observed MWs for each protein or complex, fraction of complex formed (calculated from difference between expected and observed MW; see Supplemental Experimental Procedures), and the resultant stoichiometry. (B) Composite structure of human CYLD containing the B-box (PDB: 2VHF) bound to Met1-linked diUb (PDB: 3WXE). The crystal structure of human CYLD contains a crystallographic-related interface between the B-box domain, forming a 2-fold symmetrical axis. Top, cartoon representation of the CYLD dimer with the B-box colored blue and the USP domain in green. Met1-diUb is not shown for clarity. Bottom, identical view as above, but CYLD is shown as a surface colored according to sequence conservation. Met1-diUb is shown as a semi-transparent surface. Leu622 as well as N and C termini are highlighted. Insert, B-box dimerization interface where the surface of one B-box has been removed to highlight the conserved hydrophobic and polar contacts that form across the dimer interface. (C) Equilibrium response fitting for CYLD variants based on surface plasmon resonance (SPR) data. The fraction of CYLD variant bound to SPATA2 PUB is plotted against varying CYLD concentrations. (D) Table summarizing SPR data. The *k*_*off*_, *k*_*on*_ and the kinetic dissociation constant, *K*^*kin*^_*d*_ ( = *k*_*off*_*/k*_*on*_), are calculated from the response curves in Figure S3E. The equilibrium dissociation constant *K*^*eq*^_*d*_ is calculated from (C). No binding was observed from the CYLD B-box and only weak binding was observed from CYLD L622D and CYLD ΔB-box, preventing accurate determination of *K*^*eq*^_*d*_. For comparison, CYLD L622D and CYLD ΔB-box data were fitted assuming the maximum responses were similar to WT. N.D., not detected. (E) SPATA2 binding to CYLD enhances Ub chain hydrolysis. Hydrolysis of Met1-linked tetraUb by CYLD if followed over time without or with addition of SPATA2. Green boxes highlight the tetraUb band. (F) DUB assay as in (E) using CYLD L622D mutant, which is unable to bind SPATA2 and does not show enhanced activity. Also see Figure S3.

Further studies using SEC coupled to multi-angle light scattering (SEC-MALS) revealed that the intact CYLD USP domain (aa 583–956, including the B-box, 43 kDa) eluted as a dimer (86 kDa), while CYLD ΔB-box (35 kDa) eluted as a monomer (34 kDa) (Figures 3A and S3B). Furthermore, the isolated B-box domain (aa 778–855, 9.1 kDa) eluted as a dimer (17.1 kDa) (Figure 3A). Strikingly, the SPATA2 PUB domain (aa 1–241, 27.6 kDa), a monomer on its own (26.6 kDa), formed a 2:2 complex with dimeric CYLD USP domain of 136 kDa (calculated 140 kDa) in SEC-MALS (Figure 3A). This was independently confirmed by equilibrium analytical ultracentrifugation (Figure S3C). Thus, nicely consistent with the earlier results from mass spectrometry (Figure 1E), this complex forms in a B-box-dependent manner (Figures 3A and S3B).

Previous structural analysis of the CYLD USP domain (Komander et al., 2008) suggested how the B-box domain might mediate CYLD dimerization. A conserved B-box surface forms a hydrophobic interface across a crystallographic symmetry axis, which orients the two catalytic domains such that both can access polyUb without steric hindrance (Komander et al., 2008, Sato et al., 2015) (Figure 3B). Mutation of Ile790 (I790D) within the B-box dimerization interface generated a monomeric B-box (9 kDa) and monomeric CYLD USP domain (44 kDa) (Figure 3A). Interestingly, the I790D dimerization mutant still formed a 1:1 complex with the SPATA2 PUB domain of 65 kDa that was less stable on SEC-MALS (Figure 3A, blue profile). These results were corroborated by in vitro pull-downs and surface plasmon resonance (SPR), which revealed the CYLD-SPATA2 interaction to be high affinity (96 nM), and also showed no binding of the isolated B-box domain to SPATA2 (Figures 3C, 3D, S3D, and S3E). CYLD I790D affinity for SPATA2 was still respectable (518 nM), but a higher *k*_*off*_ likely affects stability of the complex when CYLD is not dimeric (Figures 3C, 3D, and S3E).

Surface conservation depicted on the CYLD dimer revealed that while exposed areas of the B-box were not conserved, a highly conserved surface exists on the solvent-exposed side of the CYLD palm domain not involved in Ub interactions (Figure 3B). Mutation of conserved surface residues revealed that Leu622 was essential for SPATA2 interaction (Figure S3D). Leu622 is 45 Å away from the B-box domain, indicating that the catalytic USP core of CYLD mediates SPATA2 binding. Hence, CYLD and SPATA2 form a highly stable heterotetramer in vitro and likely in cells, which is destabilized when the core dimerization domain, the B-box of CYLD, is deleted or disrupted.

### SPATA2 Activates CYLD

A number of USP domains are activated allosterically by binding partners (Sahtoe and Sixma, 2015). Recent structural insights into the USP46-UAF1 complex and the USP12-UAF1-WDR20 complex revealed that the activators interact with surfaces remote from the catalytic center and mediate activation via long-range allosteric mechanisms (Li et al., 2016, Yin et al., 2015).

SPATA2 also has a reproducible, yet moderate, activating effect on CYLD. Hydrolysis of Met1- or Lys63-linked tetraUb is enhanced in presence of SPATA2 (Figures 3E and S3F). Quantification of this effect employing fluorescent Met1/Lys63-linked diUb substrates (Keusekotten et al., 2013) reveals an ∼2-fold increase in *k*_*cat*_/*K*_*M*_ in presence of SPATA2 (Figures S3G and S3H). Deletion of the B-box does not affect the catalytic activity or structure of the CYLD USP domain (Komander et al., 2008, Sato et al., 2015), but SPATA2-mediated CYLD activation is lost in CYLD ΔB-box (Figure S3I) or in CYLD L622D (Figure 3F), as these CYLD variants no longer bind SPATA2. Likewise, mutations of SPATA2 residues in the CYLD interface decrease or abolish its ability to activate CYLD (Figures S3J–S3L). SPATA2 does not affect CYLD specificity, which remains Lys63 and Met1 specific at the diUb level (Figure S3M), and still does not significantly cleave Lys48-tetraUb (data not shown).

Together, this reveals a first function for SPATA2 in CYLD activation.

### SPATA2 Links CYLD to HOIP

**Figure 4:**
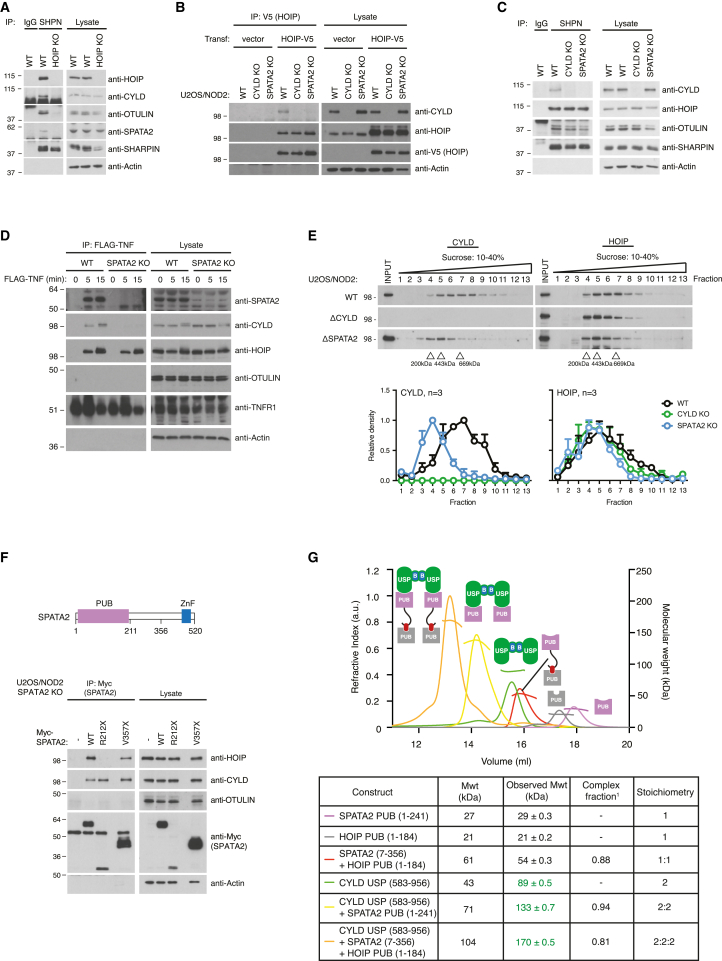
SPATA2 Binds CYLD and HOIP through Distinct Domains (A) Immunoprecipitation and western blot analysis of endogenous SHARPIN from WT or HOIP KO HCT116 cells. (B) Immunoprecipitation and western blot analysis of transiently expressed HOIP-V5 in WT, CYLD KO, and SPATA2 KO U2OS/NOD2 cells. (C) Immunoprecipitation and western blot analysis of endogenous SHARPIN in WT, CYLD KO, and SPATA2 KO U2OS/NOD2 cells. (D) Purification and western blot analysis of the TNFR1 complex from WT and SPATA2 KO U2OS/NOD2 cells stimulated with FLAG-TNF (100 ng/mL), as indicated using anti-FLAG agarose. (E) Sucrose gradient sedimentation and western blot analysis of sedimented fractions and lysates from WT, CYLD KO, and SPATA2 KO U2OS/NOD2 cells. Bottom, densitometry analysis of scanned immunoblots from three independent experiments. Values were normalized to the fraction with the highest density on each blot. Data represent means ± SEM. (F) Top, schematic representation of SPATA2 showing position for truncation variants R212X (1–211) and V357X (1–356). Bottom, immunoprecipitation and western blot analysis of transiently expressed Myc-SPATA2 variants from SPATA2 KO U2OS/NOD2 cells. (G) SEC-MALS analysis of indicated proteins and complexes on a Superdex 200 column. Bottom, table listing monomeric and observed MWs for each protein or complex, fraction of complex formed (see Figure 3A), and the resultant stoichiometry. Also see Figure S4.

We next investigated whether SPATA2 associates with LUBAC and would be involved in mediating CYLD recruitment. Indeed, purification of endogenous LUBAC by SHARPIN immunoprecipitation showed that SPATA2 co-immunoprecipitates with LUBAC and that the interaction is dependent on HOIP (Figure 4A), as is also the case for CYLD and OTULIN (Elliott et al., 2014, Hrdinka et al., 2016, Schaeffer et al., 2014, Takiuchi et al., 2014). Moreover, CYLD did not co-immunoprecipitate with ectopic V5-tagged HOIP or endogenous SHARPIN in SPATA2 KO cells, indicating that SPATA2 is required for the interaction of CYLD with LUBAC (Figures 4B and 4C). Purification of the TNFR1 complex with FLAG-tagged TNF recruits LUBAC (Haas et al., 2009) and, with it, CYLD (Draber et al., 2015). Importantly, the recruitment of CYLD to the TNFR1 complex depended on SPATA2 (Figure 4D).

To determine the amount of cellular CYLD associated with SPATA2 and LUBAC, we performed sucrose gradient sedimentation experiments on lysates from unstimulated WT, CYLD KO, and SPATA2 KO U2OS/NOD2 cells. This revealed that the majority of cellular CYLD sedimented along with HOIP in high MW fractions (with densitometry peak at 600–700 kDa) in WT cells (Figures 4E and S4A). Strikingly, in SPATA2 KO cells CYLD shifted to markedly lower MW fractions (with densitometry peak at ∼200 kDa). In accordance with the mass spectrometry experiments and the affinity of the CYLD and SPATA2 interaction, this suggests that a substantial fraction of the cellular pool of CYLD is in complex with SPATA2, which links it to LUBAC and possibly other high-order complexes. Although the sedimentation of HOIP was less influenced by the absence of SPATA2 (and CYLD), the highest MW HOIP complexes consistently shifted toward lower MW fractions in the SPATA2 KO and CYLD KO cells as compared with WT cells, which might reflect the loss of SPATA2 and/or CYLD from LUBAC complexes (Figures 4E and S4A).

We next determined how SPATA2 binds to HOIP. Expression of Myc-tagged SPATA2 variants in SPATA2 KO cells showed that while the PUB domain of SPATA2 (aa 1–211) did not bind HOIP, an extended construct of SPATA2 (aa 1–356) co-immunoprecipitated both CYLD and HOIP, indicating that the region following the PUB domain (aa 212–356) mediates the HOIP interaction (Figure 4F). This cellular binding study was confirmed in vitro with purified components. SEC-MALS with purified proteins showed that the interaction between SPATA2 (aa 7–356) and HOIP PUB (aa 1–184) is direct and that they form a 1:1 complex of 54 kDa (calculated 61 kDa) (Figure 4G, gray and red curves). Strikingly, the extended SPATA2 fragment formed a trimeric complex with CYLD and HOIP that eluted with an MW of 170 kDa, indicative of a stable 2:2:2 complex (Figure 4G, red, green, and orange curves; see Supplemental Experimental Procedures for details on stoichiometry calculation). This confirmed that SPATA2 is able to bridge CYLD with LUBAC via HOIP (Figure 4G).

While OTULIN, CYLD, and SPATA2 co-purified with LUBAC (Figure 4A), the association of OTULIN with LUBAC was independent of SPATA2 and CYLD (Figure 4C). Moreover, OTULIN was not co-purified with SPATA2, suggesting that SPATA2 (and CYLD) do not occupy the same HOIP/LUBAC molecules as OTULIN (Figure 4F).

### SPATA2 and HOIP Bind via a PIM-PUB Interaction

**Figure 5:**
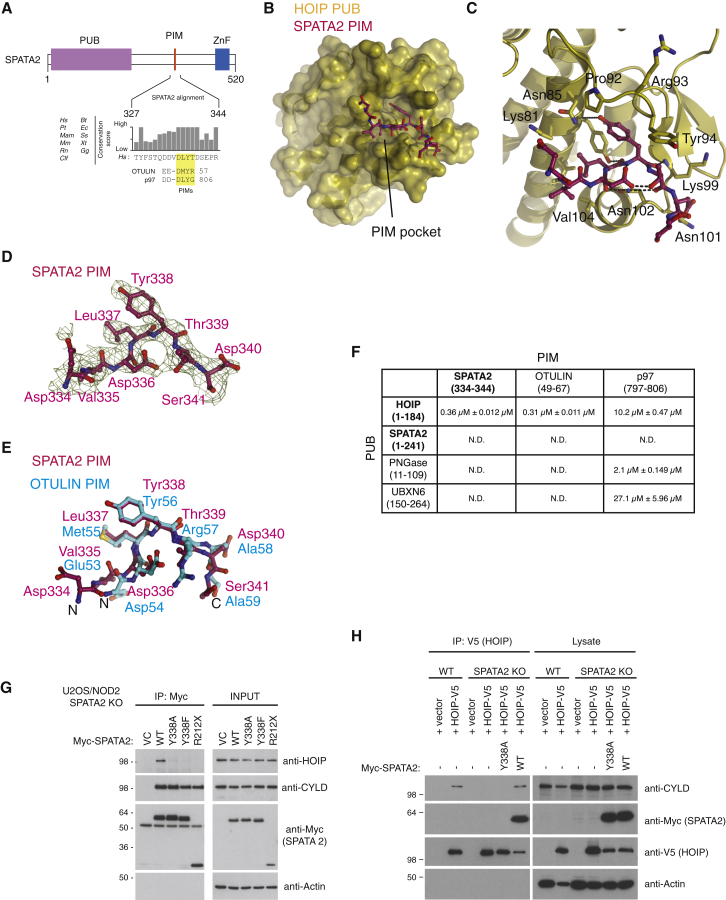
An HOIP-Specific PIM in SPATA2 (A) Primary sequence alignment of the HOIP PIM in SPATA2. The PIM is highly conserved among SPATA2 orthologs and aligns with the p97 and OTULIN PIMs. (B) Structure of the HOIP PUB domain (yellow surface) bound to the SPATA2 PIM (purple). (C) Close-up view of the HOIP PIM pocket with SPATA2 PIM bound. Key residues of the HOIP PUB domain are shown in sticks, and the SPATA2 PIM is shown in ball-and-stick representation. (D) A simulated annealing composite omit map contoured at 1 σ covering the SPATA2 PIM peptide. (E) Superposition of the SPATA2 PIM peptide (purple) with the OTULIN PIM (cyan). (F) Binding affinities of known PUB domains against known PIM peptides calculated by FP binding data using FITC-Ahx-labeled PIM peptides. Experiments were performed in triplicate and errors represent ± SEM. N.D., not detected. (G) Immunoprecipitation and western blot analysis of transiently expressed Myc-SPATA2 variants from SPATA2 KO U2OS/NOD2 cells. (H) Immunoprecipitation and western blot analysis of transiently expressed HOIP-V5 from WT and SPATA2 KO U2OS/NOD2 cells co-transfected with Myc-SPATA2 variants where indicated. Also see Figure S5.

It had been reported that mutation of the HOIP PUB domain “cornerstone” residue Asn102 in the PIM binding pocket (Elliott et al., 2014) abrogates binding not only to OTULIN but also to CYLD (Draber et al., 2015, Hrdinka et al., 2016, Takiuchi et al., 2014). This prompted us to speculate that SPATA2 might harbor a PIM and interacts with HOIP in a similar manner as does OTULIN. Indeed, species conservation analysis of the HOIP-interacting region in SPATA2 (aa 212–356) revealed a highly conserved putative PIM, Asp-Leu-Tyr-Thr, between amino acid residues 336 and 339, which is similar to the OTULIN PIM (Asp-Met-Tyr-Arg) (Elliott et al., 2014, Schaeffer et al., 2014) (Figures 5A and S2C).

A complex crystal structure of the HOIP PUB domain with a peptide spanning the putative SPATA2 PIM sequence (aa 334–344) at 2.7 Å confirmed binding of the SPATA2 PIM to the HOIP PIM pocket (Figures 5B–5D; Table 1). Two complexes in the asymmetric unit of the HOIP PUB-SPATA2 PIM crystals are highly similar (RMSD 0.7 Å) (Figure S5A), and while they crystallized in a distinct space group as compared to the HOIP PUB-OTULIN PIM crystals (*P*4_3_ as compared to *P*6_1_; Table 1), the PIM peptides overlay perfectly and occupy the PIM pocket in a virtually identical manner (Figure 5E). The major difference is the presence of Leu337 in SPATA2 instead of Met55 in OTULIN (Figure 5E), and, indeed, the SPATA2 PIM resembles the “original” PIM sequence derived from p97 (Asp-Leu-Tyr-Gly-COO^−^) (Elliott et al., 2014, Schaeffer et al., 2014, Zhao et al., 2007). In contrast to p97, both SPATA2 and OTULIN constitute internal PIMs, in which residues extending C terminally make additional contacts. In the SPATA2 PIM, Asp340 forms a backbone hydrogen bond with Asn101 in HOIP, mimicking contacts of the OTULIN PIM (Elliott et al., 2014, Schaeffer et al., 2014) (Figure 5C).

The structurally highly similar binding mode was also confirmed by biophysical binding measurements using fluorescently labeled PIM peptides and analyzing their binding to purified PUB domains by fluorescence polarization (FP) (Figures 5F and S5B–S5E). The SPATA2 or OTULIN PIM peptides displayed binding affinities of ∼300 nM with the HOIP PUB domain, but did not interact with PUB domains of PNGase or UBXN6 in this assay. The latter interacted only with the “terminal” PIM sequence found in p97, as reported (Elliott et al., 2014). Importantly, and consistent with our structural analysis described above (Figure 2), the SPATA2 PUB domain did not interact with any of the tested PIM peptides (Figures 5F and S5C).

In line with the in vitro analysis, substitution of the conserved Tyr338 in the SPATA2 PIM to Ala (Y338A) or Phe (Y338F) largely abrogated the interaction of SPATA2 with HOIP in cells, without affecting SPATA2 binding to CYLD (Figure 5G). Moreover, co-expression of V5-tagged HOIP and SPATA2 variants showed that the SPATA2 PIM is needed to co-immunoprecipitate CYLD and HOIP, demonstrating that the CYLD-SPATA2 complex is linked to LUBAC via the SPATA2 PIM-HOIP PUB interaction (Figure 5H).

### SPATA2 Links CYLD to Receptor Complexes and NF-κB Signaling

**Figure 6:**
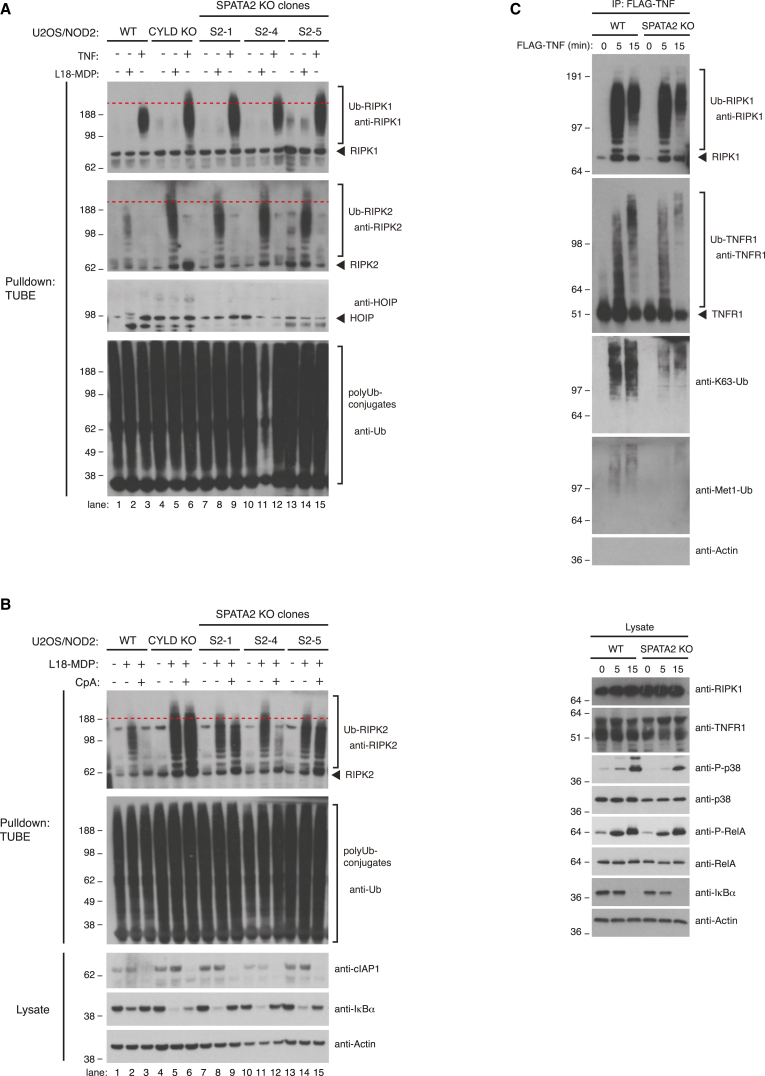
SPATA2 Mediates CYLD-Regulated NF-κB Responses (A and B) Purification and western blot analysis of endogenous Ub conjugates from WT, CYLD KO, and SPATA2 KO (clone S2-1, S2-4, and S2-5) U2OS/NOD2 cells. Cells were treated with (A) TNF (5 ng/mL for 10 min) or L18-MDP (200 ng/mL for 1 hr), or (B) were pretreated with DMSO (control) or 1 μM compound A (CpA) for 30 min before stimulation with L18-MDP (200 ng/mL for 1 hr) as indicated. (C) Purification and western blot analysis of the TNFR1 complex from WT and SPATA2 KO U2OS/NOD2 cells stimulated with FLAG-TNF (100 ng/mL) as indicated using anti-FLAG agarose. Also see Figure S6.

CYLD restricts ubiquitination of LUBAC substrates at receptor complexes to regulate inflammatory signaling (Draber et al., 2015, Hrdinka et al., 2016). We therefore explored the role of SPATA2 in regulating TNFR1 and NOD2 signaling. Strikingly, comparison of RIPK2 and RIPK1 ubiquitination in response to NOD2 stimulation and TNF treatment, respectively, showed that Ub-RIPK2 and Ub-RIPK1 species with a higher apparent MW accumulated in SPATA2 KO cells (three independent clones) and, as expected (Hrdinka et al., 2016), in CYLD KO cells as compared with WT cells (Figure 6A; RIPK2 blot, compare lane 2 with lanes 5, 8, 11, and 14; RIPK1 blot, compare lane 3 with lanes 6, 9, 12, and 15). To further assess the role of SPATA2 in regulating ubiquitination of RIPK2, cells were pre-treated with an Smac-mimetic compound (compound A, CpA), which inhibits the function of inhibitor of apoptosis (IAP) proteins and blocks RIPK2 ubiquitination in WT cells, but not in CYLD-depleted or CYLD KO cells (Damgaard et al., 2013, Hrdinka et al., 2016) (Figure 6B; compare lanes 3 and 6). Accordingly, RIPK2 ubiquitination was retained in SPATA2 KO cells despite CpA treatment, substantiating that SPATA2 regulates RIPK2 ubiquitination after NOD2 stimulation (Figure 6B; compare lane 3 with lanes 9, 12, and 15). Intriguingly, purification of the TNFR1 complex with FLAG-tagged TNF revealed that there was less Ub-RIPK1, Ub-TNFR1, Lys63-Ub, and Met1-Ub retained in the receptor complex in SPATA2 KO cells as compared with WT cells (Figures 6C and S6A). This suggests that SPATA2 not only regulates ubiquitination of LUBAC substrates but also contributes to the retention of ubiquitinated proteins at the TNFR1 complex.

**Figure 7:**
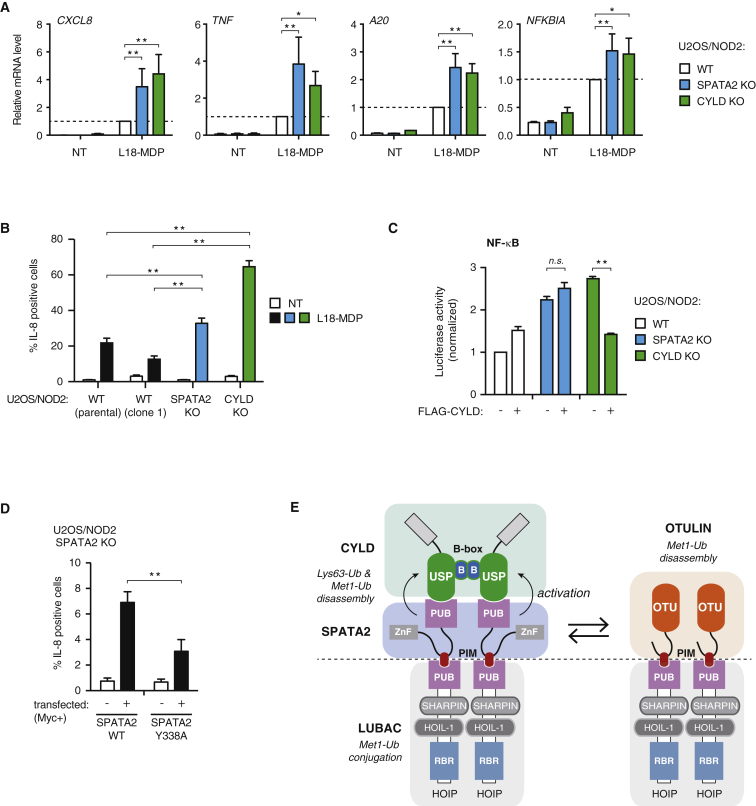
SPATA2 Mediates CYLD-Regulated NF-κB Responses (A) Relative levels of *CXCL8*, *TNF*, *A20*, and *NFKBIA* transcripts measured by qRT-PCR on cDNA from WT, CYLD KO, and SPATA2 KO U2OS/NOD2 cells treated with L18-MDP (200 ng/mL for 3 hr). (B) Intracellular flow cytometry analysis of IL-8 in WT (parental), WT (clone 1), SPATA2 KO, and CYLD KO U2OS/NOD2 cells. Cells were treated with L18-MDP (200 ng/mL) or vehicle for 5 hr along with Brefeldin A (5 μg/mL) and Monensin (2 μM) to block secretion of IL-8. (C) NF-κB activity in WT, CYLD KO, and SPATA2 KO U2OS/NOD2 cell lysates transfected with dual luciferase reporters and co-expressed with FLAG-CYLD as indicated. Luciferase activity is shown relative to the activity of WT U2OS/NOD2 cells transfected with vector control. Data represent the mean ± SEM of six independent experiments, each performed in duplicate. (D) Intracellular flow cytometry analysis of IL-8 in SPATA2 KO U2OS/NOD2 cells transfected with Myc-SPATA2 variants as indicated. Cells were treated with Brefeldin A (5 μg/mL) and Monensin (2 μM) for 5 hr. Data in (A), (B), and (D) represent mean ± SEM of at least three (A and B) or five (D) independent experiments. ^∗^p < 0.05, ^∗∗^p < 0.01; n.s., not significant. (E) Schematic representation of LUBAC complexes with the proposed configuration for binding of SPATA2/CYLD or OTULIN. Also see Figure S7.

Analysis of productive TNF signaling showed that loss of SPATA2, like CYLD, had little or no effect on activation of the MAP kinase p38, degradation of IκB, or phosphorylation of the NF-κB subunit RelA (Figure 6C) (Hrdinka et al., 2016). Accordingly, the expression of NF-κB response genes was similar in WT, SPATA2 KO, and CYLD KO cells following TNF treatment (Figure S7A). In contrast to this, NOD2 stimulation in SPATA2 KO cells and CYLD KO cells led to a comparable enhanced expression of NF-κB response genes as compared with WT cells, showing that SPATA2, like CYLD, restricts NOD2 signaling (Figure 7A). NOD2 stimulation also led to increased IL-8 production in SPATA2 KO cells as compared with WT cells, albeit the increase in IL-8 was less dramatic than in CYLD KO cells (Figure 7B).

We noted also that baseline NF-κB activity was elevated in both SPATA2 KO and CYLD KO cells as measured by a luciferase-based NF-κB reporter (Figure 7C). To directly address if SPATA2 mediates the ability of CYLD to regulate baseline NF-κB activity, we ectopically expressed CYLD in WT, CYLD KO, and SPATA2 KO cells. As expected, CYLD expression in CYLD KO cells reduced baseline NF-κB activity to the same level as in WT cells (Figure 7C). In contrast, CYLD had no effect on NF-κB activity in the SPATA2 KO cells, even though the expression level of CYLD was similar in all conditions (Figures 7C and S7B). This prompted us to address the function of the SPATA2 PIM in regulating productive NF-κB signaling. For this, IL-8 production was determined in SPATA2 KO cells transiently expressing SPATA2 WT or a PIM mutant (Y338A). Unexpectedly, transient overexpression of SPATA2 in SPATA2 KO cells (Figure S7C) caused spontaneous production of IL-8, which was largely dependent on the SPATA2 PIM (Figure 7D). This suggests that interaction of SPATA2 (and other PIM-containing proteins such as OTULIN) with the HOIP PUB is central to the regulation of LUBAC function and NF-κB signaling.

## Discussion

Here, we identify SPATA2 as a new regulatory factor of CYLD and as the protein that bridges CYLD to the Met1-Ub assembly machinery, LUBAC. The recent discovery that CYLD is associated with LUBAC revealed that the Ub-regulating capacity of LUBAC-DUB complexes extends beyond Met1-Ub to include Lys63-Ub and possibly other linkages (Draber et al., 2015, Hrdinka et al., 2016, Takiuchi et al., 2014). It is thus striking that the association of both DUBs with LUBAC is governed by PIMs (in OTULIN and SPATA2) that dock to the HOIP PUB domain in an identical manner. This raises the question if both DUBs can associate with LUBAC simultaneously or if distinct LUBAC-DUB complexes exist. Draber et al. showed that OTULIN and CYLD do not co-purify each other while both interact with LUBAC (Draber et al., 2015). In agreement with this, we reveal that CYLD-SPATA2 and OTULIN interact with distinct LUBAC complexes.

While the composition of LUBAC is currently not clear, at least two copies of HOIP exist in the complex (Elliott et al., 2014; data not shown); also, SHARPIN is dimeric (Stieglitz et al., 2012). We here show that CYLD is a constitutive dimer in solution and that it forms a 2:2 complex with SPATA2. This places two SPATA2 PIMs in close proximity, and the hetero-tetramer binds two copies of HOIP, likely invoking favorable avidity effects. Intriguingly, deletion of SPATA2 did not lead to increased binding of OTULIN to LUBAC, which suggests that the amount of OTULIN available for HOIP binding is limited. In contrast, we had previously observed that OTULIN levels seem to be in excess of HOIP, but only a fraction of it is bound to LUBAC, possibly due to phosphorylation of the PIM (Elliott et al., 2014, Zhao et al., 2007).

Importantly, which DUB is associated has regulatory implications. OTULIN binding to HOIP is required to restrict LUBAC auto-ubiquitination, which is readily detected on HOIP, HOIL-1, and SHARPIN when OTULIN is depleted or mutated in its PIM (Draber et al., 2015, Elliott et al., 2014, Fiil et al., 2013, Hrdinka et al., 2016, Keusekotten et al., 2013). In contrast, CYLD association with LUBAC appears to predominantly regulate LUBAC substrate ubiquitination such as RIPK2 after NOD2 stimulation and components of the active TNFR1 complex (Draber et al., 2015, Hrdinka et al., 2016).

Thus, we propose a dynamic model where OTULIN and CYLD-SPATA2 are interchanged at the HOIP PUB interface, whereby the DUBs do not stably co-exist at the same LUBAC complex (Figure 7E). Quantitative studies of native LUBAC complexes will be needed to fully elucidate their composition and how DUB occupancy, and thereby LUBAC function, is regulated.

Our study reveals that the CYLD B-box, the role of which has remained elusive, is responsible for dimerization of the CYLD USP domain. HOIP contains a CYLD-like B-box following the PUB domain (Komander et al., 2008), and we have previously shown that a fragment spanning PUB, B-Box, and subsequent zinc-finger domains is able to self-associate (Elliott et al., 2014). B-box modules are also present in E3 ligases of the tripartite motif (TRIM) family, where at least in some cases they contribute to protein oligomerization (Wagner et al., 2016a). A crystal structure of the B-box from TRIM63/MuRF1 indicates a hydrophobic dimer interface as seen in CYLD (Mrosek et al., 2008). It will be interesting to see if the ability to form homotypic interactions is a general feature of B-box modules.

Strikingly, SPATA2 did not interact with monomeric CYLD ΔB-box, and while the CYLD-SPATA2 binding interface does not appear to involve the B-box itself, SPR measurements reveal diminished complex stability when CYLD is monomeric. It is possible that SPATA2 binding to a CYLD dimer creates additional interactions that stabilize the hetero-tetrameric complex, and further structural work is required to illuminate this.

Finally, the finding that SPATA2 activates CYLD adds SPATA2 to the growing list of allosteric DUB activators. Recent structural work revealed how the WD-repeat protein UAF1 interacted with the catalytic domain of USP46 and USP12, which is facilitated predominantly through the “fingers” subdomain of the USP core, mediating long-range allosteric interactions eventually leading to enhanced enzyme efficiency (Yin et al., 2015, Li et al., 2016). CYLD is structurally distinct from canonical USP domain folds in that it does not contain “fingers” but instead a B-box that binds the exposed side of the palm domain, but removal of which does not affect CYLD activity (Komander et al., 2008, Sato et al., 2015). The location of the SPATA2 binding site at the back of the palm domain suggests that SPATA2 activates CYLD in a distinct manner. In addition to SPATA2, SPATA2L also interacts with CYLD in cells (Sowa et al., 2009) (Figure 1E; Table S1) and may regulate other aspects of CYLD function. The fact that KO of SPATA2 already prevents association of CYLD with the TNFR1 (Figure 4D) indicates that SPATA2L cannot simply substitute for SPATA2, at least in the cellular systems tested.

In line with the biochemical data, our functional data indicate that SPATA2 plays a central regulatory role in LUBAC-mediated signaling, in particular in response to NOD2 stimulation, where loss of SPATA2 resulted in increased RIPK2 ubiquitination and productive NOD2 signaling. Moreover, it was striking that SPATA2 overexpression led to spurious IL-8 production in an SPATA2 PIM-dependent manner. Loss of SPATA2, however, had a less dramatic effect on some NOD2 responses than did the loss of CYLD. This could reflect that CYLD can regulate signaling independently of LUBAC binding, but that remains to be investigated. In addition to this, SPATA2 regulated ubiquitination following TNF treatment, and while this did not appear to greatly affect NF-κB signaling, it could well affect the formation of cell death-inducing complexes, as is the case for CYLD (Hitomi et al., 2008, O’Donnell et al., 2011). Indeed, during revision of this manuscript a study by Wagner et al. (2016b) showed that SPATA2 mediates necroptosis induced by TNF and caspase inhibition.

Conceptually, our study reveals that PUB-PIM interactions are at the core of how LUBAC function is regulated through its associated DUB activities, CYLD-SPATA2 and OTULIN. This adds SPATA2 to the still growing list of regulatory components of LUBAC and identifies a new modulator of important pathways involved in inflammation and infection. Future studies into the regulation of these interactions will be important for our understanding of how Met1-Ub regulates cellular signaling.

## Experimental Procedures

Please see the Supplemental Experimental Procedures for further details on all experimental procedures.

### Protein Expression and Purification

HOIP and SPATA2 proteins were purified from E.coli as described (Elliott et al., 2014), and CYLD was expressed in Sf9 insect cells as described (Komander et al., 2008).

### Crystallization and Structure Determination

Crystallization was performed using sparse matrix sitting drop vapor diffusion screening. The SPATA2 structure was determined by AMPLE (Bibby et al., 2012) using idealized helices, and the HOIP PUB-SPATA2 PIM complex by molecular replacement.

### Binding Experiments

Details on gel filtration studies, SPR, and FP experiments can be found in the Supplemental Experimental Procedures.

### Mass Spectrometry

Details on LC-MS/MS analysis of CYLD interactors can be found in the Supplemental Experimental Procedures.

### DUB Assays

Qualitative gel-based DUB assays were performed as in Komander et al. (2009). Quantitative cleavage of Lys63-/Met1-linked diUb using fixed concentrations of FlAsH-labeled diUb was performed as in Keusekotten et al. (2013).

### Purification of Endogenous Ub Conjugates

Ub conjugates were purified using GST-1xUBA^ubq^ Ub affinity reagent as in Fiil et al. (2013).

### Characterization of NOD2 and TNF Signaling

Flow cytometry analysis of IL-8 production, quantitative real-time PCR, and purification of TNFR complexes were performed as described in Hrdinka et al. (2016).

### Luciferase Reporter Assays

Cells were co-transfected the NF-κB luciferase reporter construct pBIIX-luc and a thymidine kinase-renilla luciferase construct. Additional plasmids were transfected as indicated and assays performed as in Damgaard et al. (2012).

### Sucrose Gradient Sedimentation

Continuous 10%–40% sucrose gradients were generated using a GradientMaster 108 (Biocomp). Whole-cell lysate was subjected to velocity sedimentation on the sucrose gradients and fractioned before analysis by immunoblotting.

### Generation of CRISPR/Cas9 Cell Lines

KO cell lines were created with the CRISPR/Cas9 KO (Santa Cruz) system containing gRNA, Cas9, and EGFP marker.

## Author Contributions

Conceptualization, M.G.-H. and D.K.; Investigation, P.R.E., D.L., M.H., K.B., J.W., S.M.V.F., B.K.F., S.H.M., and N.V.; Methodology, B.M.K. and J.C.C.; Writing – Original Draft, M.G.-H., D.K., P.R.E., and D.L.; Writing, M.G.-H., D.K., P.R.E., and D.L.; Funding Acquisition, M.G.-H. and D.K.

## Figures and Tables

**Table 1:** Data Collection and Refinement Statistics

	SPATA2 7–219	HOIP 5–180 + SPATA2 334–344
**Data Collection**		

Beamline	Diamond I02	Diamond I02
Space group	*P 2*_*1*_	*P 4*_*3*_
*a*, *b*, *c* (Å)	43.48, 51.14, 56.29	89.04, 89.04, 53.56
α, β, γ (°)	90.00, 105.97, 90.00	90.00, 90.00, 90.00
Wavelength	0.9794	0.9795
Resolution (Å)	54.12–1.45 (1.48–1.45)	62.96–2.70 (2.83–2.70)
*R*_merge_	3.7 (32.7)	7.2 (67.9)
*< I* / σ*I >*	12.1 (2.2)	9.5 (2.0)
CC(1/2)	0.99 (0.91)	0.99 (0.61)
Completeness (%)	90.2 (81.2)	97.7 (99.7)
Redundancy	3.0 (2.9)	2.7 (2.7)

**Refinement**		

Resolution (Å)	54.11–1.45	62.96–2.70
No. reflections	37,458	11,425
*R*_work_ / *R*_free_	18.2/22.5	23.3/28.1

**No. Atoms**		

Protein	1,722	2,695
Ligand/ion	12	15
Water	308	–

**B Factors**		

Wilson B	15.73	57.1
Protein	25.1	61.3
Ligand/ion	21.8	85.0
Water	38.0	–

**RMSDs**		

Bond lengths (Å)	0.005	0.002
Bond angles (°)	0.772	0.526
Ramachandran statistics (outliers, allowed, favored)	0.0, 1.4, 98.6	0.0, 2.6, 97.4

Related to Figures 2 and 5. Values in parentheses are for the highest-resolution shell. Datasets were collected and structures determined from a single crystal.

## Accession Numbers

The accession numbers for the coordinates and structure factors for the SPATA2 PUB domain and HOIP PUB- SPATA2 PIM structures are PDB: 5LJM and 5LJN, respectively.

## Supplemental Information

Document S1. Supplemental Experimental Procedures and Figures S1–S7

Table S1. Mass Spectrometry Analysis of CYLD Interactome

Document S2. Article plus Supplemental Information
