# Supplementary material for: SPATA2 Links CYLD to LUBAC, Activates CYLD, and Controls LUBAC Signaling
Source: Mol Cell. 2016 Sep 15;63(6):990–1005. doi: 10.1016/j.molcel.2016.08.001 (PMC5031558; doi:10.1016/j.molcel.2016.08.001)
Supplement: Document S1. Supplemental Experimental Procedures and Figures S1–S7 [file mmc1.pdf]

Molecular Cell, Volume *63*

Supplemental Information

SPATA2 Links CYLD to LUBAC, Activates CYLD, and Controls LUBAC Signaling

Paul R. Elliott, Derek Leske, Matous Hrdinka, Katrin Bagola, Berthe K. Fiil, Stephen H. McLaughlin, Jane Wagstaff, Norbert Volkmar, John C. Christianson, Benedikt M. Kessler, Stefan M.V. Freund, David Komander, and Mads Gyrd-Hansen

Figure S1

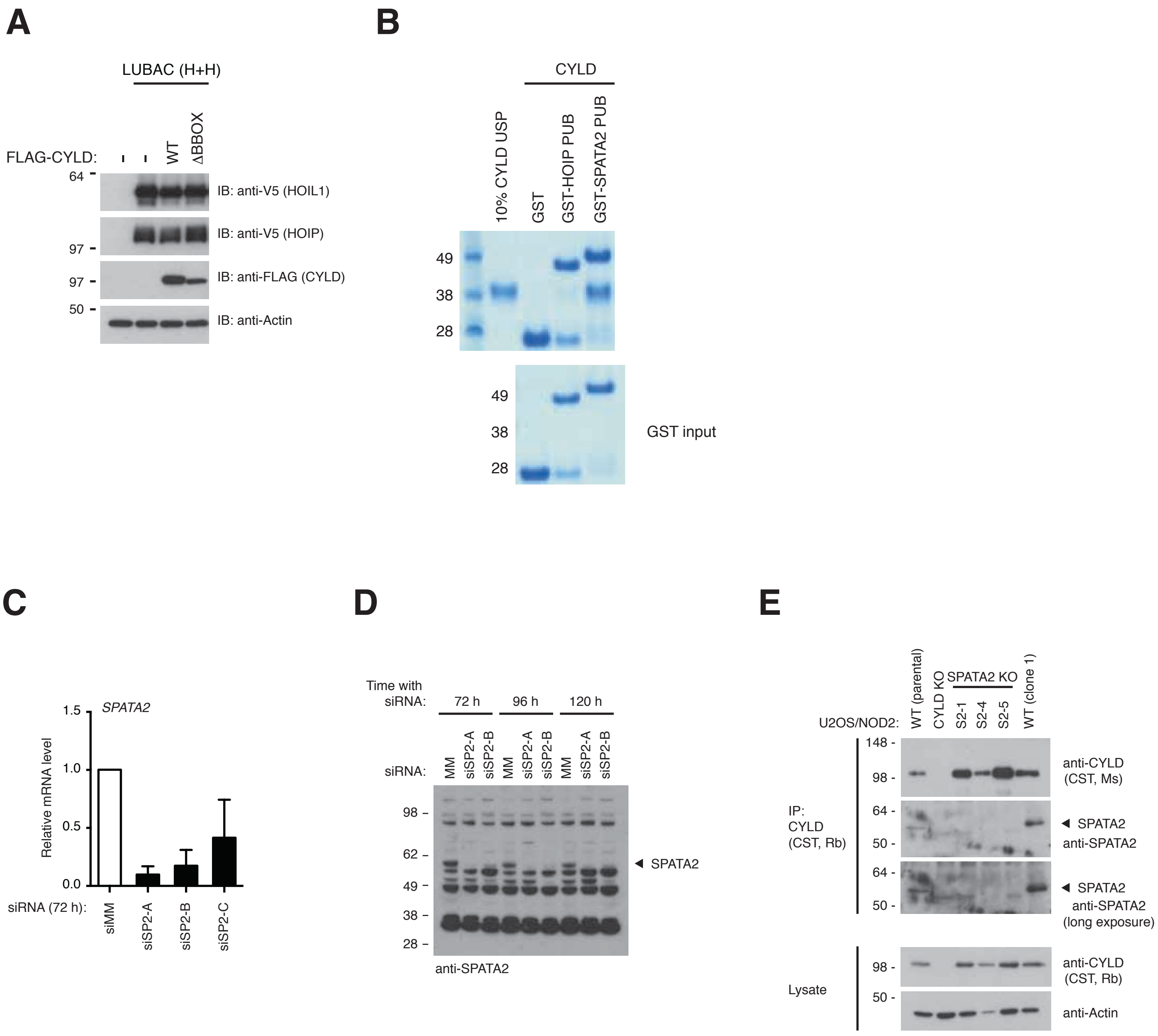
A
LUBAC (H+H)
FLAG-CYLD:
WT
ΔBBOX
64
97
97
50
IB: anti-V5 (HOIL1)
IB: anti-V5 (HOIP)
IB: anti-FLAG (CYLD)
IB: anti-Actin
B
CYLD
10% CYLD USP
GST
GST-HOIP PUB
GST-SPATA2 PUB
49
38
28
GST input
C
SPATA2
Relative mRNA level
1.5
1.0
0.5
0.0
siRNA (72 h):
siMM
siSP2-A
siSP2-B
siSP2-C
D
Time with siRNA:
72 h
96 h
120 h
siRNA:
MM
siSP2-A
siSP2-B
98
62
49
38
28
SPATA2
anti-SPATA2
E
U2OS/NOD2:
WT (parental)
CYLD KO
SPATA2 KO
S2-1
S2-4
S2-5
WT (clone 1)
IP: CYLD (CST, Rb)
148
98
64
50
anti-CYLD (CST, Ms)
SPATA2
anti-SPATA2
anti-SPATA2 (long exposure)
Lysate
anti-CYLD (CST, Rb)
anti-Actin

Figure S1:, related to Figure 1
(A) CYLD KO U2OS/NOD2 cell lysates from experiment shown in Figure 1C were analyzed by western blot analysis as indicated. (B) Coomassie stained GST pull-down experiments using GST, GST-HOIP PUB or GST-SPATA2 PUB and CYLD USP domain. (C-E) Characterization of SPATA2 antibody used in study and confirmation of knockout U2OS/NOD2 cell lines generated using CRISPR/Cas9. (C) Relative *SPATA2* mRNA levels following knockdown with *SPATA2* siRNA (SP2-A, SP2-B, and SP2-C) in U2OS/NOD2 cells. Values shown represents mean ± SEM from three independent experiments. (D) Western blot analysis of SPATA2 levels in U2OS/NOD2 cell lysates following knockdown by MM (control), SP2-A, and SP2-B siRNA for 72 h, 96 h, and 120 h (as indicated). (E) Immunoprecipitation and western blot analysis of endogenous CYLD in WT, CYLD KO, SPATA2 KO clones (S2-1, S2-4, and S2-5), and S3-5 (selected WT clone) to determine SPATA2 status.

Figure S2

A

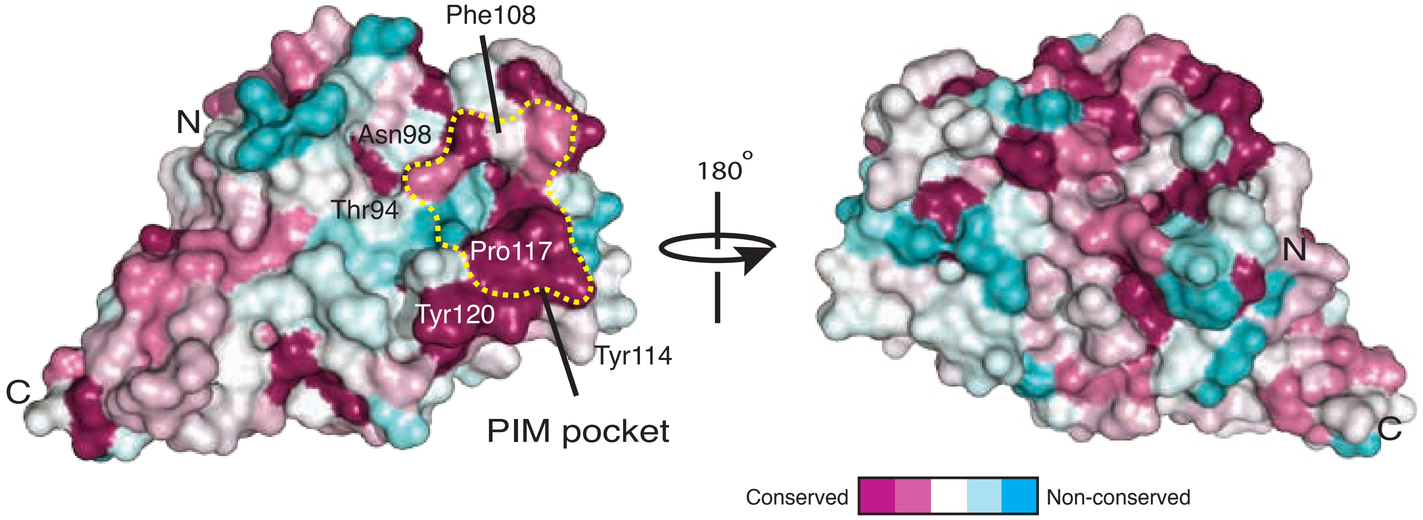

B

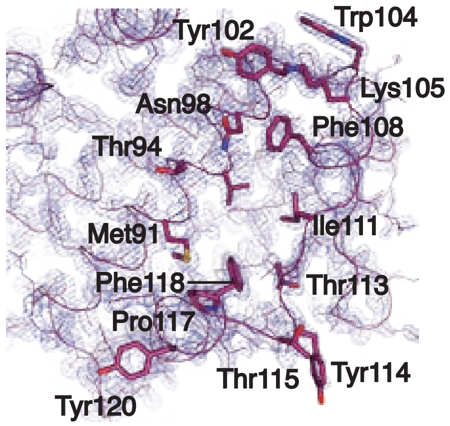

C

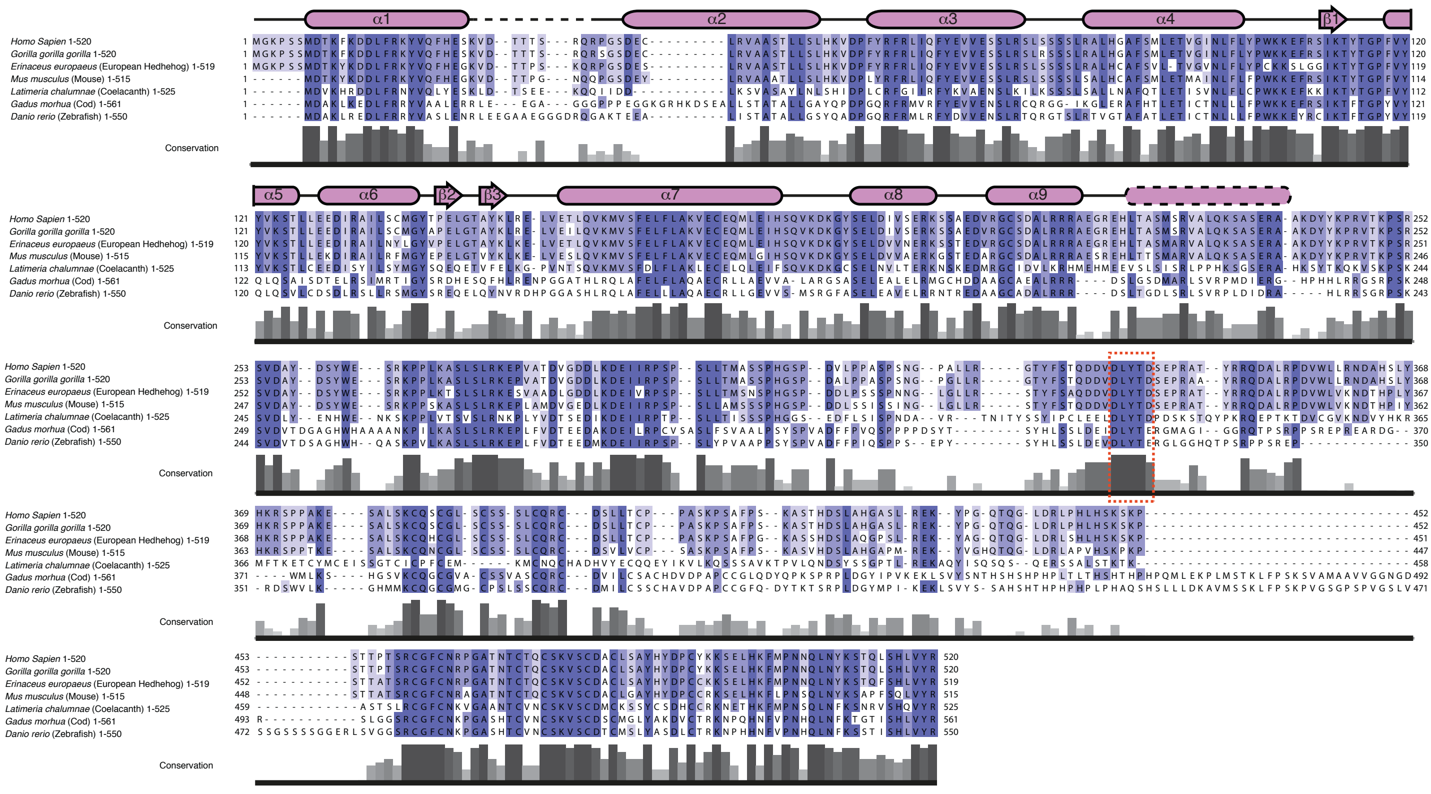

Figure S2:, related to Figure 2

(A) Surface conservation analysis of the SPATA2 PUB domain colored according to sequence conservation. The PIM pocket is highlighted, as are key residues around the PIM pocket and CYLD binding site. (B) A weighted 2|Fo|-|Fc| map contoured at 0.9 σ of the PIM pocket of SPATA2. (C) Sequence alignment of SPATA2 showing the sequences of distant orthologs. The sequence conservation between all SPATA2 orthologs is shown as bars. Secondary structure of the PUB domain is shown above the alignment. The PIM peptide is shown (red box). A predicted tenth alpha helix based upon secondary structure analysis is shown where the longer SPATA2 PUB domain (1-241) ends. This fragment behaves better during purification and is used in biophysical analysis.

Figure S3

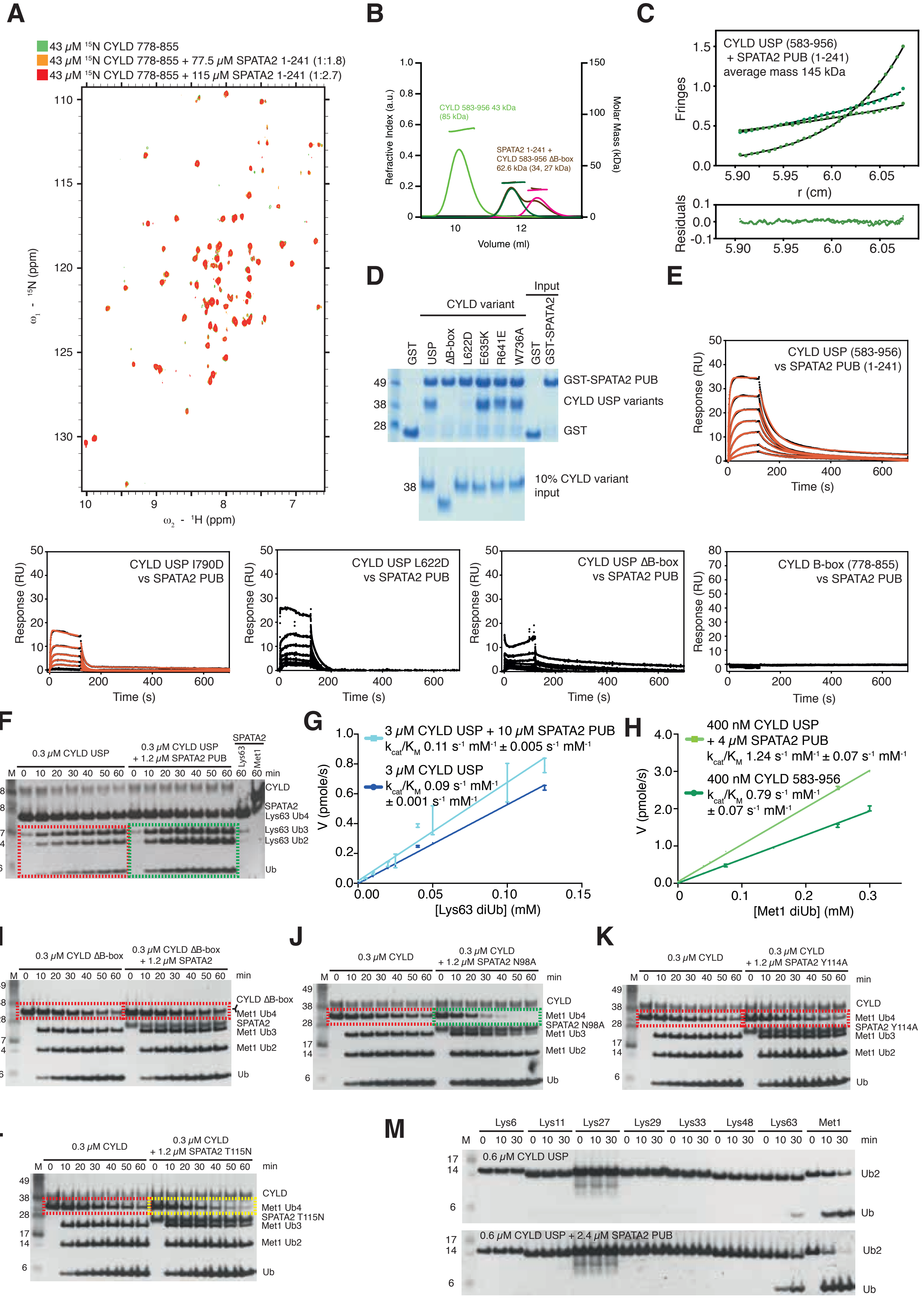

A
43 μM ¹⁵N CYLD 778-855
43 μM ¹⁵N CYLD 778-855 + 77.5 μM SPATA2 1-241 (1:1.8)
43 μM ¹⁵N CYLD 778-855 + 115 μM SPATA2 1-241 (1:2.7)
ω1 - ¹⁵N (ppm)
ω2 - ¹H (ppm)
B
CYLD 583-956 43 kDa (85 kDa)
SPATA2 1-241 + CYLD 583-956 ΔB-box 62.6 kDa (34, 27 kDa)
Refractive Index (a.u.)
Molar Mass (kDa)
Volume (ml)
C
CYLD USP (583-956) + SPATA2 PUB (1-241) average mass 145 kDa
Fringes
r (cm)
Residuals
D
CYLD variant
Input
GST
USP
ΔB-box
L622D
E635K
R641E
W736A
GST
GST-SPATA2
GST-SPATA2 PUB
CYLD USP variants
GST
10% CYLD variant input
E
CYLD USP (583-956) vs SPATA2 PUB (1-241)
Response (RU)
Time (s)
CYLD USP I790D vs SPATA2 PUB
CYLD USP L622D vs SPATA2 PUB
CYLD USP ΔB-box vs SPATA2 PUB
CYLD B-box (778-855) vs SPATA2 PUB
F
0.3 μM CYLD USP
0.3 μM CYLD USP + 1.2 μM SPATA2 PUB
SPATA2
Lys63
Met1
M 0 10 20 30 40 50 60 0 10 20 30 40 50 60 60 60 min
CYLD
SPATA2
Lys63 Ub4
Lys63 Ub3
Lys63 Ub2
Ub
G
3 μM CYLD USP + 10 μM SPATA2 PUB
kcat/KM 0.11 s⁻¹ mM⁻¹ ± 0.005 s⁻¹ mM⁻¹
3 μM CYLD USP
kcat/KM 0.09 s⁻¹ mM⁻¹ ± 0.001 s⁻¹ mM⁻¹
V (pmole/s)
[Lys63 diUb] (mM)
H
400 nM CYLD USP + 4 μM SPATA2 PUB
kcat/KM 1.24 s⁻¹ mM⁻¹ ± 0.07 s⁻¹ mM⁻¹
400 nM CYLD 583-956
kcat/KM 0.79 s⁻¹ mM⁻¹ ± 0.07 s⁻¹ mM⁻¹
V (pmole/s)
[Met1 diUb] (mM)
I
0.3 μM CYLD ΔB-box
0.3 μM CYLD ΔB-box + 1.2 μM SPATA2
CYLD ΔB-box
Met1 Ub4
SPATA2
Met1 Ub3
Met1 Ub2
Ub
J
0.3 μM CYLD
0.3 μM CYLD + 1.2 μM SPATA2 N98A
CYLD
Met1 Ub4
SPATA2 N98A
Met1 Ub3
Met1 Ub2
Ub
K
0.3 μM CYLD
0.3 μM CYLD + 1.2 μM SPATA2 Y114A
CYLD
Met1 Ub4
SPATA2 Y114A
Met1 Ub3
Met1 Ub2
Ub
L
0.3 μM CYLD
0.3 μM CYLD + 1.2 μM SPATA2 T115N
CYLD
Met1 Ub4
SPATA2 T115N
Met1 Ub3
Met1 Ub2
Ub
M
Lys6 Lys11 Lys27 Lys29 Lys33 Lys48 Lys63 Met1
M 0 10 30 0 10 30 0 10 30 0 10 30 0 10 30 0 10 30 0 10 30 0 10 30 min
0.6 μM CYLD USP
0.6 μM CYLD USP + 2.4 μM SPATA2 PUB
Ub2
Ub

Figure S3:, related to Figure 3

(A) BEST-TROSY spectra of ^{15}N-labelled CYLD B-box (778-855) (green) mixed with 1:1.8 molar excess (orange) and 1:2.7 molar excess of SPATA2 PUB domain (red), showing no perturbations of B-box resonances, indicative of no or very weak binding. (B) SEC-MALS profile for CYLD USP (green), CYLD USP ΔB-box (blue), SPATA2 PUB (red) and CYLD USP ΔB-box mixed with SPATA2 PUB (brown). The separate elution profiles of the USP ΔB-box and SPATA2 PUB domain are in agreement with the elution profiles and molecular weights of individual components, confirming lack of complex formation. (C) Sedimentation equilibrium analytical ultracentrifugation analysis of the CYLD USP–SPATA2 PUB complex. 1 μM of complex was centrifuged at 5,700, 6,900 and 12,000 rpm at 20 °C until equilibration was reached. The fringe pattern is plotted against the position within the cell and the residuals are shown below. The calculated Mwt of the complex is 145 kDa, consistent with a 2:2 dimer (predicted Mwt 142 kDa). (D) Pull-down using GST-SPATA2 PUB and different CYLD variants. (E) SPR sensorgrams for CYLD variant binding to immobilized SPATA2 PUB. Fits of the raw data for determining the kinetic dissociation constant (K^{kin}_{d}) are shown in red. (F) DUB assay, similar to **Figure 3E**, following Lys63-linked tetra-Ub cleavage by CYLD in the absence or presence of SPATA2 PUB domain. The final two lanes demonstrate that SPATA2 does not contain any protease activity against Lys63- and Met1-linked tetraUb. A small amount of contaminating Lys63-linked di- and triUb are found in the Lys63-linked tetraUb sample at time zero and does not increase overtime unless CYLD is present. (G-H) Kinetic analysis using Lys63-linked diUb (G) or Met1-linked diUb (H) and a fixed concentration of 150 nM Lys63/Met1 FlAsH-tagged diUb. Initial rates of substrate cleavage are plotted against substrate concentration and fitted to yield CYLD activity in the absence or presence of SPATA2 PUB. Error bars represent standard deviation from the mean of measurements performed in triplicate. (I) DUB assay similar to **Figures 3E-F,** using CYLD USP ΔB-box. Removal of the B-box domain does not affect CYLD DUB activity. However, CYLD ΔB-box is unable to be activated by SPATA2 PUB. (J-L) DUB assays, using Met1-linked tetraUb and SPATA2 PUB mutant that still binds and activates CYLD (N98A, J), abolish CYLD binding and activation (Y114A, K) and have an intermediate effect on CYLD activation (T115N, L). (M) DUB assay using 0.6 μM CYLD USP and all eight linked diUb in the absence of (top) and the presence of 2.4 μM SPATA2 (bottom) showing an enhancement in CYLD activity by SPATA2 but no change in Ub linkage specificity by CYLD.

Figure S4

A

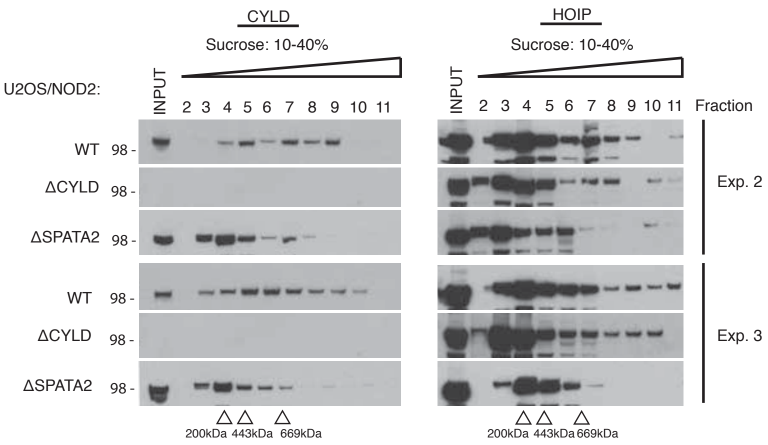

CYLD
Sucrose: 10-40%
HOIP
Sucrose: 10-40%
U2OS/NOD2:
INPUT
2 3 4 5 6 7 8 9 10 11
INPUT
2 3 4 5 6 7 8 9 10 11 Fraction
WT 98 -
ΔCYLD 98 -
ΔSPATA2 98 -
Exp. 2
WT 98 -
ΔCYLD 98 -
ΔSPATA2 98 -
Exp. 3
200kDa 443kDa 669kDa
200kDa 443kDa 669kDa

Figure S4:, related to Figure 4
(A) Sucrose centrifugation sedimentation (experiments two and three) and western blot analysis of lysates from WT, CYLD KO, and SPATA2 KO U2OS/NOD2 cells as in **Figure 4E**. The shown blots together with the blots in Figure 4E were subjected to image densitometry using ImageJ software. Raw values in each fraction were normalized and means ± SEM are depicted in Figure 4E, bottom.

Figure S5

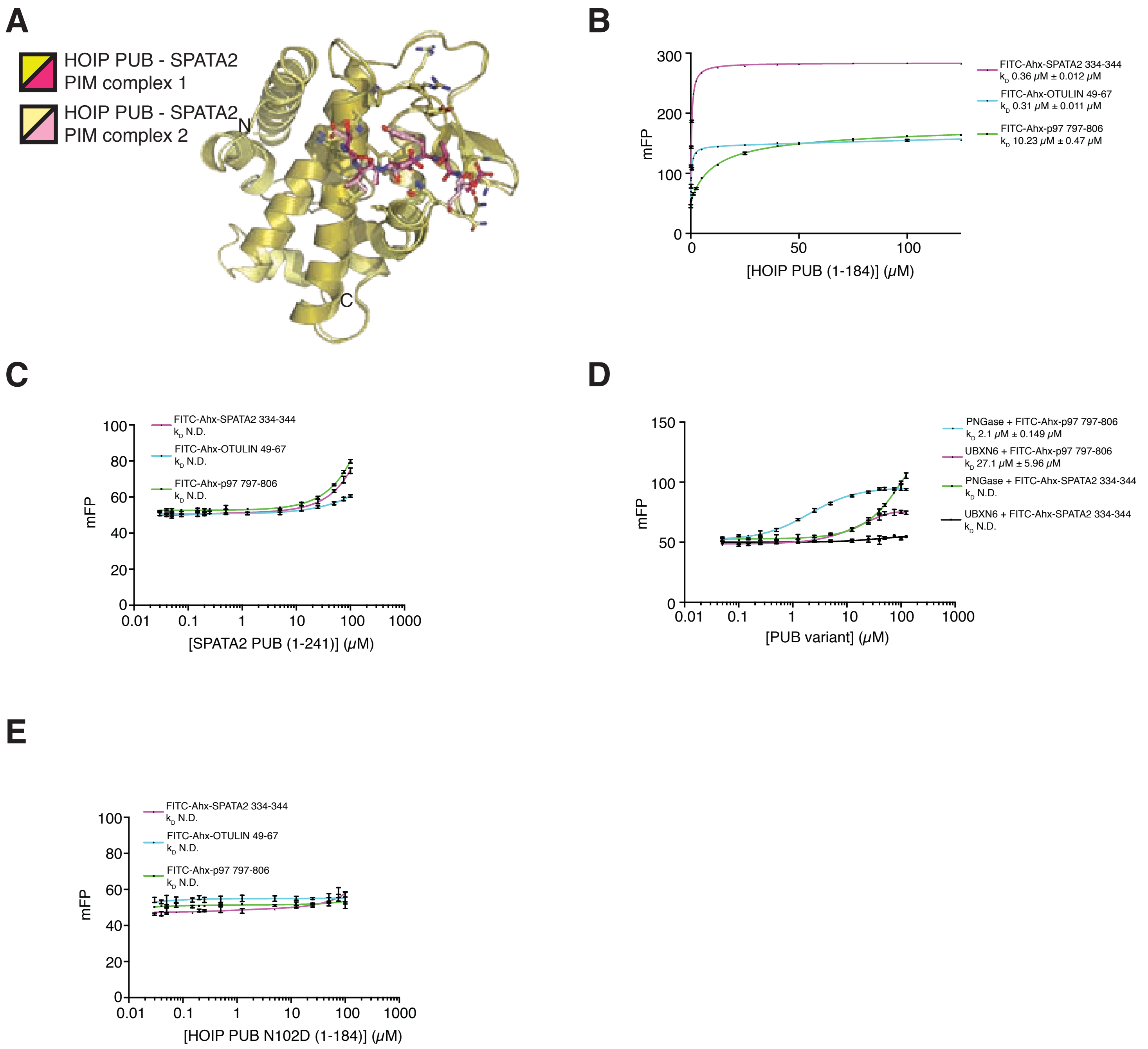
A
HOIP PUB - SPATA2 PIM complex 1
HOIP PUB - SPATA2 PIM complex 2
N
C
B
mFP
[HOIP PUB (1-184)] (μM)
FITC-Ahx-SPATA2 334-344 k_D 0.36 μM ± 0.012 μM
FITC-Ahx-OTULIN 49-67 k_D 0.31 μM ± 0.011 μM
FITC-Ahx-p97 797-806 k_D 10.23 μM ± 0.47 μM
C
mFP
[SPATA2 PUB (1-241)] (μM)
FITC-Ahx-SPATA2 334-344 k_D N.D.
FITC-Ahx-OTULIN 49-67 k_D N.D.
FITC-Ahx-p97 797-806 k_D N.D.
D
mFP
[PUB variant] (μM)
PNGase + FITC-Ahx-p97 797-806 k_D 2.1 μM ± 0.149 μM
UBXN6 + FITC-Ahx-p97 797-806 k_D 27.1 μM ± 5.96 μM
PNGase + FITC-Ahx-SPATA2 334-344 k_D N.D.
UBXN6 + FITC-Ahx-SPATA2 334-344 k_D N.D.
E
mFP
[HOIP PUB N102D (1-184)] (μM)
FITC-Ahx-SPATA2 334-344 k_D N.D.
FITC-Ahx-OTULIN 49-67 k_D N.D.
FITC-Ahx-p97 797-806 k_D N.D.

Figure S5:, related to Figure 5

(A) Superimposition of the two HOIP PUB – SPATA2 PIM structures from the asymmetric unit. (B) Affinity measurements using HOIP PUB domain against FITC-Ahx-p97 797-806 (green), FITC-Ahx-OTULIN 49-67 (cyan) and FITC-Ahx-SPATA2 334-344 (magenta). (C) Affinity measurements as in (B) using SPATA2 PUB domain. (D) Affinity measurements using either PUB domains of PNGase or UBXN6. (E) Mutation of the cornerstone residue Asn102 in HOIP abolishes all PIM binding, including SPATA2. All measurements were performed in triplicate and errors represent the standard deviation from the mean.

Figure S6

A

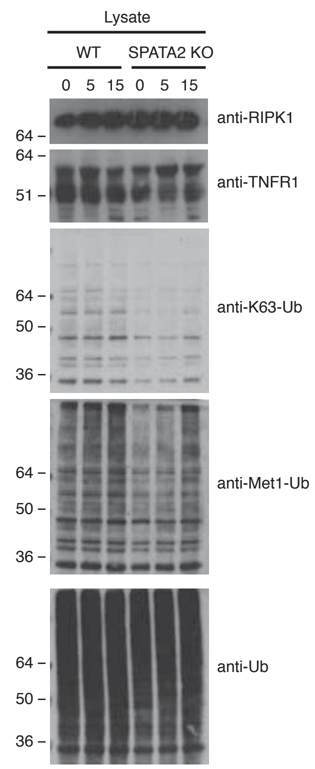
Lysate
WT
SPATA2 KO
0 5 15 0 5 15
64 –
anti-RIPK1
64 –
51 –
anti-TNFR1
64 –
50 –
36 –
anti-K63-Ub
64 –
50 –
36 –
anti-Met1-Ub
64 –
50 –
36 –
anti-Ub

Figure S6:, related to Figure 6

(A) WT and SPATA2 KO U2OS/NOD2 cell lysates from FLAG-TNF immunoprecipitation shown in **Figure 6A** were analyzed by western blot as indicated.

Figure S7

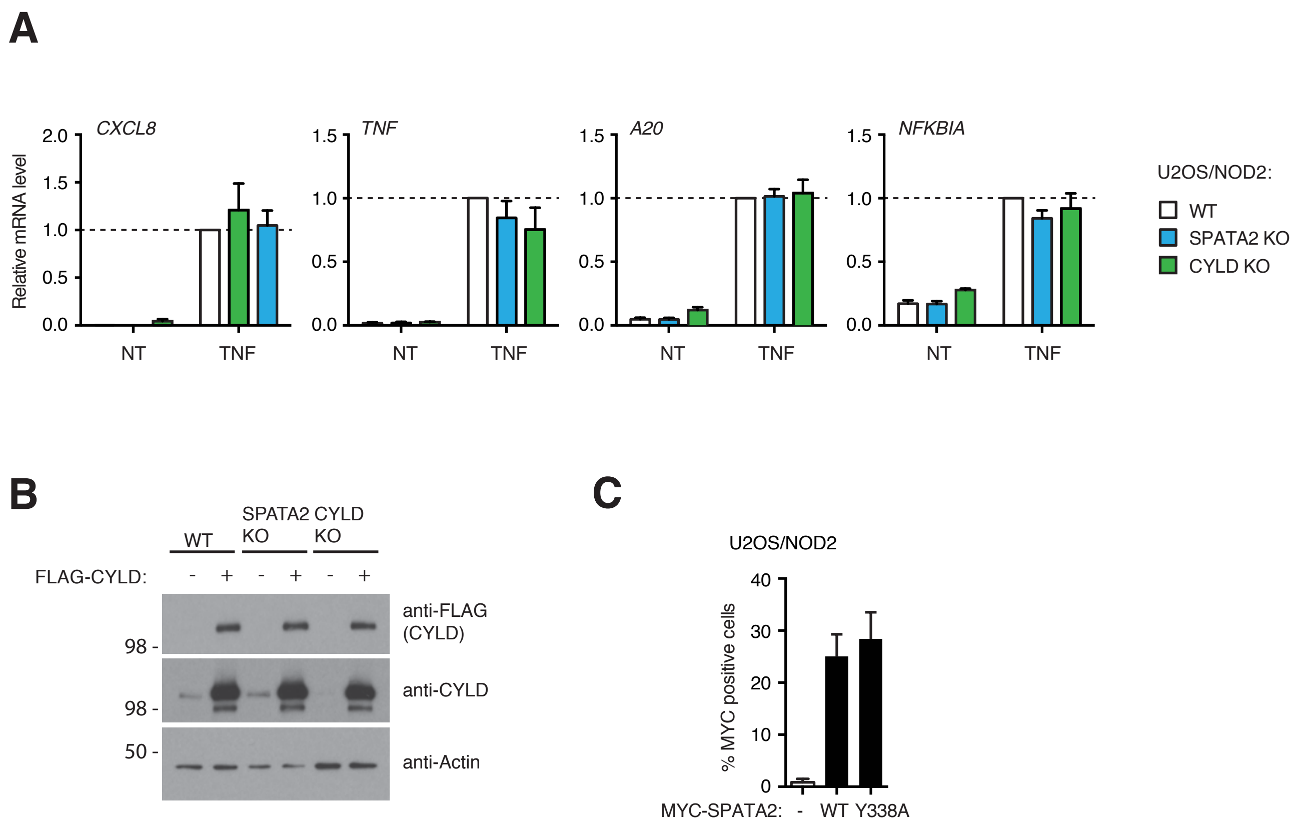
A
CXCL8
TNF
A20
NFKBIA
Relative mRNA level
2.0
1.5
1.0
0.5
0.0
NT
TNF
U2OS/NOD2:
WT
SPATA2 KO
CYLD KO
B
WT
SPATA2 KO
CYLD KO
FLAG-CYLD:
- + - + - +
98 -
98 -
50 -
anti-FLAG (CYLD)
anti-CYLD
anti-Actin
C
U2OS/NOD2
% MYC positive cells
40
30
20
10
0
MYC-SPATA2:
-
WT
Y338A

Figure S7:, related to Figure 7

(A) Relative levels of *CXCL8*, *TNF*, *A20*, and *NFKBIA* transcripts measured by qRT-PCR on cDNA from WT, CYLD KO, and SPATA2 KO U2OS/NOD2 cells treated with TNF (5ng/ml for 3 h). (B) WT, CYLD KO, and SPATA2 KO U2OS/NOD2 cell lysates from experiment shown in **Figure 7C** were analyzed by western blot analysis as indicated. (C) Intracellular flow cytometry analysis of Myc-positive cells in SPATA2 KO U2OS/NOD2 cells transfected with Myc-SPATA2 variants as indicated. Cells were treated with Brefeldin A (5 μg/ml) and Monensin (2 μM) for 5 h. Data represents mean ± SEM of three independent experiments.

Extended Experimental Procedures

Sequence analysis
Multiple alignment analysis shown in **Figure 5A** was performed with ClustalX on SPATA2 sequences (NCBI accession): Hs (Homo sapiens); Q9UM82, Pt (Pan troglodytes); H2QKJ9, Mam (Macaca mulatta); F6YDM2, Mm (Mus musculus); Q8K004, Rn (Rattus norvegicus); Q91XS7, Clf (Canis lupus familiaris); F1PY42, Bt (Bos taurus); E1BLR1, Eq (Equus caballus); F7CMQ6, Ss (Sus scrofa); A0A0B8RVG9, Xt (Xenopus tropicalis); F7EKQ2, Gg (Gallus gallus); Q5ZI97.

Plasmids and cloning
The following plasmids used in the study have been described previously; pcDNA3-HOIP-V5/His and pcDNA3-HOIL-1-V5/His (Haas et al., 2009). pcDNA3-HOIP PUB+NZF-V5/His and pcDNA3-HOIP N102D-V5/His, pCMV2-FLAG-CYLD, pCMV2-FLAG-CYLD(C601A), pGEX-6P-1-Ubiquillin-UBAx, and the NF-κB luciferase reporter plasmids pBIIX-Luc and TK-renilla-Luc (Hrdinka et al., 2016). CYLD ΔB-box was generated by overlap extension and splicing PCR using pCMV2-FLAG-CYLD as template. pcDNA3.1 Myc-SPATA was generated by PCR amplification from cDNA and insertion into pcDNA3.1 vector. All SPATA2 variants were created by PCR-based site-directed mutagenesis using pcDNA3.1 MYC-SPATA2 as template.
For biochemical and structural studies the coding sequence of full-length SPATA2 was amplified from a brain cDNA library (Clontech) using KOD HotStart DNA polymerase and the following forward and reverse primers: AAGTTCTGTTTCAGGGCCCGATGGGGAAGCCCAGTTCAATG and ATGGTCTAGAAAGCTTTATCTGTACACGAGATGGGAGAG respectively. The PCR product was cloned into pOPINK, which encodes a 3C cleavable N-terminal His6-GST-tag (Berrow et al., 2007) using Infusion HD cloning (Clontech) and was verified by DNA sequencing.

Antibodies and immunoprecipitation reagents
The following antibodies and reagents were used according to the manufacturers' instructions: mouse monoclonal anti-β-Actin (Chemicon Millipore, Billerica, MA), rat monoclonal anti-cIAP1 (Enzo Life Sciences, Exeter, UK), rabbit polyclonal anti-CYLD (Cell Signaling Technology, Danvers, MA), mouse monoclonal anti-CYLD (Santa Cruz Biotechnology, Santa Cruz, CA; used for immunoblotting in **Figure 1G**; **Figure S1E**), monoclonal mouse-anti-FLAG (Sigma-Aldrich, Gillingham, UK), rabbit polyclonal anti-HOIL-1/RBCK1 (Novus Biologicals, Littleton, CO), rabbit polyclonal anti-HOIP/RNF31 (Sigma-Aldrich), rabbit polyclonal anti-HOIP/RNF31 (Ubiquigent, Dundee, UK), rabbit polyclonal anti-IκBα, (Cell Signaling Technology), control rabbit IgG (Santa Cruz Biotechnology), rabbit polyclonal anti-OTULIN (Abcam, Cambridge, UK), rabbit monoclonal anti-RIPK1 (Cell Signaling Technology), rabbit polyclonal anti-RIPK2 (Santa Cruz Biotechnology), rabbit-polyclonal anti-SHARPIN (Proteintech, Rosemont, IL), rabbit polyclonal anti-SPATA2 (Bethyl Laboratories, Montgomery, TX), mouse monoclonal anti-SPATA2 (Santa Cruz Biotechnology used for immunoblotting in **Figure 1G** and **1H**) monoclonal rabbit anti-TNFR1 (Cell Signaling Technology)**,** mouse monoclonal anti-Tubulin (Clone DM1A, Sigma Aldrich), mouse monoclonal anti-Ubiquitin (Imgenex, San Diego, CA), mouse monoclonal anti-Ubiquitin (clone P4D1, Cell Signaling Technology), mouse monoclonal anti-V5 (AbD Serotec, Kidlington, UK)**.** HRP-conjugated secondary antibodies were from Bio-Rad, Hercules, CA (anti-rabbit), Dako, Glostrup, DK (anti-mouse), and R&D systems, Minneapolis, MN (anti-sheep). For immunoprecipitation, anti-HA-agarose conjugate (Clone HA-7, Sigma Aldrich), anti-FLAG affinity gel (Clone M2, Sigma Aldrich) and Protein A/G plus agarose (Santa Cruz Biotechnology) was used. For intracellular staining of IL-8 for flow cytometry the APC conjugated mouse anti-human IL-8 Antibody (clone E8N1, BioLegend, SanDiego, CA) was used.

Cell lines
NOD2-expressing U2OS-Flp-In™ T-REx™ (U2OS/NOD2) cells were cultured in DMEM GlutaMax (Gibco Life Technologies, Carlsbad, CA) supplemented with 10% (v/v) FBS (Sigma-Aldrich) and 1% (v/v) Penicillin-Streptomycin (Gibco Life Technologies) and transfected using Fugene 6 (Promega). Throughout the study, the U2OS/NOD2 cells were cultured and stimulated in the absence of doxycycline unless otherwise indicated. HCT116 cells were cultured in McCoy's 5A (modified) GlutaMax (Gibco®), supplemented with 10% FBS and 1% Penicillin-Streptomycin (Gibco®).

Purification of endogenous ubiquitin conjugates
Ubiquitin conjugates were purified from U2OS/NOD2 cells treated with 200 ng/ml L18-MDP (Invivogen, San Diego, CA) and/or 1 μM Compound A (a kind gift from Tetralogic Therapeutics) using GST-1xUBA^{ubq} ubiquitin affinity reagent (TUBE) (Fiil et al., 2013). Briefly, cells were lysed in TUBE lysis buffer (20 mM Na_2HPO_4, 20 mM NaH_2PO_4, 1% (v/v) NP-40, 2 mM EDTA) supplemented with 1 mM DTT (Sigma Aldrich), 5 mM N-Ethylmaleimide (NEM; Sigma Aldrich), cOmplete protease inhibitors (Roche Diagnostics, Burgess Hill,

UK), and PhosSTOP (Roche Diagnostics). TUBE (50 μg/ml) was added pre-bound to Glutathione Sepharose 4B beads (GE Healthcare) for at least 1 hr. Lysates were cleared by centrifugation, mixed with beads and incubated agitating at 4°C for a minimum of 2 hr. The beads were washed four times in 500 μl of ice-cold PBS 0.1% (v/v) Tween-20. The bound material was eluted with 1x lysis sample buffer.

Intracellular flow cytometry of IL-8

U2OS/NOD2 cells were stimulated with 200 ng/ml L18-MDP for 5 h in the presence of 5 μg/ml Brefeldin A (BioLegend, San Diego, CA) and 2 μM Monensin (BioLegend,) protein transport inhibitors. After stimulation, cells were washed with PBS, dissociated by Trypsin/EDTA solution (Gibco Life Technologies), and collected by centrifugation. Cells were fixed with IC Fixation Buffer (BioLegend) O/N at 4°C, washed with PBS, permeabilized using Perm/Wash Buffer containing 2% (v/v) FCS, 0.1% (w/v) saponin and 0.1% (w/v) NaN_3 in PBS and incubated in the Perm/Wash Buffer with anti-IL8/APC (100x) and/or anti-Myc/DyLight 488 (500x, MA1-21316-D488, Thermo-Fisher Scientific, Rockford, IL) for 1 hr at room temperature. The cells were washed in Perm/wash buffer and analyzed by FACS Canto Flow Cytometer (BD Biosciences, San Jose, CA) and data processed using FlowJo software (TreeStar, Ashland, OR).

Immunoprecipitation

Immunoprecipitation of FLAG-CYLD, HOIP-V5, Myc-SPATA2 was from U2OS/NOD2 cells. Cells were transfected and treated as indicated. Cells were lysed in IP buffer (TBS with 0.5% NP40) supplemented with 5 mM NEM, 1 mM DTT, cOmplete protease inhibitor cocktail (Roche) and PhosSTOP (Roche) for 30 min on ice. Lysates were cleared by centrifugation and incubated at 4°C overnight with anti-FLAG affinity gel, or anti-V5-coupled beads. Beads were washed five times in 500 μL of ice-cold IP buffer and bound material eluted with 2x LDS. DTT was added to 1 mM and heated for 10 mins at 70 °C. For immunoprecipitation of endogenous CYLD and SPATA2 proteins from U2OS/NOD2 cells, confluent 10 cm plates were lysed in IP lysis buffer or TUBE lysis buffer supplemented with 5 mM NEM, cOmplete protease inhibitor cocktail and PhosSTOP and incubated 1-2 h with anti-CYLD (D1A10, Cell Signaling), anti-SPATA2 (A302-494A, Bethyl), or control rabbit IgG and then overnight with Protein A/G Sepharose. The immunoprecipitated material was washed with lysis buffer and eluted from the beads with Laemmli sample buffer. For Immunoprecipitation of endogenous SHARPIN, cells were lysed in buffer containing 25 mM HEPES (pH 7.4), 150 mM KCl, 2 mM $MgCl_2$, 1 mM EGTA, 0.5% Triton X-100, 5 mM NEM, 1 mM DTT, cOmplete protease inhibitor cocktail and PhosSTOP for 30 min on ice. After sedimentation of the cell debris, 5% of the supernatant was taken as input control. The remaining supernatant was incubated with anti-SHARPIN antibody (rabbit IgG served as negative control) and Protein A/G plus agarose for 4-6 hours at 4°C. After washing in lysis buffer, proteins were eluted from the resin by heating in 1.5x Laemmli sample buffer. For immunoprecipitation of the TNFR1 complex, 2 x 15cm plates of U2OS/NOD2 cells were treated with FLAG-TNF (Enzo Life Sciences, 100 ng/ml) for given time points and cells lysed in TNF lysis buffer (30 mM Tris HCl (pH 7.4), 120 mM NaCl, 2 mM EDTA, 2 mM KCl, 1% Triton X-100, cOmplete protease inhibitor cocktail and PhosSTOP) (Haas et al, 2009). Anti-FLAG affinity gel was added and incubated for a minimum of 2h at 4°C. Beads were washed five times in 500 μl of ice-cold IP buffer and bound material eluted with FLAG peptide (Sigma-Aldrich) at 100 μg/ml. Lysates were analyzed by immunoblotting.

Mass spectrometry analysis of CYLD interactome

Proteomic Sample Preparation. CYLD knock-out U2OS/NOD2 cells were transfected with FLAG-CYLD WT, FLAG-CYLD ΔBBOX, or control empty vector using Fugene 6. After 24 h cells were lysed in 0.5% NP-40/TBS lysis buffer and CYLD was immunoprecipitated using anti-FLAG Sepharose for 4 h at 4C. The immunoprecipitated material was eluted using FLAG peptide (Sigma Aldric) and was further prepared as previously described (Fischer et al., 2012). In brief, protein samples were reduced and alkylated in solution and subjected to chloroform/methanol precipitation. The precipitate was resuspended in 6 M urea in 100 mM Tris at a pH of 7.4, digested with elastase at 37 °C overnight, and desalted using C18 Sep Pak column cartridges (Waters, Elstree, UK).

Sample analysis. After drying down in vacuum, samples were resuspended in buffer A (98% H_2O, 2% acetonitrile, 0.1% formic acid) and analyzed by nano–liquid chromatography tandem mass spectrometry (nano–LC-MS/MS) in technical duplicates as described previously (Fischer and Kessler, 2015). In brief, samples were separated using a nanoUPLC (Easy spray C18 column with a 75 μm × 500 mm, 2.1 μm particle size; Thermo Fisher Scientific, Bremen, Germany) coupled to a Q Exactive tandem mass spectrometer (Thermo Fisher Scientific). MS data was acquired with a resolution of 70,000 at m/z 200 and selecting the Top 15 precursor ions. Ion target in MS1 was $3x10^6$ and $5x10^5$ in MS2 mode. Ions were accumulated for up to 100 ms in MS1 and 128 ms in MS2.

Proteomic Data Analysis. For label free quantitation, raw data was imported into Progenesis QI (Waters) Data was aligned and features detected using default settings. MS2 spectra of the combined data were converted to

.mgf files using the 200 most abundant peaks per MS2 spectrum, followed by identification with Mascot v2.5 (10 ppm precursor and 0.05 Da fragment mass tolerance, UniProt Swiss-Prot human database (retrieved 08/12/2013), 1% FDR, peptide score threshold of 20). Carbamidomethylation (C) was selected as fixed and deamidation (N, Q) and Oxidation (M) were selected as variable modifications. Quantitative data was re-normalized to all significantly identified peptides meeting the above mentioned selection criteria. For each replicate, the enrichment of proteins was calculated relative to the abundance in the IP control. The average of enrichment from each replicate is plotted in the figure using the ggplot2 package (www.ggplot2.org) and the R software environment (www.R-project.org).

RNA isolation, cDNA synthesis and qRT-PCR

Total RNA was isolated using an RNeasy Mini Kit (Qiagen) and DNase digestion was performed on-column with the RNase-Free DNase Set (Qiagen) according to the manufacturer's protocol. Total RNA was reverse transcribed with RevertAid™ Reverse Transcriptase (Thermo-Fisher Scientific) and mixture of anchored oligo$(dT)_{20}$ primers and random pentadecamers in the presence of RiboLock (Thermo-Fisher Scientific). qPCR was performed using SYBR Select Master Mix (Applied Biosystems). cDNA was amplified with the following primer pairs:

Hypoxanthine phosphoribosyltransferase (*HPRT*; used as reference for normalization):
5'-AGCCAGACTTTGTTGGATTTG-3' and 5'-TTTACTGGCGATGTCAATAGG-3',
SPATA2: 5'- TCGCTCAGCTCCTCTAGC-3' and 5'- AGGGCCCGTGTAGGTCTT-3', *TNF*: 5' - TGCTGCAGGACTTGAGAAGA-3' and 5' -GAGGAAGGCCTAAGGTCCAC-3',
CXCL8: 5'-TCTGGCAACCCTAGTCTGCT-3' and 5' -AAACCAAGGCACAGTGGAAC -3, *A20*: 5' - ATGCACCGATACACACTGGA-3' and 5' -GGATGATCTCCCGAAACTGA-3', *NFKBIA*: 5' - GCTGATGTCAATGCTCAGGA-3' and 5' -CCCCACACTTCAACAGGAGT-3'.

Transient RNAi knock-down

U2OS/NOD2 cells were reverse transfected with siRNA oligonucleotides (final concentration 35 nM siRNA oligo) using Lipofectamine RNAiMAX (Invitrogen Life Technologies) according to the manufacturer's instructions. For checking protein levels following *SPATA2* siRNA knockdown (**Figure S1C**), an additional forward transfection of the same siRNA was performed 12 hr following reverse transfection (indicated time points are from reverse transfection). The following siRNA oligonucleotides (Sigma-Aldrich) were used for RNAi-mediated knockdown:

CYLD: SASI_Hs01_00012965, GAAGAAUAUGUUUAGAUAU[dT][dT]
HOIP: GGCGUGGUGUCAAGUUUAA[dT][dT]
SPATA2(SP2-A): SASI_Hs01_00108531, CACCUUCACUCCAAAUCCA[dT][dT]
SPATA2(SP2-B): SASI_Hs01_00108532, CGAGUGUGAGCAGAUGCUA[dT][dT]
SPATA2(SP2-C): SASI_Hs01_00108533, GUGACCAAGCCCUCGAGGU[dT][dT]
Mismatched control (siMM): Mission siNEG Ctrl 1 (Cat. #SIC001)

Luciferase reporter assays

WT, CYLD KO and SPATA2 KO U2OS/NOD2 cells were co-transfected with the NF-κB luciferase reporter construct pBIIX-luc and a thymidine kinase-renilla luciferase construct for normalization of transfection efficiency. Cells were co-transfected with additional plasmids as indicated and assays were performed as previously described (Damgaard et al., 2012). Individual experiments were performed in duplicate.

Sucrose gradient sedimentation

Whole-cell lysate was prepared in IP buffer. Continuous 10-40% (w/v) sucrose gradients in a physiological salt solution (150 mM NaCl, 50 mM Tris pH 7.4, 5 mM EDTA, 1 mM PMSF) were generated using a Gradient Master™ 108 (Biocomp, Fredericton, Canada) and 500 µg of protein lysate was subjected to velocity sedimentation at 36,000 rpm (Optima™ L-100 XP (SW41 rotor), Beckman-Coulter), 4 degrees, for 16 hours. 13 fractions were collected and protein was precipitated with trichloracetic acid at a concentration of 8.4%, incubated on ice for 60 min, and centrifuged at 14,000g for 5 min. The resulting pellet was washed 2x with acetone and resuspended in 2x Laemmli buffer.

To approximate the hydrodynamic radii of individual protein complexes, a mixture of purified, native gel filtration standards (Gel Filtration Markers Kit, MWGF1000, Sigma) was subjected to sucrose gradient sedimentation. Fractions were collected and processed as described above. The sedimentation pattern of each protein standard was determined by SDS-PAGE and subsequent silver staining (Silver Stain for Mass Spectrometry, Pierce).

For densitometry analysis, the intensity of each fraction, minus background signal, was quantified in ImageJ. The resulting values were normalised and plotted relative to the highest intensity value in each blot. Data shown representative of three biological replicates. Data represents mean ± SEM.

Generation of knockout cells by CRISPR/Cas9

To generate knockout U2OS/NOD2 cells, cells were transfected using Fugene HD with CRISPR/Cas9 KO Plasmid (Santa Cruz Biotechnology) containing gRNA, Cas9 and EGFP marker. For generation of SPATA2 KO, sc-406210 was used; for CYLD KO, sc-400882 was used.

After 36 h top 10 % GFP-positive cells were sorted by Flow Cytometry and cloned by limiting dilution to obtain single cell clones. Individual clones were validated by western blotting with SPATA2 or CYLD specific antibodies and, for SPATA2 KO clones, co-immunoprecipitation with endogenous CYLD (**Figure 1H; Figure S1E**). The generation of HCT116 HOIP KO cells was previously described (Hrdinka et al., 2016).

Cell transfection

U2OS/NOD2 cells were transiently transfected using Fugene 6 transfection reagent (Promega, Madison, WI) according to manufacturer's instructions.

Statistical analysis

Statistical analysis was performed using Prism 5 or 6 (GraphPad Software Inc). Two-way ANOVA was used to determine statistical significance, except in **Figure 1C** where the two-tailed Student's test was used.

Protein expression and purification

His6-GST-SPATA2 PUB domain constructs were expressed in Rosetta2 (DE3) pLacI cells. Cells were grown at 30 °C in 2xTY medium supplemented with 30 μg/ml kanamycin and 34 μg/ml chloramphenicol to an OD600 of 0.6-1.0. The culture was cooled to 16 °C prior to overnight induction with 400 μM IPTG. Cells were resuspended and lysed by sonication in lysis buffer (20 mM Tris pH 7.4, 300 mM NaCl, 50 mM imidazole, 2 mM β-mercaptoethanol, 10 % (v/v) glycerol, lysozyme, DNaseI (Sigma), 1 mM PMSF and protease inhibitor cocktail (Roche). SPATA2 was purified by immobilised metal affinity chromatography using a HisTrap column (GE Life Sciences). Pooled fractions were dialysed overnight into cation exchange chromatography buffer (20 mM MES pH 6.0, 50 mM NaCl, 4 mM DTT and 10% (v/v) glycerol). For biophysical and structural analysis the His6-GST-tag was cleaved by overnight incubation with 3C protease during dialysis. GST-SPATA2 and SPATA2 PUB domain constructs were purified by cation exchange chromatography (sulphopropyl (SP), GE Life Sciences). Eluted fractions were subjected to size exclusion chromatography (HiLoad 16/60 Superdex 75, GE Life Sciences) in buffer containing 20 mM Tris pH 7.4, 600 mM NaCl, 4 mM DTT. The resultant fractions were judged to be 95-99% pure following SDS-PAGE analysis and flash frozen. HOIP PUB domain was expressed and purified according to (Elliott et al., 2014). Owing to proteolysis of SPATA2 C-terminal of the PUB domain within *E. coli*, the SPATA2 PUB-PIM sequence (7-356) was coexpressed with the HOIP PUB domain that had been sub-cloned into the pETDuet vector (Novagen). GST-SPATA2 (7-356) and His6-HOIP (1-184) were purified using HisTrap affinity and then anion exchange chromatography in buffer containing 20 mM Tris pH 8.5, 10 % (v/v/) glycerol and 4 mM DTT, which enable the removal of excess HOIP before finally being purified by size exclusion chromatography (HiLoad 16/60 Superdex 75, GE Life Sciences). CYLD USP was expressed in sf9 cells and purified according to (Komander et al., 2008) with the exception that 10 %(v/v) glycerol was included in the anion exchange buffer (20 mM Tris pH 8.5, 4 mM DTT, 10 % (v/v) glycerol).

GST pull-down assays

30 μg GST, GST-HOIP PUB (1-184) and GST-SPATA2 PUB (1-241) variants were bound to 25 μl glutathione resin (packed volume) (GE Life Sciences) and incubated for 1 hr at 4 °C in 450 μl buffer (20 mM Tris pH 7.4, 600 mM NaCl, 4 mM DTT). Resin were washed in 3 x 450 μl buffer and incubated with 30 μg CYLD USP variants (in slight molar excess) in 200 μl buffer for 1 hr at 4 °C. Resin were washed in 4 x 450 μl to remove non-bound protein before the resin were resuspended in 50 μl SDS loading buffer prior to SDS-PAGE analysis.

Analytical size exclusion chromatography binding studies

Binding studies with purified HOIP, SPATA2 and CYLD variants were performed on an AKTA Micro system (GS Life Sciences) using a Superdex 75 PC 3.2/30 column equilibrated in: 20 mM Tris pH 7.4, 400 mM NaCl, 2 mM DTT. For studies involving HOIP and SPATA2 PUB domains, 50 μM of each PUB domain was mixed with 50 μM CYLD at room temperature for 10 minutes. Fractions containing protein were mixed with SDS loading buffer prior to SDS-PAGE analysis.

Multi-angle light scattering

The mass of SPATA2 and CYLD complexes in solution was determined by size exclusion multi-angle light scattering (SEC-MALS) using either a Superdex 75 10/300 or Superdex 200 10/300 (GE Life Sciences) connected to a Wyatt Heleos II 18 angle light scattering instrument coupled to a Wyatt Optilab rEX online refractive index detector. Protein samples (100 μl of 2 mg/ml, with the exception of the CYLD B-box 3 mg/ml

and SPATA2 CYLD complex (50 μM) 3.3 mg/ml) were loaded at 0.5 ml/min in buffer containing 20 mM Tris pH 7.4, 200 mM NaCl, 2 mM DTT. A BSA run at 2 mg/ml was used to determine inter-detector delay and peak broadening effects. The protein concentration was determined from the excess differential refractive index based on 0.186 RI per 1 g/ml. The concentration and observed scattering intensities were used to calculate the absolute molecular mass using the ASTRA6 software (Wyatt technology). Partial dissociation of each complex results in a lower observed molecular weight, and the complex fraction can be calculated by dividing the observed molecular weight by the expected/calculated molecular weight. An additional 10% error can be assumed from SEC-MALS measurements.
We estimate the stoichiometry of the trimeric HOIP–SPATA2–CYLD complex as 2:2:2 due to the lack of unbound protein in the SEC MALS run that was performed with equimolar amounts of protein. The lower observed molecular weight (170 kDa as compared to 208 kDa) likely originates from partial complex dissociation, which is already observed in individual binary complexes (see complex fraction), and is likely more pronounced in the trimeric complex.

Crystallization, data collection and refinement
Conventional methods to obtain crystals of the SPATA2 PUB domain failed to yield any crystals, during screening of different SPATA2 PUB domain constructs and crystallisation conditions. Serendipitously, crystals of the SPATA2 PUB domain were obtained from a construct (7-219) that had been purified by size exclusion chromatography (HiLoad 16/60 Superdex 75, GE Life Sciences) in buffer containing 20 mM MES pH 6.0, 200 mM NaCl, 4 mM DTT. The resultant fractions could not be concentrated beyond ~2 mg/ml however, after incubation at 4 °C over a period of a week crystals were observed in the concentrator. Crystals were transferred to a solution containing 20 mM MES pH 6.0, 200 mM NaCl, 4 mM DTT and 30 % (v/v) glycerol prior to cryo-cooling. Crystals of the HOIP PUB domain (5-180) in complex with SPATA2 PIM (334-344) were grown by sitting-drop vapour with 1.5 molar excess of SPATA2 PIM peptide. HOIP SPATA2 PIM was mixed with reservoir containing 1.7-1.9 M $(NH_4)_2SO_4$, 50 mM sodium cacodylate pH 6.4-7.0, 15 mM MgCl2 in a 1:2 ratio. Crystals were transferred into a solution containing 2M lithium sulphate prior to cryo-cooling.

Diffraction data were collected at Diamond Light source beamline I02. Diffraction images were processed using xia2 (Winter, 2010) and manually scaled using AIMLESS (Evans and Murshudov, 2013). Owing to the low sequence homology between SPATA2 and the HOIP/PNGase PUB domains (25% sequence identity) and slight deviation of core helices relative to one another, conventional molecular replacement was not sufficient for determining the SPATA2 PUB structure. The *ab initio* phase determining programme AMPLE was used to determine the structure of the SPATA2 PUB domain through placement of short poly-Ala idealised helices (Bibby et al., 2012). Manual inspection of the search models and additional molecular replacement of idealised helices using PHASER (McCoy et al., 2007) resulted in suitable phases that allowed automatic building of the resultant electron density map using ARP/wARP (Langer et al., 2008). The structure of the HOIP-SPATA2 PIM peptide complex was determined by molecular replacement using PHASER (McCoy et al., 2007) and the HOIP PUB domain (PDB ID 4OYK) with the OTULIN PIM peptide deleted. Iterative rounds of model building and refinement were performed with COOT (Emsley et al., 2010) and PHENIX (Adams et al., 2011) respectively. In the case of the HOIP PUB SPATA2 PIM complex, unambiguous electron density could be built into for the SPATA2 PIM peptide after initial rounds of refinement. Data collection and refinement statistics can be found in **Table 1**. All structure figures were generated with Pymol (www.pymol.org).

DUB assays
Qualitative gel-based DUB assays were performed as in (Komander et al., 2009). Briefly, CYLD USP was diluted in 25 mM Tris pH 7.4, 200 mM NaCl and 5 mM DTT and activated at 21 °C in the absence or presence of SPATA2 PUB for 15 min. 10 μM tetraUb or 4 μM diUb was incubated with indicated amounts of CYLD in 50 mM Tris pH 7.4, 100 mM NaCl, 4 mM DTT at 25 °C. Time points were taken and mixed with SDS sample buffer to stop the reaction. Samples were resolved on 4-12% SDS-PAGE gradient gels and visualised by silver staining (Biorad).

Fluorescence polarisation binding assays
Serial dilutions of either HOIP/SPATA2 PUB domain variants into FP assay buffer: 20 mM Tris pH 7.4, 300 mM NaCl, 2 mM DTT were prepared and 10 μl of this was aliqutoed into a 384-well low volume plate (Corning) containing 10 μl of 100 nM FITC-Ahx PIM peptides of either OTULIN (49-67), p97 (797-806) or SPATA2 (334-344). Fluorescence polarisation was recorded on a PheraStar plate reader (BMG Labtech) using an optics module with λex = 485 nm and λem = 520 nm. Polarisation values were plotted against PUB concentration and fitted to a one-site binding model using Graphpad Prism 5.

Fluorescence polarisation cleavage assays

Change in fluorescence polarisation upon cleavage of the peptide/isopeptide bond between the distal and proximal ubiquitin moieties of Met1/Lys63 diUb were used to derive Michaelis-Menten rates. Reactions were performed in a black 384-well low volume plate (Corning) and measured on a PheraStar plate reader (BMG Labtech) using an optics module with λex = 485 nm and λem = 520 nm. Assays were performed based upon (Keusekotten et al., 2013). Briefly, Met1/Lys63 diUb were serially diluted into FlAsH buffer (20 mM Tris pH 7.4, 200 mM NaCl, 2 mM β-mercaptoethanol, 0.04 mg/ml BSA) and contained a fixed concentration of 300 nM FlAsH-tagged Met1/Lys63 diUb. To each well, 10 µl of substrate was mixed with 10 µl of CYLD USP (583-956) and the change in fluorescence polarisation was recorded at 25 °C. Observed fluorescence polarisation values were converted to percentage of substrate cleavage by comparing to baseline values of FlAsH-tagged monoUb. All measurements were corrected by subtracting changes in fluorescence polarisation of FlAsH-tagged Met1/Lys63 diUb alone and all measurements were performed in triplicate. Initial rates of substrate cleavage were calculated using GraphPad Prism 5. Initial rates were plotted against substrate concentration at a fixed CYLD concentration. The K_M of CYLD USP for Met1- and Lys63-diUb is greater than the highest substrate concentrations used in the assay, preventing direct determination of Michaelis-Menten parameters. Therefore, the DUB activity (k_{cat}/K_M) was calculated from a linear fit of substrate concentration plotted against initial rate and corrected against amount of CYLD USP used in the assay. To enable efficient initial rates, 3 µM CYLD USP +/- 10 µM SPATA2 PUB was used for Lys63 diUb cleavage and 0.4 µM CYLD USP +/- 4 µM SPATA2 PUB was used for Met1 diUb cleavage.

Nuclear Magnetic Resonance Spectroscopy
His6-SUMO-tagged CYLD B-box (778-855) was expressed in 2M9 medium supplemented with ^{15}N NH_4Cl and purified using standard HisTrap and anion exchange protocols described above. BEST-TROSY spectra (Solyom et al., 2013) were acquired at 298K on Bruker Avance2+ 700 MHz spectrometer equipped with a cryogenic triple TCl probe. Data processing and analysis were performed with Topspin3.0 (Bruker) and Sparky (http://www.cgl.ucsf.edu/home/sparky/).

Analytical Ultracentrifugation
Equilibrium sedimentation experiments for a complex of SPATA2 PUB and CYLD USP were performed on an Optima XL-I analytical ultracentrifuge (Beckmann) using An50Ti rotors. Sample volumes of 110 µl with protein concentrations of 1 µM in 20 mM HEPES, pH 7.5, 200 mM NaCl, 4 mM DTT were loaded in 12 mm 6-sector cells and centrifuged at 5700, 6900, and 12000 rpm until equilibrium was reached at 20 ˚C. At each speed, comparison of several scans was used to judge whether or not equilibrium had been reached. Data were processed and analysed using UltraSpin software (http://www.mrc-lmb.cam.ac.uk/dbv/ultraspin2/) and SEDPHAT (Schuck, 2003). The partial-specific volumes (v-bar), solvent density and viscosity were calculated using SEDNTERP (Dr. Thomas Laue, University of New Hampshire.

Surface Plasmon Resonance
SPR was performed using a Biacore T200 using CM5-sensor chips (GE Healthcare). Both reference control and analyte channels were equilibrated 20 mM HEPES, pH 7.5, 200 mM NaCl, 4 mM DTT. SPATA2 PUB was immobilised onto the chip surface *via* amide coupling using the supplied kit (GE Healthcare) to reach an RU value of between 100 and 300 for separate experiments. SPR runs were performed with analytes injected for 120 s followed by a 600 s dissociation in a 1:2 dilution series with initial concentrations of: 2 µM for CYLD USP WT and I790D; 55.2 µM for CYLD L622D; 63 µM for CYLD ΔB-Box; and 484 µM for CYLD B-box (778-855). After reference and buffer signal correction, sensorgram data were fitted using KaleidaGraph (Synergy Software) and Prism (Graphpad Prism).
The kinetic rate constants of dissociation were measured by fitting dissociation data at time t (R_{dissoc}) using a single-exponential function:

$$R_{dissoc} = R_o \exp^{-(k_{off} t)} + RI + Dt \qquad (1)$$

where k_{off} is the dissociation rate constant, R_o is maximum change in resonance, RI is the bulk resonance change and D is a linear drift term. The rate constants of association were obtained by fitting the observed change in resonance signal (R_{assoc}) at time t using the following equation:

$$R_{assoc} = \left(\frac{k_{on} C R_{\max}}{k_{on} C + k_{off}} \right) \left[1 - \exp^{-(k_{on} C + k_{off}) t} \right] + RI + Dt \qquad (2)$$

where k_{on} is the association rate constant, C is the analyte concentration and $R_{\max}$ is the maximum change in resonance. The affinity for the interactions were calculated from the ratios of the microscopic rate constants:

$$K_d = \frac{k_{off}}{k_{on}} \tag{3}$$

For CYLD USP WT and I709D, the equilibrium response (R_{eq}) data were fitted using a single site interaction model to determine K_d:

$$R_{eq} = \left(\frac{CR_{max}}{C + K_d}\right) + N_s C + B \tag{4}$$

where C is the analyte concentration and R_{max} is the maximum response at saturation, N_s is a linear non-specific binding term and B is the background resonance. Data were transformed to fraction bound with the fitted values using:

$$f_b = \left(\frac{R - B}{R_{max} + N_s C}\right) \tag{5}$$

For L622D and ΔBox, as saturation was not reached, data were transformed using parameters fitted for WT.

Supplemental references

Adams, P.D., Afonine, P.V., Bunkoczi, G., Chen, V.B., Echols, N., Headd, J.J., Hung, L.W., Jain, S., Kapral, G.J., Grosse Kunstleve, R.W., *et al.* (2011). The Phenix software for automated determination of macromolecular structures. Methods *55*, 94-106.

Berrow, N.S., Alderton, D., Sainsbury, S., Nettleship, J., Assenberg, R., Rahman, N., Stuart, D.I., and Owens, R.J. (2007). A versatile ligation-independent cloning method suitable for high-throughput expression screening applications. Nucleic Acids Res *35*, e45.

Bibby, J., Keegan, R.M., Mayans, O., Winn, M.D., and Rigden, D.J. (2012). AMPLE: a cluster-and-truncate approach to solve the crystal structures of small proteins using rapidly computed ab initio models. Acta Crystallogr D Biol Crystallogr *68*, 1622-1631.

Elliott, P.R., Nielsen, S.V., Marco-Casanova, P., Fiil, B.K., Keusekotten, K., Mailand, N., Freund, S.M., Gyrd-Hansen, M., and Komander, D. (2014). Molecular Basis and Regulation of OTULIN-LUBAC Interaction. Mol Cell *54*, 335-348.

Emsley, P., Lohkamp, B., Scott, W.G., and Cowtan, K. (2010). Features and development of Coot. Acta Crystallogr D Biol Crystallogr *66*, 486-501.

Evans, P.R., and Murshudov, G.N. (2013). How good are my data and what is the resolution? Acta Crystallogr D Biol Crystallogr *69*, 1204-1214.

Fiil, B.K., Damgaard, R.B., Wagner, S.A., Keusekotten, K., Fritsch, M., Bekker-Jensen, S., Mailand, N., Choudhary, C., Komander, D., and Gyrd-Hansen, M. (2013). OTULIN restricts Met1-linked ubiquitination to control innate immune signaling. Mol Cell *50*, 818-830.

Fischer, R., and Kessler, B.M. (2015). Gel-aided sample preparation (GASP)--a simplified method for gel-assisted proteomic sample generation from protein extracts and intact cells. Proteomics *15*, 1224-1229.

Fischer, R., Trudgian, D.C., Wright, C., Thomas, G., Bradbury, L.A., Brown, M.A., Bowness, P., and Kessler, B.M. (2012). Discovery of candidate serum proteomic and metabolomic biomarkers in ankylosing spondylitis. Mol Cell Proteomics *11*, M111 013904.

Haas, T.L., Emmerich, C.H., Gerlach, B., Schmukle, A.C., Cordier, S.M., Rieser, E., Feltham, R., Vince, J., Warnken, U., Wenger, T., *et al.* (2009). Recruitment of the linear ubiquitin chain assembly complex stabilizes the TNF-R1 signaling complex and is required for TNF-mediated gene induction. Mol Cell *36*, 831-844.

Hrdinka, M., Fiil, B.K., Zucca, M., Leske, D., Bagola, K., Yabal, M., Elliott, P.R., Damgaard, R.B., Komander, D., Jost, P.J., *et al.* (2016). CYLD Limits Lys63- and Met1-Linked Ubiquitin at Receptor Complexes to Regulate Innate Immune Signaling. Cell Rep *14*, 2846-2858.

Keusekotten, K., Elliott, P.R., Glockner, L., Fiil, B.K., Damgaard, R.B., Kulathu, Y., Wauer, T., Hospenthal, M.K., Gyrd-Hansen, M., Krappmann, D., *et al.* (2013). OTULIN antagonizes LUBAC signaling by specifically hydrolyzing Met1-linked polyubiquitin. Cell *153*, 1312-1326.

Komander, D., Lord, C.J., Scheel, H., Swift, S., Hofmann, K., Ashworth, A., and Barford, D. (2008). The structure of the CYLD USP domain explains its specificity for Lys63-linked polyubiquitin and reveals a B box module. Mol Cell *29*, 451-464.

Komander, D., Reyes-Turcu, F., Licchesi, J.D., Odenwaelder, P., Wilkinson, K.D., and Barford, D. (2009). Molecular discrimination of structurally equivalent Lys 63-linked and linear polyubiquitin chains. EMBO Rep *10*, 466-473.

Langer, G., Cohen, S.X., Lamzin, V.S., and Perrakis, A. (2008). Automated macromolecular model building for X-ray crystallography using ARP/wARP version 7. Nature protocols *3*, 1171-1179.

McCoy, A.J., Grosse-Kunstleve, R.W., Adams, P.D., Winn, M.D., Storoni, L.C., and Read, R.J. (2007). Phaser crystallographic software. J Appl Crystallogr *40*, 658-674.

Solyom, Z., Schwarten, M., Geist, L., Konrat, R., Willbold, D., and Brutscher, B. (2013). BEST-TROSY experiments for time-efficient sequential resonance assignment of large disordered proteins. J Biomol NMR *55*, 311-321.

Winter, G. (2010). xia2: an expert system for macromolecular crystallography data reduction. Journal of Applied Crystallography *43*, 186-190.
