# Supplementary material for: SPATA2 Links CYLD to LUBAC, Activates CYLD, and Controls LUBAC Signaling
Source: Mol Cell. 2016 Sep 15;63(6):990–1005. doi: 10.1016/j.molcel.2016.08.001 (PMC5031558; doi:10.1016/j.molcel.2016.08.001)
Supplement: Document S2. Article plus Supplemental Information [file mmc3.pdf]

Molecular Cell

Article

SPATA2 Links CYLD to LUBAC, Activates CYLD, and Controls LUBAC Signaling

Graphical Abstract

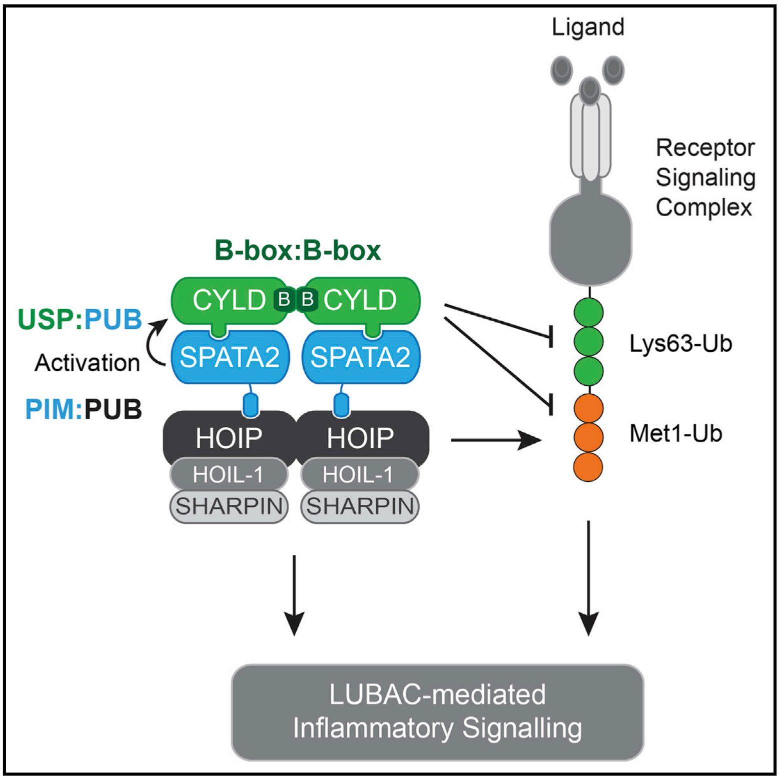

Authors

Paul R. Elliott, Derek Leske, Matous Hrdinka, ..., Stefan M.V. Freund, David Komander, Mads Gyrd-Hansen

Correspondence

dk@mrc-lmb.cam.ac.uk (D.K.), mads.gyrd-hansen@ludwig.ox.ac.uk (M.G.-H.)

In Brief

Elliott et al. show that SPATA2 bridges CYLD with LUBAC to regulate substrate ubiquitination and inflammatory signaling. Structural and biochemical work defines SPATA2-CYLD and SPATA2-HOIP interfaces, reveals SPATA2-mediated CYLD activation, and provides first insights into stoichiometry of LUBAC complexes.

Highlights

- CYLD recruitment to LUBAC and the TNF receptor 1 complex is mediated by SPATA2
- SPATA2 forms a high-affinity complex with CYLD and stimulates CYLD's activity
- SPATA2, like OTULIN, uses a conserved PIM to dock to the HOIP PUB domain
- SPATA2 limits ubiquitination of LUBAC substrates to regulate inflammatory signaling

Accession Numbers

5LJM
5LJN

Elliott et al., 2016, Molecular Cell *63*, 990–1005
September 15, 2016 © 2016 The Authors. Published by Elsevier Inc.
http://dx.doi.org/10.1016/j.molcel.2016.08.001

CellPress

Molecular Cell
Article

SPATA2 Links CYLD to LUBAC, Activates CYLD, and Controls LUBAC Signaling

Paul R. Elliott,[1,4] Derek Leske,[2,4] Matous Hrdinka,[2] Katrin Bagola,[2] Berthe K. Fiil,[2] Stephen H. McLaughlin,[1] Jane Wagstaff,[1] Norbert Volkmar,[2] John C. Christianson,[2] Benedikt M. Kessler,[3] Stefan M.V. Freund,[1] David Komander,[1,*] and Mads Gyrd-Hansen[2,5,*]
[1]Division of Protein and Nucleic Acid Chemistry, MRC Laboratory of Molecular Biology, Francis Crick Avenue, Cambridge CB2 0QH, UK
[2]Ludwig Institute for Cancer Research, Nuffield Department of Medicine, University of Oxford, Old Road Campus Research Building, Oxford OX3 7DQ, UK
[3]TDI Mass Spectrometry Laboratory, Target Discovery Institute, Nuffield Department of Medicine, University of Oxford, Roosevelt Drive, Oxford OX3 7FZ, UK
[4]Co-first author
[5]Lead Contact
*Correspondence: dk@mrc-lmb.cam.ac.uk (D.K.), mads.gyrd-hansen@ludwig.ox.ac.uk (M.G.-H.)
http://dx.doi.org/10.1016/j.molcel.2016.08.001

SUMMARY

The linear ubiquitin chain assembly complex (LUBAC) regulates immune signaling, and its function is regulated by the deubiquitinases OTULIN and CYLD, which associate with the catalytic subunit HOIP. However, the mechanism through which CYLD interacts with HOIP is unclear. We here show that CYLD interacts with HOIP via spermatogenesis-associated protein 2 (SPATA2). SPATA2 interacts with CYLD through its non-canonical PUB domain, which binds the catalytic CYLD USP domain in a CYLD B-box-dependent manner. Significantly, SPATA2 binding activates CYLD-mediated hydrolysis of ubiquitin chains. SPATA2 also harbors a conserved PUB-interacting motif that selectively docks into the HOIP PUB domain. In cells, SPATA2 is recruited to the TNF receptor 1 signaling complex and is required for CYLD recruitment. Loss of SPATA2 increases ubiquitination of LUBAC substrates and results in enhanced NOD2 signaling. Our data reveal SPATA2 as a high-affinity binding partner of CYLD and HOIP, and a regulatory component of LUBAC-mediated NF-κB signaling.

INTRODUCTION

Modification of proteins with ubiquitin (Ub) constitutes a versatile posttranslational modification that regulates a variety of cellular processes, including receptor signaling, cell cycle progression, and DNA damage responses. Ub signaling controls activation of nuclear factor-κB (NF-κB) and innate immune responses downstream of pattern recognition receptors (PRRs) such as Toll-like receptors (TLRs), nucleotide-oligomerization domain (NOD)-like receptors, and cytokine receptors, such as tumor necrosis factor (TNF) receptor 1 (TNFR1) (Fiil and Gyrd-Hansen, 2014; Jiang and Chen, 2011).

Stimulation of these receptors triggers assembly of multi-protein signaling complexes where Ub ligases and deubiquitinases (DUBs) coordinate the deposition of Ub chains linked via lysine 63 (Lys63-Ub) and methionine 1 (Met1-Ub) on protein substrates to orchestrate activation of the TAB-TAK1 and NEMO-IKKα/β kinase complexes, respectively. Activation of IKK is required for productive signaling and NF-κB-mediated transcriptional responses, and its activation depends on the binding of Met1-Ub by the IKK subunit NEMO (also known as IKKγ) (Fiil and Gyrd-Hansen, 2014; Jiang and Chen, 2011).

Met1-Ub is conjugated by the linear ubiquitin chain assembly complex (LUBAC), composed of HOIP, HOIL-1, and SHARPIN, which has emerged as an important Ub ligase activity in innate immune signaling and immune regulation (Boisson et al., 2012, 2015; Damgaard et al., 2012; Gerlach et al., 2011; Ikeda et al., 2011; Kirisako et al., 2006; Tokunaga et al., 2011). In cells, LUBAC function is regulated by at least two associated DUBs, OTULIN and CYLD, which serve both overlapping and unique roles. OTULIN exclusively hydrolyzes Met1-Ub, prevents spurious accumulation of Met1-Ub on LUBAC components under basal conditions, and restricts ubiquitination of LUBAC substrates such as RIPK2 after NOD2 stimulation (Fiil et al., 2013; Keusekotten et al., 2013). CYLD, a bona fide tumor suppressor and negative regulator of NF-κB signaling (Harhaj and Dixit, 2012), disassembles both Met1-Ub and Lys63-Ub (Komander et al., 2009; Ritorto et al., 2014; Sato et al., 2015). CYLD is recruited with LUBAC to TNFR1 and NOD2 signaling complexes and trims Ub chains on LUBAC substrates (Draber et al., 2015; Hrdinka et al., 2016; Takiuchi et al., 2014).

Both CYLD and OTULIN associate with LUBAC via an N-terminal peptide:N-glycanase/UBA- or UBX-containing protein (PUB) domain in the catalytic subunit HOIP (Draber et al., 2015; Elliott et al., 2014; Hrdinka et al., 2016; Schaeffer et al., 2014; Takiuchi et al., 2014). OTULIN harbors a PUB-interacting motif (PIM) that inserts into a PIM binding pocket in the HOIP PUB domain to create a high-affinity interaction important for its ability to counteract LUBAC auto-ubiquitination (Elliott et al., 2014; Schaeffer et al., 2014). The association of CYLD with LUBAC and its recruitment to receptor complexes also involves the PIM binding

 © 2016 The Authors. Published by Elsevier Inc.
This is an open access article under the CC BY license (http://creativecommons.org/licenses/by/4.0/).

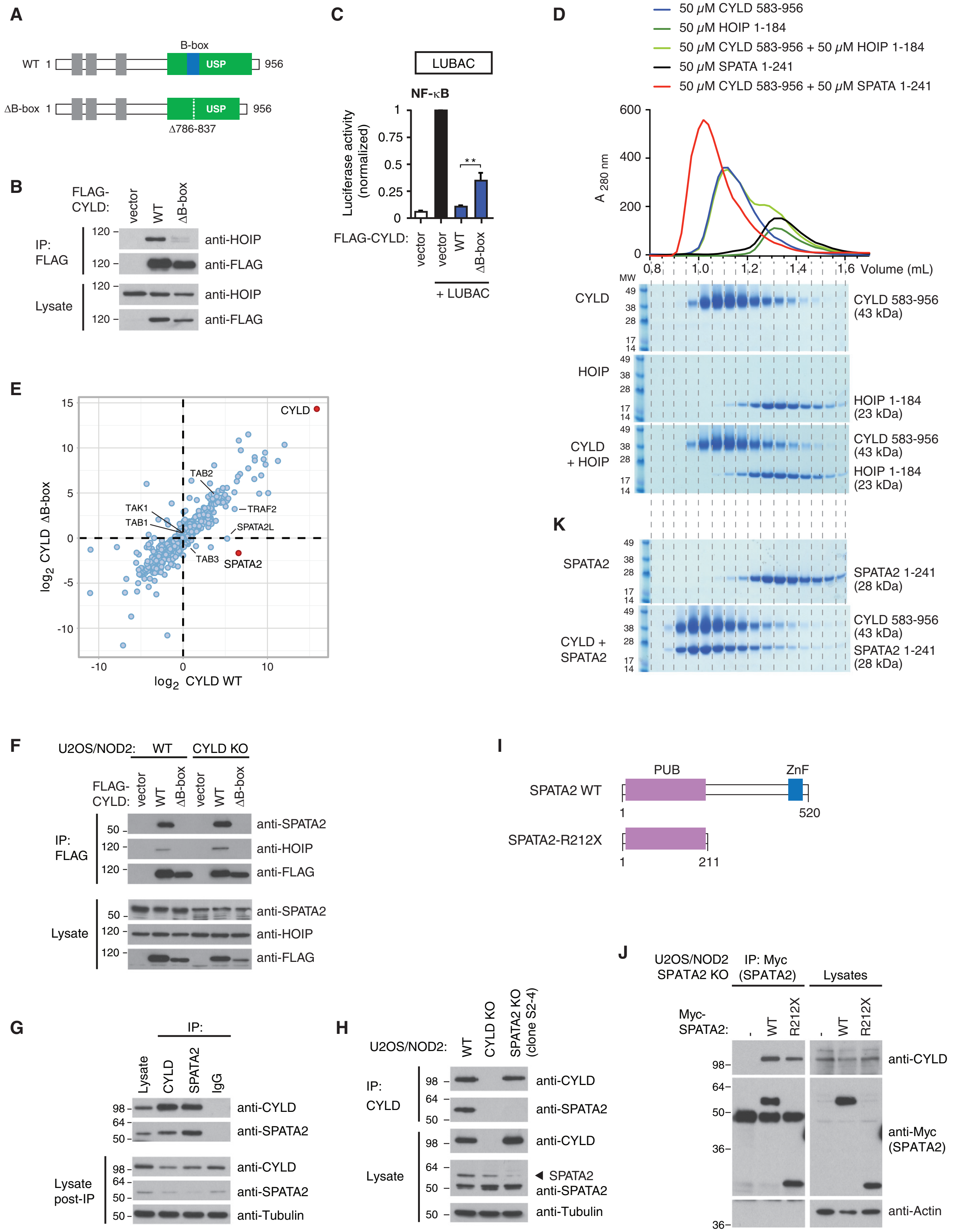

(legend on next page)

pocket in the HOIP PUB domain (Draber et al., 2015; Hrdinka et al., 2016; Takiuchi et al., 2014), but the molecular basis for the interaction is not understood.

Here, we show that CYLD does not interact directly with HOIP and identify the uncharacterized protein spermatogenesis-associated protein 2 (SPATA2) as the factor that bridges CYLD and HOIP. SPATA2 contains a PIM that binds the PUB domain in HOIP, but not other PUB domains. SPATA2 binds the USP domain of CYLD via its PUB domain, but in a PIM-independent manner. Interestingly, this interaction also activates CYLD. Functionally, SPATA2 mediates the recruitment of CYLD to the TNFR1 signaling complex and supports CYLD-dependent regulation of LUBAC-mediated NF-κB signaling.

RESULTS

SPATA2 Binds CYLD in a B-box-Dependent Manner

In cells, CYLD interaction with HOIP depends on the PIM-binding pocket within the HOIP PUB domain (Draber et al., 2015; Hrdinka et al., 2016; Takiuchi et al., 2014). Mutational analysis of CYLD showed that the interaction is mediated by the CYLD USP domain and depends on the CYLD B-box (Takiuchi et al., 2014) (Figures 1A and 1B). Deletion of the CYLD B-box impaired the ability of CYLD to antagonize LUBAC-mediated NF-κB activity, suggesting that this region in CYLD regulates LUBAC function (Figures 1C and S1A, available online). However, CYLD does not contain a discernible PIM within this region and there was no obvious binding between the CYLD USP and HOIP PUB domain, as determined by size-exclusion chromatography (SEC), where CYLD and HOIP eluted in separate fractions (Figure 1D), in vitro pull-downs (Figure S1B), or nuclear magnetic resonance (NMR) spectroscopy (data not shown) using purified proteins. This prompted us to search for a protein that would mediate the interaction between CYLD and HOIP. For this, we purified FLAG-tagged wild-type (WT) CYLD and CYLD with deletion of the B-box (ΔB-box) from CYLD knockout (KO) U2OS/NOD2 cells (Figure 1B) and subjected the purified material to liquid chromatography-tandem mass spectrometry (LC-MS/MS). Among the detected proteins were previously described CYLD interactors such as TAK1 (Reiley et al., 2007), TNF receptor-associated factor 2 (TRAF2) (Kovalenko et al., 2003), and also the known CYLD interactors SPATA2 and SPATA2-like (SPATA2L) (Sowa et al., 2009). Strikingly, SPATA2 was the most highly enriched protein in the CYLD WT sample relative to the CYLD ΔB-box sample, indicating that the interaction depends on the CYLD B-box (Figure 1E; Table S1). Also, SPATA2L was preferentially enriched by CYLD WT whereas TAK1-TAB components and TRAF2 were co-purified similarly with CYLD WT and CYLD ΔB-box (Figure 1E; Table S1). The interaction between SPATA2 and CYLD was confirmed in cells by co-immunoprecipitation of ectopic or endogenous proteins (Figures 1F and 1G). To ensure that SPATA2 was detected by the SPATA2 antibody in the CYLD immunoprecipitation, the specificity of the antibody was carefully characterized in cells where SPATA2 was depleted by RNAi-mediated silencing and in cells where SPATA2 had been genetically deleted by CRISPR/Cas9 genome editing (Figures 1H and S1C–S1E). This confirmed that the antibody detected SPATA2 in cell lysates and in CYLD immunoprecipitation experiments, but it also showed that the antibody detected several unrelated proteins in lysates, some of which migrated at a similar molecular weight (MW) as SPATA2 (Figures 1H and S1C–S1F). We then analyzed which region of SPATA2 was responsible for CYLD binding. This showed that the SPATA2 N-terminal PUB domain mediates CYLD interaction in cells (Figures 1I and 1J). Indeed, the CYLD USP domain (aa 583–956) and SPATA2 PUB domain (aa 1–241) formed an SEC-stable complex (Figure 1K), confirming a direct interaction.

Characterization of the CYLD-SPATA2 Interaction

It was striking that while CYLD was unable to form a stable complex with the PUB domain of HOIP, it instead interacted with the PUB domain in SPATA2 (Figures 1D, 1K, and S1B). A crystal structure of the SPATA2 PUB domain (aa 7–219) at 1.45 Å resolution (Figures 2A–2D, S2A, and S2B; Table 1) revealed a fold most similar to that of the extended PUB domain in HOIP (root-mean-square deviation [RMSD] 2.4 Å) (Elliott et al., 2014) (Figures 2A–2C) and the smaller PUB domain of PNGase (RMSD 3.2 Å) (Zhao et al., 2007) (Figure 2D). Species conservation of SPATA2 mapped onto the surface of the PUB domain reveals that while the PIM pocket is highly conserved (Figures 2E, 2F, S2A, and S2C), this interaction site is also very different from canonical PUB domains. The previously mapped PUB-PIM

Figure 1. Identification of SPATA2 as a B-box-Dependent CYLD Interactor

(A) Schematic representation of full-length and ΔB-box (deletion of aa residues 786–837) CYLD.

(B) Immunoprecipitation and western blot analysis of transiently expressed FLAG-CYLD variants from CYLD KO U2OS/NOD2 cells.

(C) NF-κB activity in U2OS/NOD2 cell lysates transfected with dual luciferase reporters and co-expressed with vector, LUBAC (HOIL-1/HOIP), and/or FLAG-CYLD variants as indicated. Luciferase activity is shown relative to the activity of LUBAC in transfected cells. Data represent the mean ± SEM of six independent experiments, each performed in duplicate. **$p < 0.01$.

(D) Analytical SEC profile of CYLD USP (583–956) (blue), HOIP PUB domain (1–184) (green), and HOIP and CYLD at equimolar ratio (light green). Coomassie-stained SDS-PAGE gels below show protein-containing fractions. Also included are SPATA2 PUB domain (1–241) (black) and CYLD and SPATA2 at equimolar ratio (red) (see K).

(E) Mass spectrometry analysis of FLAG-CYLD interactomes purified with anti-FLAG from CYLD KO U2OS cell lysates. The scatterplot shows enrichment of proteins co-purified with CYLD WT (x axis) and CYLD ΔB-box (y axis). Dots below the diagonal indicate B-box-dependent interactors.

(F) Immunoprecipitation and western blot analysis of transiently expressed FLAG-CYLD variants from WT or CYLD KO U2OS/NOD2 cells.

(G) Immunoprecipitation and western blot analysis of endogenous CYLD and SPATA2 from U2OS/NOD2 cells. Control IgG served as negative control.

(H) Immunoprecipitation and western blot analysis of transiently expressed FLAG-CYLD from WT, CYLD KO, or SPATA2 KO (clone S2-4) U2OS/NOD2 cells.

(I) Schematic representation of SPATA2 WT and SPATA2 R212X (SPATA2 aa 1–211).

(J) Immunoprecipitation and western blot analysis of transiently expressed Myc-SPATA2 variants from SPATA2 KO U2OS/NOD2 cells.

(K) Coomassie-stained SDS-PAGE gels (linked to profile in D) of SPATA2 PUB and CYLD USP-SPATA2 PUB complex.

Also see Figure S1.

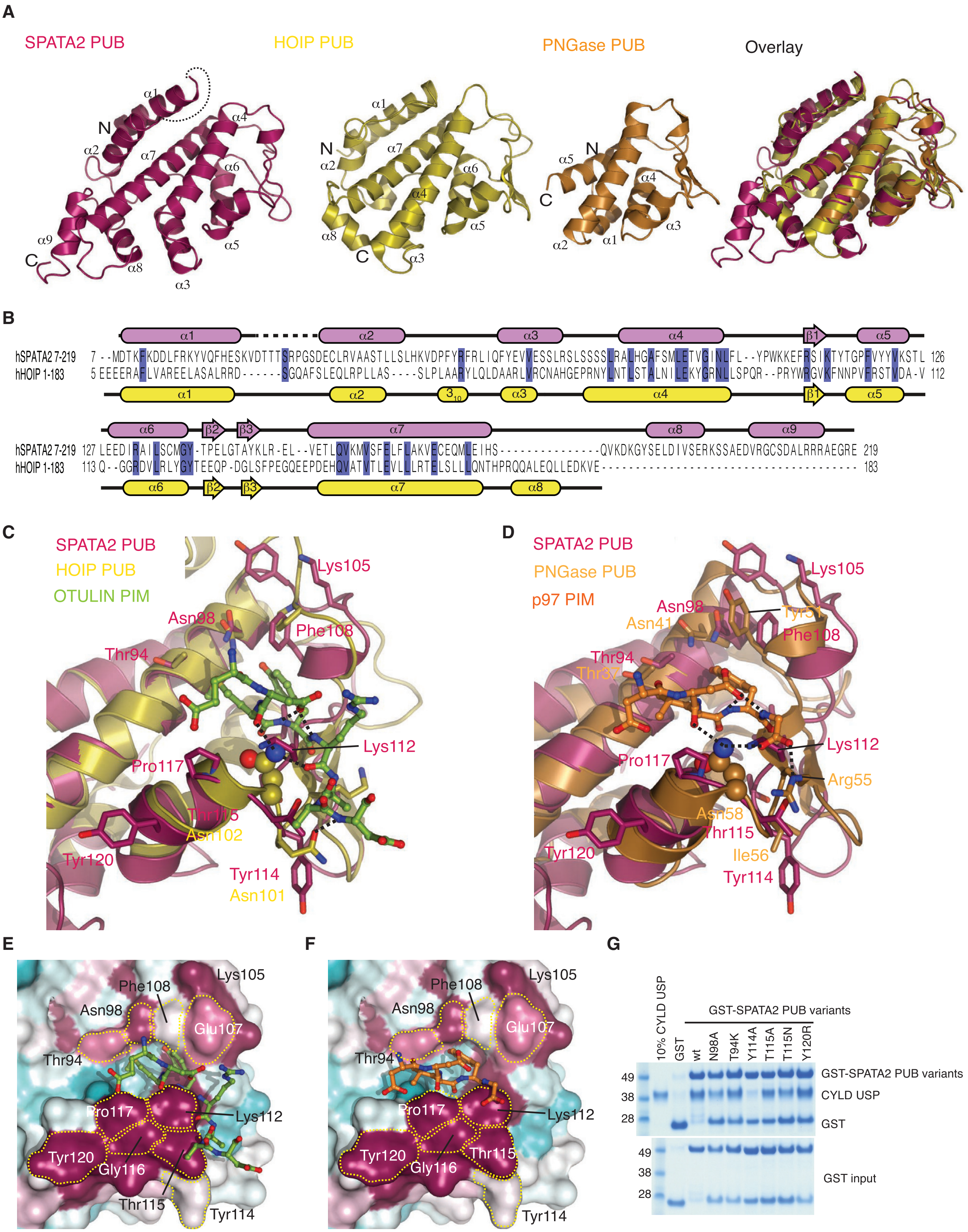

(legend on next page)

Table 1. Data Collection and Refinement Statistics

	SPATA2 7–219	HOIP 5–180 + SPATA2 334–344
Data Collection		
Beamline	Diamond I02	Diamond I02
Space group	$P\ 2_1$	$P\ 4_3$
a, *b*, *c* (Å)	43.48, 51.14, 56.29	89.04, 89.04, 53.56
α, β, γ (°)	90.00, 105.97, 90.00	90.00, 90.00, 90.00
Wavelength	0.9794	0.9795
Resolution (Å)	54.12–1.45 (1.48–1.45)	62.96–2.70 (2.83–2.70)
R_{merge}	3.7 (32.7)	7.2 (67.9)
$< I / \sigma I >$	12.1 (2.2)	9.5 (2.0)
CC(1/2)	0.99 (0.91)	0.99 (0.61)
Completeness (%)	90.2 (81.2)	97.7 (99.7)
Redundancy	3.0 (2.9)	2.7 (2.7)
Refinement		
Resolution (Å)	54.11–1.45	62.96–2.70
No. reflections	37,458	11,425
R_{work} / R_{free}	18.2/22.5	23.3/28.1
No. Atoms		
Protein	1,722	2,695
Ligand/ion	12	15
Water	308	–
B Factors		
Wilson B	15.73	57.1
Protein	25.1	61.3
Ligand/ion	21.8	85.0
Water	38.0	–
RMSDs		
Bond lengths (Å)	0.005	0.002
Bond angles (°)	0.772	0.526
Ramachandran statistics (outliers, allowed, favored)	0.0, 1.4, 98.6	0.0, 2.6, 97.4

Related to Figures 2 and 5. Values in parentheses are for the highest-resolution shell. Datasets were collected and structures determined from a single crystal.

interactions include a conserved Asp-Leu/Met-Tyr (see below), in which Leu and Tyr occupy a deep, hydrophobic gorge on the PUB surface, the PIM pocket. In SPATA2, this pocket is significantly different from both HOIP as well as PNGase structures, and modeling of interactions with PIM peptides derived from OTULIN or p97 would generate steric clashes (Figures 2E and 2F). Consistently, the SPATA2 PUB domain does not bind PIM peptides (see below), suggesting an interaction motif in CYLD may need to display distinct properties. Nonetheless, the high conservation in this area did suggest that this surface may mediate CYLD interactions, and single amino acid mutations in or near the SPATA2 PIM pocket interfered with CYLD binding (Figure 2G). In particular, mutations in the "lower wall" of the SPATA2 PIM pocket (Y114A, T115N, and T115A) decreased CYLD interactions, while mutation of residues in the "upper wall" of the pocket (N98A and T94K) did not have strong effects on CYLD binding. The strongest effect on CYLD binding was observed when we mutated Tyr114, which points away from the PIM pocket (Figures 2E and 2G), supporting that CYLD binds the SPATA2 PUB domain in a PIM-independent manner.

The CYLD B-box Mediates CYLD Dimerization and Is Essential for SPATA2 Complex Formation

The B-box dependence of the CYLD-SPATA2 interaction (Figures 1B, 1C, and 1F) could suggest a direct interaction between the B-box and the SPATA2 PUB domain. Surprisingly, NMR titration experiments with an isolated ^{15}N-labeled B-box domain (aa 778–855) and unlabeled SPATA2 PUB domain revealed no signs of an interaction (Figure S3A). This contrasts the formation of a stable complex on gel filtration between the SPATA PUB domain and the CYLD USP domain (Figure 1K).

Further studies using SEC coupled to multi-angle light scattering (SEC-MALS) revealed that the intact CYLD USP domain (aa 583–956, including the B-box, 43 kDa) eluted as a dimer (86 kDa), while CYLD ΔB-box (35 kDa) eluted as a monomer (34 kDa) (Figures 3A and S3B). Furthermore, the isolated B-box domain (aa 778–855, 9.1 kDa) eluted as a dimer (17.1 kDa) (Figure 3A). Strikingly, the SPATA2 PUB domain (aa 1–241, 27.6 kDa), a monomer on its own (26.6 kDa), formed a 2:2 complex with dimeric CYLD USP domain of 136 kDa (calculated 140 kDa) in SEC-MALS (Figure 3A). This was independently confirmed by equilibrium analytical ultracentrifugation (Figure S3C). Thus, nicely consistent with the earlier results from mass spectrometry (Figure 1E), this complex forms in a B-box-dependent manner (Figures 3A and S3B).

Previous structural analysis of the CYLD USP domain (Komander et al., 2008) suggested how the B-box domain might mediate CYLD dimerization. A conserved B-box surface forms a hydrophobic interface across a crystallographic symmetry axis, which orients the two catalytic domains such that both can access polyUb without steric hindrance (Komander et al., 2008;

Figure 2. Structure of the SPATA2 PUB Domain

(A) Far left, structure of the SPATA2 PUB domain (residues 7–219). Left, structure of the HOIP PUB domain (residues 1–184, PDB: 40YJ). Right, structure of the PNGase PUB domain (residues 12–110, PDB: 2HPJ). Far right, superimposition of all PUB domains on the α4, α7 core helices.

(B) Structure-based sequence alignment of SPATA2 and HOIP PUB domains.

(C) Superposition of SPATA2 onto HOIP PUB bound to OTULIN PIM (PDB: 40YK, PIM in green). Asn102, important for OTULIN PIM binding, is shown in a ball-and-stick model.

(D) Superposition of SPATA2 and PNGase PUB bound to p97 PIM (PDB: 2HPL).

(E and F) Conservation analysis of the SPATA2 PIM pocket (purple, conserved; white/cyan, not conserved). PIM peptides from OTULIN (E) and p97 (F) are modeled, revealing significant clashes.

(G) Pull-down using GST-SPATA2 PUB domain and mutants within the PIM pocket against the CYLD USP domain.

Also see Figure S2.

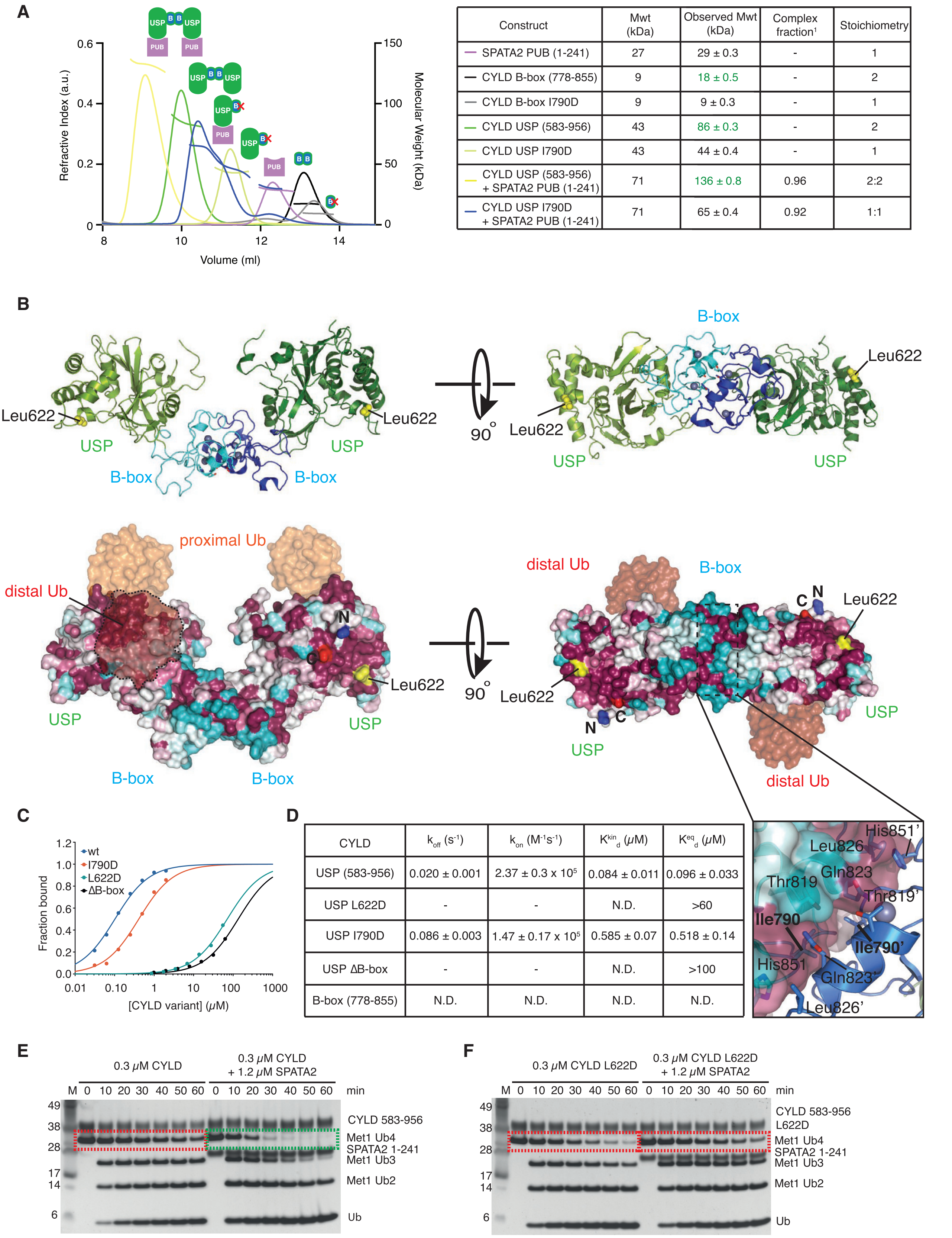

Construct	Mwt (kDa)	Observed Mwt (kDa)	Complex fraction[1]	Stoichiometry
SPATA2 PUB (1-241)	27	29 ± 0.3	-	1
CYLD B-box (778-855)	9	18 ± 0.5	-	2
CYLD B-box I790D	9	9 ± 0.3	-	1
CYLD USP (583-956)	43	86 ± 0.3	-	2
CYLD USP I790D	43	44 ± 0.4	-	1
CYLD USP (583-956) + SPATA2 PUB (1-241)	71	136 ± 0.8	0.96	2:2
CYLD USP I790D + SPATA2 PUB (1-241)	71	65 ± 0.4	0.92	1:1

CYLD	k_{off} (s^{-1})	k_{on} ($M^{-1}s^{-1}$)	K^{kin}_d (μM)	K^{eq}_d (μM)
USP (583-956)	0.020 ± 0.001	2.37 ± 0.3 x 10^5	0.084 ± 0.011	0.096 ± 0.033
USP L622D	-	-	N.D.	>60
USP I790D	0.086 ± 0.003	1.47 ± 0.17 x 10^5	0.585 ± 0.07	0.518 ± 0.14
USP ΔB-box	-	-	N.D.	>100
B-box (778-855)	N.D.	N.D.	N.D.	N.D.

(legend on next page)

Sato et al., 2015) (Figure 3B). Mutation of Ile790 (I790D) within the B-box dimerization interface generated a monomeric B-box (9 kDa) and monomeric CYLD USP domain (44 kDa) (Figure 3A). Interestingly, the I790D dimerization mutant still formed a 1:1 complex with the SPATA2 PUB domain of 65 kDa that was less stable on SEC-MALS (Figure 3A, blue profile). These results were corroborated by in vitro pull-downs and surface plasmon resonance (SPR), which revealed the CYLD-SPATA2 interaction to be high affinity (96 nM), and also showed no binding of the isolated B-box domain to SPATA2 (Figures 3C, 3D, S3D, and S3E). CYLD I790D affinity for SPATA2 was still respectable (518 nM), but a higher k_{off} likely affects stability of the complex when CYLD is not dimeric (Figures 3C, 3D, and S3E).

Surface conservation depicted on the CYLD dimer revealed that while exposed areas of the B-box were not conserved, a highly conserved surface exists on the solvent-exposed side of the CYLD palm domain not involved in Ub interactions (Figure 3B). Mutation of conserved surface residues revealed that Leu622 was essential for SPATA2 interaction (Figure S3D). Leu622 is 45 Å away from the B-box domain, indicating that the catalytic USP core of CYLD mediates SPATA2 binding. Hence, CYLD and SPATA2 form a highly stable heterotetramer in vitro and likely in cells, which is destabilized when the core dimerization domain, the B-box of CYLD, is deleted or disrupted.

SPATA2 Activates CYLD

A number of USP domains are activated allosterically by binding partners (Sahtoe and Sixma, 2015). Recent structural insights into the USP46-UAF1 complex and the USP12-UAF1-WDR20 complex revealed that the activators interact with surfaces remote from the catalytic center and mediate activation via long-range allosteric mechanisms (Li et al., 2016; Yin et al., 2015).

SPATA2 also has a reproducible, yet moderate, activating effect on CYLD. Hydrolysis of Met1- or Lys63-linked tetraUb is enhanced in presence of SPATA2 (Figures 3E and S3F). Quantification of this effect employing fluorescent Met1/Lys63-linked diUb substrates (Keusekotten et al., 2013) reveals an ~2-fold increase in k_{cat}/K_M in presence of SPATA2 (Figures S3G and S3H). Deletion of the B-box does not affect the catalytic activity or structure of the CYLD USP domain (Komander et al., 2008; Sato et al., 2015), but SPATA2-mediated CYLD activation is lost in CYLD ΔB-box (Figure S3I) or in CYLD L622D (Figure 3F), as these CYLD variants no longer bind SPATA2. Likewise, mutations of SPATA2 residues in the CYLD interface decrease or abolish its ability to activate CYLD (Figures S3J–S3L). SPATA2 does not affect CYLD specificity, which remains Lys63 and Met1 specific at the diUb level (Figure S3M), and still does not significantly cleave Lys48-tetraUb (data not shown).

Together, this reveals a first function for SPATA2 in CYLD activation.

SPATA2 Links CYLD to HOIP

We next investigated whether SPATA2 associates with LUBAC and would be involved in mediating CYLD recruitment. Indeed, purification of endogenous LUBAC by SHARPIN immunoprecipitation showed that SPATA2 co-immunoprecipitates with LUBAC and that the interaction is dependent on HOIP (Figure 4A), as is also the case for CYLD and OTULIN (Elliott et al., 2014; Hrdinka et al., 2016; Schaeffer et al., 2014; Takiuchi et al., 2014). Moreover, CYLD did not co-immunoprecipitate with ectopic V5-tagged HOIP or endogenous SHARPIN in SPATA2 KO cells, indicating that SPATA2 is required for the interaction of CYLD with LUBAC (Figures 4B and 4C). Purification of the TNFR1 complex with FLAG-tagged TNF recruits LUBAC (Haas et al., 2009) and, with it, CYLD (Draber et al., 2015). Importantly, the recruitment of CYLD to the TNFR1 complex depended on SPATA2 (Figure 4D).

To determine the amount of cellular CYLD associated with SPATA2 and LUBAC, we performed sucrose gradient sedimentation experiments on lysates from unstimulated WT, CYLD KO, and SPATA2 KO U2OS/NOD2 cells. This revealed that the majority of cellular CYLD sedimented along with HOIP in high MW fractions (with densitometry peak at 600–700 kDa) in WT cells (Figures 4E and S4A). Strikingly, in SPATA2 KO cells CYLD shifted to markedly lower MW fractions (with densitometry peak at ~200 kDa). In accordance with the mass spectrometry

Figure 3. Mapping the SPATA2 Interaction Site on CYLD

(A) SEC-MALS analysis of CYLD and SPATA2 PUB variants using a Superdex 75 size-exclusion column. Right, table listing monomeric and observed MWs for each protein or complex, fraction of complex formed (calculated from difference between expected and observed MW; see Supplemental Experimental Procedures), and the resultant stoichiometry.

(B) Composite structure of human CYLD containing the B-box (PDB: 2VHF) bound to Met1-linked diUb (PDB: 3WXE). The crystal structure of human CYLD contains a crystallographic-related interface between the B-box domain, forming a 2-fold symmetrical axis. Top, cartoon representation of the CYLD dimer with the B-box colored blue and the USP domain in green. Met1-diUb is not shown for clarity. Bottom, identical view as above, but CYLD is shown as a surface colored according to sequence conservation. Met1-diUb is shown as a semi-transparent surface. Leu622 as well as N and C termini are highlighted. Insert, B-box dimerization interface where the surface of one B-box has been removed to highlight the conserved hydrophobic and polar contacts that form across the dimer interface.

(C) Equilibrium response fitting for CYLD variants based on surface plasmon resonance (SPR) data. The fraction of CYLD variant bound to SPATA2 PUB is plotted against varying CYLD concentrations.

(D) Table summarizing SPR data. The k_{off}, k_{on} and the kinetic dissociation constant, $K^{kin}{}_d$ (= k_{off}/k_{on}), are calculated from the response curves in Figure S3E. The equilibrium dissociation constant $K^{eq}{}_d$ is calculated from (C). No binding was observed from the CYLD B-box and only weak binding was observed from CYLD L622D and CYLD ΔB-box, preventing accurate determination of $K^{eq}{}_d$. For comparison, CYLD L622D and CYLD ΔB-box data were fitted assuming the maximum responses were similar to WT. N.D., not detected.

(E) SPATA2 binding to CYLD enhances Ub chain hydrolysis. Hydrolysis of Met1-linked tetraUb by CYLD if followed over time without or with addition of SPATA2. Green boxes highlight the tetraUb band.

(F) DUB assay as in (E) using CYLD L622D mutant, which is unable to bind SPATA2 and does not show enhanced activity.

Also see Figure S3.

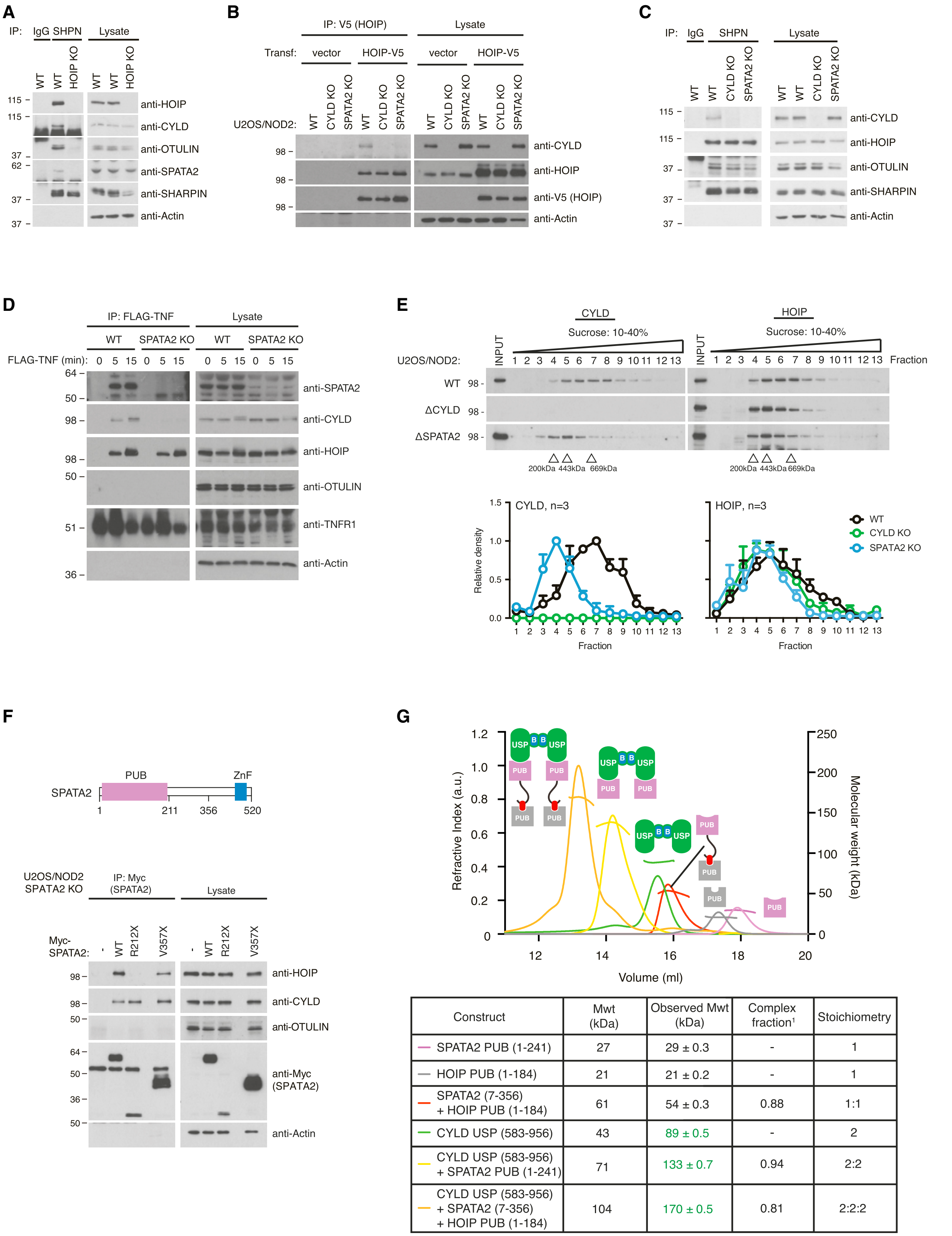

Construct	Mwt (kDa)	Observed Mwt (kDa)	Complex fraction[1]	Stoichiometry
SPATA2 PUB (1-241)	27	29 ± 0.3	-	1
HOIP PUB (1-184)	21	21 ± 0.2	-	1
SPATA2 (7-356) + HOIP PUB (1-184)	61	54 ± 0.3	0.88	1:1
CYLD USP (583-956)	43	89 ± 0.5	-	2
CYLD USP (583-956) + SPATA2 PUB (1-241)	71	133 ± 0.7	0.94	2:2
CYLD USP (583-956) + SPATA2 (7-356) + HOIP PUB (1-184)	104	170 ± 0.5	0.81	2:2:2

(legend on next page)

experiments and the affinity of the CYLD and SPATA2 interaction, this suggests that a substantial fraction of the cellular pool of CYLD is in complex with SPATA2, which links it to LUBAC and possibly other high-order complexes. Although the sedimentation of HOIP was less influenced by the absence of SPATA2 (and CYLD), the highest MW HOIP complexes consistently shifted toward lower MW fractions in the SPATA2 KO and CYLD KO cells as compared with WT cells, which might reflect the loss of SPATA2 and/or CYLD from LUBAC complexes (Figures 4E and S4A).

We next determined how SPATA2 binds to HOIP. Expression of Myc-tagged SPATA2 variants in SPATA2 KO cells showed that while the PUB domain of SPATA2 (aa 1–211) did not bind HOIP, an extended construct of SPATA2 (aa 1–356) co-immunoprecipitated both CYLD and HOIP, indicating that the region following the PUB domain (aa 212–356) mediates the HOIP interaction (Figure 4F). This cellular binding study was confirmed in vitro with purified components. SEC-MALS with purified proteins showed that the interaction between SPATA2 (aa 7–356) and HOIP PUB (aa 1–184) is direct and that they form a 1:1 complex of 54 kDa (calculated 61 kDa) (Figure 4G, gray and red curves). Strikingly, the extended SPATA2 fragment formed a trimeric complex with CYLD and HOIP that eluted with an MW of 170 kDa, indicative of a stable 2:2:2 complex (Figure 4G, red, green, and orange curves; see Supplemental Experimental Procedures for details on stoichiometry calculation). This confirmed that SPATA2 is able to bridge CYLD with LUBAC via HOIP (Figure 4G).

While OTULIN, CYLD, and SPATA2 co-purified with LUBAC (Figure 4A), the association of OTULIN with LUBAC was independent of SPATA2 and CYLD (Figure 4C). Moreover, OTULIN was not co-purified with SPATA2, suggesting that SPATA2 (and CYLD) do not occupy the same HOIP/LUBAC molecules as OTULIN (Figure 4F).

SPATA2 and HOIP Bind via a PIM-PUB Interaction

It had been reported that mutation of the HOIP PUB domain "cornerstone" residue Asn102 in the PIM binding pocket (Elliott et al., 2014) abrogates binding not only to OTULIN but also to CYLD (Draber et al., 2015; Hrdinka et al., 2016; Takiuchi et al., 2014). This prompted us to speculate that SPATA2 might harbor a PIM and interacts with HOIP in a similar manner as does OTULIN. Indeed, species conservation analysis of the HOIP-interacting region in SPATA2 (aa 212–356) revealed a highly conserved putative PIM, Asp-Leu-Tyr-Thr, between amino acid residues 336 and 339, which is similar to the OTULIN PIM (Asp-Met-Tyr-Arg) (Elliott et al., 2014; Schaeffer et al., 2014) (Figures 5A and S2C).

A complex crystal structure of the HOIP PUB domain with a peptide spanning the putative SPATA2 PIM sequence (aa 334–344) at 2.7 Å confirmed binding of the SPATA2 PIM to the HOIP PIM pocket (Figures 5B–5D; Table 1). Two complexes in the asymmetric unit of the HOIP PUB-SPATA2 PIM crystals are highly similar (RMSD 0.7 Å) (Figure S5A), and while they crystallized in a distinct space group as compared to the HOIP PUB-OTULIN PIM crystals ($P4_3$ as compared to $P6_1$; Table 1), the PIM peptides overlay perfectly and occupy the PIM pocket in a virtually identical manner (Figure 5E). The major difference is the presence of Leu337 in SPATA2 instead of Met55 in OTULIN (Figure 5E), and, indeed, the SPATA2 PIM resembles the "original" PIM sequence derived from p97 (Asp-Leu-Tyr-Gly-COO^-) (Elliott et al., 2014; Schaeffer et al., 2014; Zhao et al., 2007). In contrast to p97, both SPATA2 and OTULIN constitute internal PIMs, in which residues extending C terminally make additional contacts. In the SPATA2 PIM, Asp340 forms a backbone hydrogen bond with Asn101 in HOIP, mimicking contacts of the OTULIN PIM (Elliott et al., 2014; Schaeffer et al., 2014) (Figure 5C).

The structurally highly similar binding mode was also confirmed by biophysical binding measurements using fluorescently labeled PIM peptides and analyzing their binding to purified PUB domains by fluorescence polarization (FP) (Figures 5F and S5B–S5E). The SPATA2 or OTULIN PIM peptides displayed binding affinities of ~300 nM with the HOIP PUB domain, but did not interact with PUB domains of PNGase or UBXN6 in this assay. The latter interacted only with the "terminal" PIM sequence found in p97, as reported (Elliott et al., 2014). Importantly, and consistent with our structural analysis described above (Figure 2), the SPATA2 PUB domain did not interact with any of the tested PIM peptides (Figures 5F and S5C).

In line with the in vitro analysis, substitution of the conserved Tyr338 in the SPATA2 PIM to Ala (Y338A) or Phe (Y338F) largely abrogated the interaction of SPATA2 with HOIP in cells, without affecting SPATA2 binding to CYLD (Figure 5G). Moreover, coexpression of V5-tagged HOIP and SPATA2 variants showed that the SPATA2 PIM is needed to co-immunoprecipitate CYLD and HOIP, demonstrating that the CYLD-SPATA2 complex is linked to LUBAC via the SPATA2 PIM-HOIP PUB interaction (Figure 5H).

Figure 4. SPATA2 Binds CYLD and HOIP through Distinct Domains

(A) Immunoprecipitation and western blot analysis of endogenous SHARPIN from WT or HOIP KO HCT116 cells.

(B) Immunoprecipitation and western blot analysis of transiently expressed HOIP-V5 in WT, CYLD KO, and SPATA2 KO U2OS/NOD2 cells.

(C) Immunoprecipitation and western blot analysis of endogenous SHARPIN in WT, CYLD KO, and SPATA2 KO U2OS/NOD2 cells.

(D) Purification and western blot analysis of the TNFR1 complex from WT and SPATA2 KO U2OS/NOD2 cells stimulated with FLAG-TNF (100 ng/mL), as indicated using anti-FLAG agarose.

(E) Sucrose gradient sedimentation and western blot analysis of sedimented fractions and lysates from WT, CYLD KO, and SPATA2 KO U2OS/NOD2 cells. Bottom, densitometry analysis of scanned immunoblots from three independent experiments. Values were normalized to the fraction with the highest density on each blot. Data represent means ± SEM.

(F) Top, schematic representation of SPATA2 showing position for truncation variants R212X (1–211) and V357X (1–356). Bottom, immunoprecipitation and western blot analysis of transiently expressed Myc-SPATA2 variants from SPATA2 KO U2OS/NOD2 cells.

(G) SEC-MALS analysis of indicated proteins and complexes on a Superdex 200 column. Bottom, table listing monomeric and observed MWs for each protein or complex, fraction of complex formed (see Figure 3A), and the resultant stoichiometry.

Also see Figure S4.

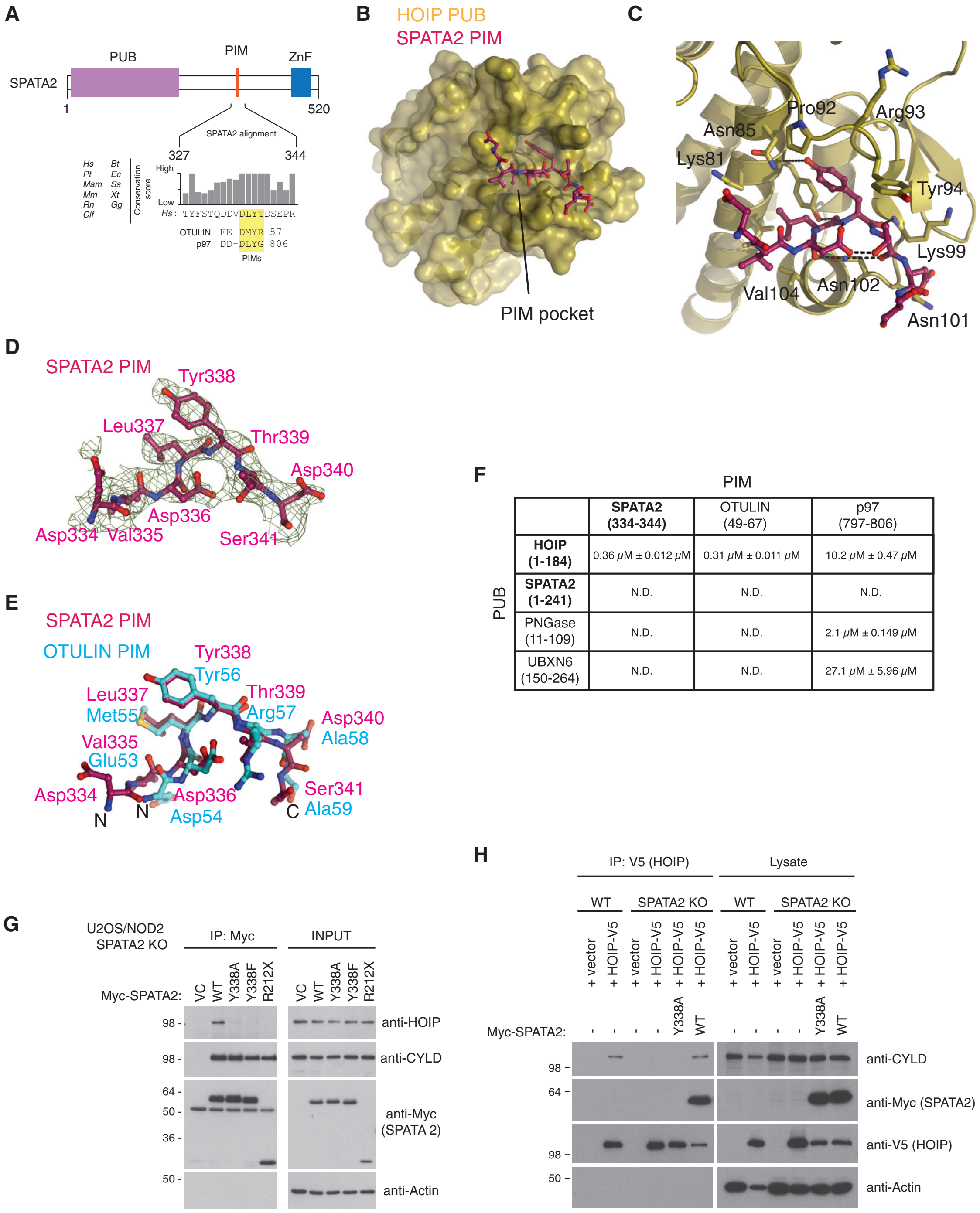

		PIM		
		**SPATA2 (334-344)**	OTULIN (49-67)	p97 (797-806)
PUB	**HOIP (1-184)**	0.36 μM ± 0.012 μM	0.31 μM ± 0.011 μM	10.2 μM ± 0.47 μM
	**SPATA2 (1-241)**	N.D.	N.D.	N.D.
	PNGase (11-109)	N.D.	N.D.	2.1 μM ± 0.149 μM
	UBXN6 (150-264)	N.D.	N.D.	27.1 μM ± 5.96 μM

Figure 5. An HOIP-Specific PIM in SPATA2

(A) Primary sequence alignment of the HOIP PIM in SPATA2. The PIM is highly conserved among SPATA2 orthologs and aligns with the p97 and OTULIN PIMs.

(B) Structure of the HOIP PUB domain (yellow surface) bound to the SPATA2 PIM (purple).

(C) Close-up view of the HOIP PIM pocket with SPATA2 PIM bound. Key residues of the HOIP PUB domain are shown in sticks, and the SPATA2 PIM is shown in ball-and-stick representation.

(D) A simulated annealing composite omit map contoured at 1 σ covering the SPATA2 PIM peptide.

(E) Superposition of the SPATA2 PIM peptide (purple) with the OTULIN PIM (cyan).

(F) Binding affinities of known PUB domains against known PIM peptides calculated by FP binding data using FITC-Ahx-labeled PIM peptides. Experiments were performed in triplicate and errors represent ± SEM. N.D., not detected.

(legend continued on next page)

SPATA2 Links CYLD to Receptor Complexes and NF-κB Signaling

CYLD restricts ubiquitination of LUBAC substrates at receptor complexes to regulate inflammatory signaling (Draber et al., 2015; Hrdinka et al., 2016). We therefore explored the role of SPATA2 in regulating TNFR1 and NOD2 signaling. Strikingly, comparison of RIPK2 and RIPK1 ubiquitination in response to NOD2 stimulation and TNF treatment, respectively, showed that Ub-RIPK2 and Ub-RIPK1 species with a higher apparent MW accumulated in SPATA2 KO cells (three independent clones) and, as expected (Hrdinka et al., 2016), in CYLD KO cells as compared with WT cells (Figure 6A; RIPK2 blot, compare lane 2 with lanes 5, 8, 11, and 14; RIPK1 blot, compare lane 3 with lanes 6, 9, 12, and 15). To further assess the role of SPATA2 in regulating ubiquitination of RIPK2, cells were pre-treated with an Smac-mimetic compound (compound A, CpA), which inhibits the function of inhibitor of apoptosis (IAP) proteins and blocks RIPK2 ubiquitination in WT cells, but not in CYLD-depleted or CYLD KO cells (Damgaard et al., 2013; Hrdinka et al., 2016) (Figure 6B; compare lanes 3 and 6). Accordingly, RIPK2 ubiquitination was retained in SPATA2 KO cells despite CpA treatment, substantiating that SPATA2 regulates RIPK2 ubiquitination after NOD2 stimulation (Figure 6B; compare lane 3 with lanes 9, 12, and 15). Intriguingly, purification of the TNFR1 complex with FLAG-tagged TNF revealed that there was less Ub-RIPK1, Ub-TNFR1, Lys63-Ub, and Met1-Ub retained in the receptor complex in SPATA2 KO cells as compared with WT cells (Figures 6C and S6A). This suggests that SPATA2 not only regulates ubiquitination of LUBAC substrates but also contributes to the retention of ubiquitinated proteins at the TNFR1 complex.

Analysis of productive TNF signaling showed that loss of SPATA2, like CYLD, had little or no effect on activation of the MAP kinase p38, degradation of IκB, or phosphorylation of the NF-κB subunit RelA (Figure 6C) (Hrdinka et al., 2016). Accordingly, the expression of NF-κB response genes was similar in WT, SPATA2 KO, and CYLD KO cells following TNF treatment (Figure S7A). In contrast to this, NOD2 stimulation in SPATA2 KO cells and CYLD KO cells led to a comparable enhanced expression of NF-κB response genes as compared with WT cells, showing that SPATA2, like CYLD, restricts NOD2 signaling (Figure 7A). NOD2 stimulation also led to increased IL-8 production in SPATA2 KO cells as compared with WT cells, albeit the increase in IL-8 was less dramatic than in CYLD KO cells (Figure 7B).

We noted also that baseline NF-κB activity was elevated in both SPATA2 KO and CYLD KO cells as measured by a luciferase-based NF-κB reporter (Figure 7C). To directly address if SPATA2 mediates the ability of CYLD to regulate baseline NF-κB activity, we ectopically expressed CYLD in WT, CYLD KO, and SPATA2 KO cells. As expected, CYLD expression in CYLD KO cells reduced baseline NF-κB activity to the same level as in WT cells (Figure 7C). In contrast, CYLD had no effect on NF-κB activity in the SPATA2 KO cells, even though the expression level of CYLD was similar in all conditions (Figures 7C and S7B). This prompted us to address the function of the SPATA2 PIM in regulating productive NF-κB signaling. For this, IL-8 production was determined in SPATA2 KO cells transiently expressing SPATA2 WT or a PIM mutant (Y338A). Unexpectedly, transient overexpression of SPATA2 in SPATA2 KO cells (Figure S7C) caused spontaneous production of IL-8, which was largely dependent on the SPATA2 PIM (Figure 7D). This suggests that interaction of SPATA2 (and other PIM-containing proteins such as OTULIN) with the HOIP PUB is central to the regulation of LUBAC function and NF-κB signaling.

DISCUSSION

Here, we identify SPATA2 as a new regulatory factor of CYLD and as the protein that bridges CYLD to the Met1-Ub assembly machinery, LUBAC. The recent discovery that CYLD is associated with LUBAC revealed that the Ub-regulating capacity of LUBAC-DUB complexes extends beyond Met1-Ub to include Lys63-Ub and possibly other linkages (Draber et al., 2015; Hrdinka et al., 2016; Takiuchi et al., 2014). It is thus striking that the association of both DUBs with LUBAC is governed by PIMs (in OTULIN and SPATA2) that dock to the HOIP PUB domain in an identical manner. This raises the question if both DUBs can associate with LUBAC simultaneously or if distinct LUBAC-DUB complexes exist. Draber et al. showed that OTULIN and CYLD do not co-purify each other while both interact with LUBAC (Draber et al., 2015). In agreement with this, we reveal that CYLD-SPATA2 and OTULIN interact with distinct LUBAC complexes.

While the composition of LUBAC is currently not clear, at least two copies of HOIP exist in the complex (Elliott et al., 2014; data not shown); also, SHARPIN is dimeric (Stieglitz et al., 2012). We here show that CYLD is a constitutive dimer in solution and that it forms a 2:2 complex with SPATA2. This places two SPATA2 PIMs in close proximity, and the hetero-tetramer binds two copies of HOIP, likely invoking favorable avidity effects. Intriguingly, deletion of SPATA2 did not lead to increased binding of OTULIN to LUBAC, which suggests that the amount of OTULIN available for HOIP binding is limited. In contrast, we had previously observed that OTULIN levels seem to be in excess of HOIP, but only a fraction of it is bound to LUBAC, possibly due to phosphorylation of the PIM (Elliott et al., 2014; Zhao et al., 2007).

Importantly, which DUB is associated has regulatory implications. OTULIN binding to HOIP is required to restrict LUBAC auto-ubiquitination, which is readily detected on HOIP, HOIL-1, and SHARPIN when OTULIN is depleted or mutated in its PIM (Draber et al., 2015; Elliott et al., 2014; Fiil et al., 2013; Hrdinka et al., 2016; Keusekotten et al., 2013). In contrast, CYLD association with LUBAC appears to predominantly regulate LUBAC substrate ubiquitination such as RIPK2 after NOD2 stimulation

(G) Immunoprecipitation and western blot analysis of transiently expressed Myc-SPATA2 variants from SPATA2 KO U2OS/NOD2 cells.
(H) Immunoprecipitation and western blot analysis of transiently expressed HOIP-V5 from WT and SPATA2 KO U2OS/NOD2 cells co-transfected with Myc-SPATA2 variants where indicated.
Also see Figure S5.

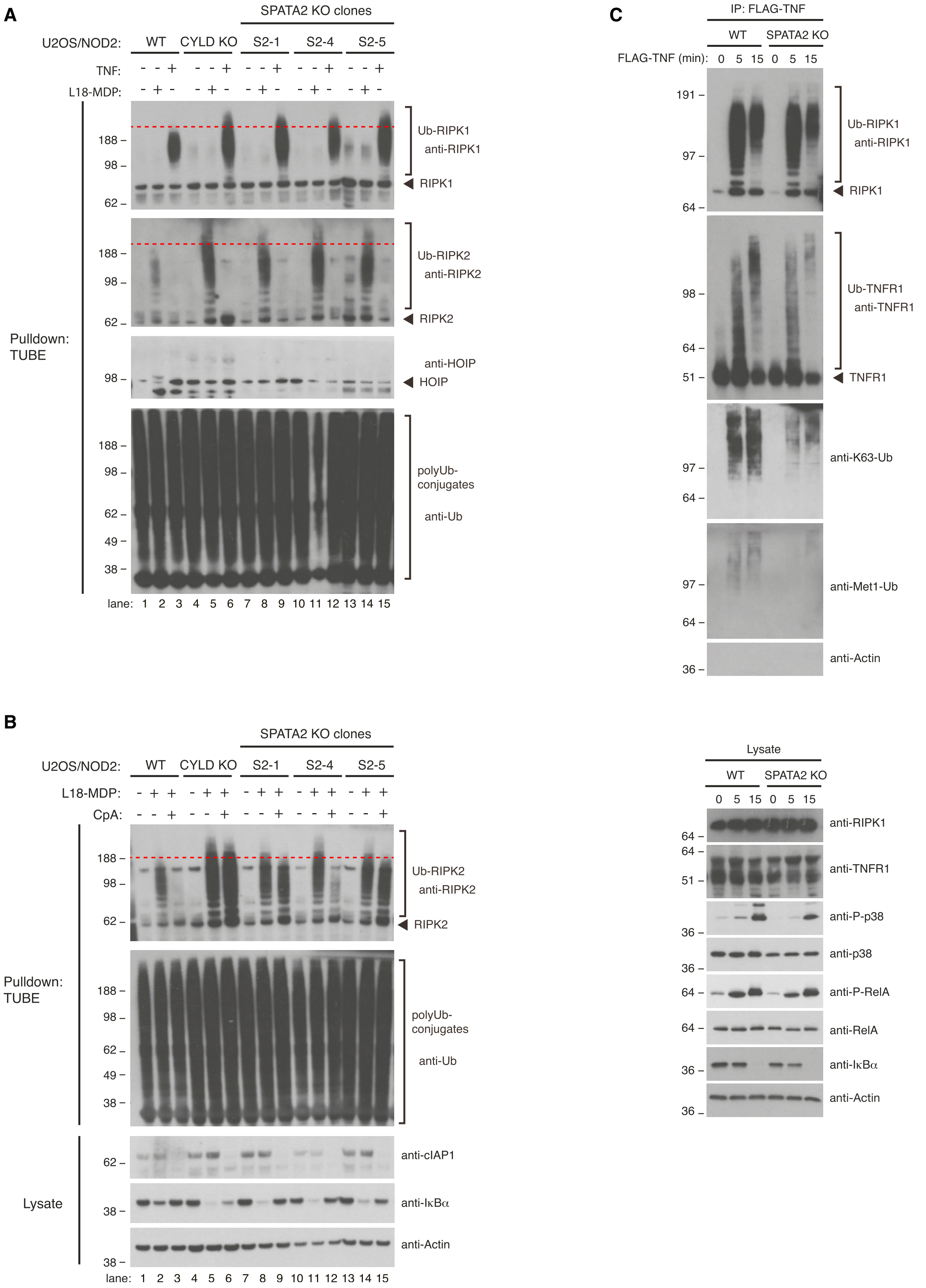

(legend on next page)

and components of the active TNFR1 complex (Draber et al., 2015; Hrdinka et al., 2016).

Thus, we propose a dynamic model where OTULIN and CYLD-SPATA2 are interchanged at the HOIP PUB interface, whereby the DUBs do not stably co-exist at the same LUBAC complex (Figure 7E). Quantitative studies of native LUBAC complexes will be needed to fully elucidate their composition and how DUB occupancy, and thereby LUBAC function, is regulated.

Our study reveals that the CYLD B-box, the role of which has remained elusive, is responsible for dimerization of the CYLD USP domain. HOIP contains a CYLD-like B-box following the PUB domain (Komander et al., 2008), and we have previously shown that a fragment spanning PUB, B-Box, and subsequent zinc-finger domains is able to self-associate (Elliott et al., 2014). B-box modules are also present in E3 ligases of the tripartite motif (TRIM) family, where at least in some cases they contribute to protein oligomerization (Wagner et al., 2016a). A crystal structure of the B-box from TRIM63/MuRF1 indicates a hydrophobic dimer interface as seen in CYLD (Mrosek et al., 2008). It will be interesting to see if the ability to form homotypic interactions is a general feature of B-box modules.

Strikingly, SPATA2 did not interact with monomeric CYLD ΔB-box, and while the CYLD-SPATA2 binding interface does not appear to involve the B-box itself, SPR measurements reveal diminished complex stability when CYLD is monomeric. It is possible that SPATA2 binding to a CYLD dimer creates additional interactions that stabilize the hetero-tetrameric complex, and further structural work is required to illuminate this.

Finally, the finding that SPATA2 activates CYLD adds SPATA2 to the growing list of allosteric DUB activators. Recent structural work revealed how the WD-repeat protein UAF1 interacted with the catalytic domain of USP46 and USP12, which is facilitated predominantly through the "fingers" subdomain of the USP core, mediating long-range allosteric interactions eventually leading to enhanced enzyme efficiency (Yin et al., 2015; Li et al., 2016). CYLD is structurally distinct from canonical USP domain folds in that it does not contain "fingers" but instead a B-box that binds the exposed side of the palm domain, but removal of which does not affect CYLD activity (Komander et al., 2008; Sato et al., 2015). The location of the SPATA2 binding site at the back of the palm domain suggests that SPATA2 activates CYLD in a distinct manner. In addition to SPATA2, SPATA2L also interacts with CYLD in cells (Sowa et al., 2009) (Figure 1E; Table S1) and may regulate other aspects of CYLD function. The fact that KO of SPATA2 already prevents association of CYLD with the TNFR1 (Figure 4D) indicates that SPATA2L cannot simply substitute for SPATA2, at least in the cellular systems tested.

In line with the biochemical data, our functional data indicate that SPATA2 plays a central regulatory role in LUBAC-mediated signaling, in particular in response to NOD2 stimulation, where loss of SPATA2 resulted in increased RIPK2 ubiquitination and productive NOD2 signaling. Moreover, it was striking that SPATA2 overexpression led to spurious IL-8 production in an SPATA2 PIM-dependent manner. Loss of SPATA2, however, had a less dramatic effect on some NOD2 responses than did the loss of CYLD. This could reflect that CYLD can regulate signaling independently of LUBAC binding, but that remains to be investigated. In addition to this, SPATA2 regulated ubiquitination following TNF treatment, and while this did not appear to greatly affect NF-κB signaling, it could well affect the formation of cell death-inducing complexes, as is the case for CYLD (Hitomi et al., 2008; O'Donnell et al., 2011). Indeed, during revision of this manuscript a study by Wagner et al. (2016b) showed that SPATA2 mediates necroptosis induced by TNF and caspase inhibition.

Conceptually, our study reveals that PUB-PIM interactions are at the core of how LUBAC function is regulated through its associated DUB activities, CYLD-SPATA2 and OTULIN. This adds SPATA2 to the still growing list of regulatory components of LUBAC and identifies a new modulator of important pathways involved in inflammation and infection. Future studies into the regulation of these interactions will be important for our understanding of how Met1-Ub regulates cellular signaling.

EXPERIMENTAL PROCEDURES

Please see the Supplemental Experimental Procedures for further details on all experimental procedures.

Protein Expression and Purification

HOIP and SPATA2 proteins were purified from E.coli as described (Elliott et al., 2014), and CYLD was expressed in Sf9 insect cells as described (Komander et al., 2008).

Crystallization and Structure Determination

Crystallization was performed using sparse matrix sitting drop vapor diffusion screening. The SPATA2 structure was determined by AMPLE (Bibby et al., 2012) using idealized helices, and the HOIP PUB-SPATA2 PIM complex by molecular replacement.

Binding Experiments

Details on gel filtration studies, SPR, and FP experiments can be found in the Supplemental Experimental Procedures.

Mass Spectrometry

Details on LC-MS/MS analysis of CYLD interactors can be found in the Supplemental Experimental Procedures.

DUB Assays

Qualitative gel-based DUB assays were performed as in Komander et al. (2009). Quantitative cleavage of Lys63-/Met1-linked diUb using fixed concentrations of FlAsH-labeled diUb was performed as in Keusekotten et al. (2013).

Figure 6. SPATA2 Mediates CYLD-Regulated NF-κB Responses

(A and B) Purification and western blot analysis of endogenous Ub conjugates from WT, CYLD KO, and SPATA2 KO (clone S2-1, S2-4, and S2-5) U2OS/NOD2 cells. Cells were treated with (A) TNF (5 ng/mL for 10 min) or L18-MDP (200 ng/mL for 1 hr), or (B) were pretreated with DMSO (control) or 1 μM compound A (CpA) for 30 min before stimulation with L18-MDP (200 ng/mL for 1 hr) as indicated.

(C) Purification and western blot analysis of the TNFR1 complex from WT and SPATA2 KO U2OS/NOD2 cells stimulated with FLAG-TNF (100 ng/mL) as indicated using anti-FLAG agarose.

Also see Figure S6.

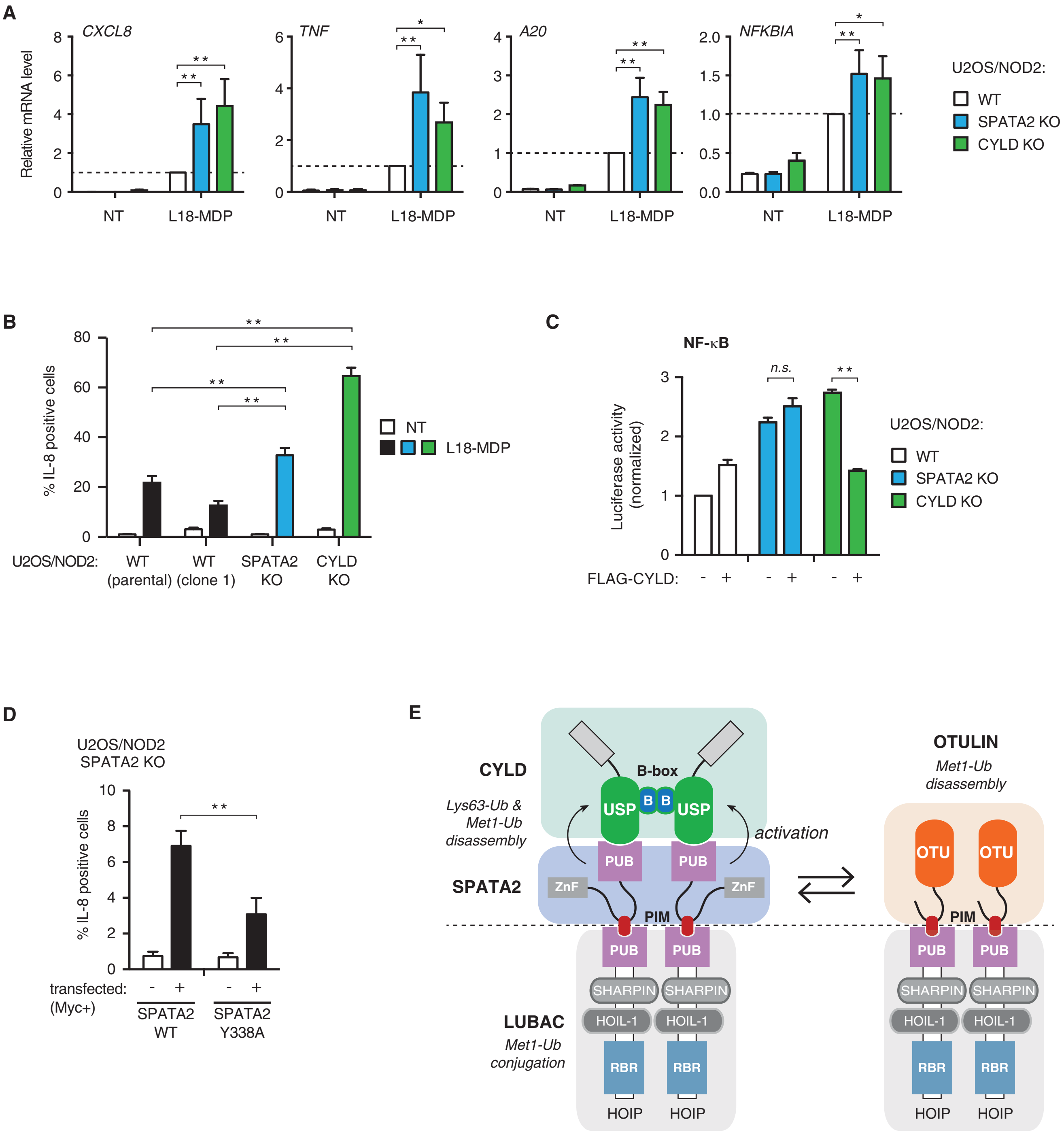

Figure 7. SPATA2 Mediates CYLD-Regulated NF-κB Responses

(A) Relative levels of *CXCL8*, *TNF*, *A20*, and *NFKBIA* transcripts measured by qRT-PCR on cDNA from WT, CYLD KO, and SPATA2 KO U2OS/NOD2 cells treated with L18-MDP (200 ng/mL for 3 hr).

(B) Intracellular flow cytometry analysis of IL-8 in WT (parental), WT (clone 1), SPATA2 KO, and CYLD KO U2OS/NOD2 cells. Cells were treated with L18-MDP (200 ng/mL) or vehicle for 5 hr along with Brefeldin A (5 μg/mL) and Monensin (2 μM) to block secretion of IL-8.

(C) NF-κB activity in WT, CYLD KO, and SPATA2 KO U2OS/NOD2 cell lysates transfected with dual luciferase reporters and co-expressed with FLAG-CYLD as indicated. Luciferase activity is shown relative to the activity of WT U2OS/NOD2 cells transfected with vector control. Data represent the mean ± SEM of six independent experiments, each performed in duplicate.

(legend continued on next page)

Purification of Endogenous Ub Conjugates

Ub conjugates were purified using GST-1xUBA^{ubq} Ub affinity reagent as in Fiil et al. (2013).

Characterization of NOD2 and TNF Signaling

Flow cytometry analysis of IL-8 production, quantitative real-time PCR, and purification of TNFR complexes were performed as described in Hrdinka et al. (2016).

Luciferase Reporter Assays

Cells were co-transfected the NF-κB luciferase reporter construct pBIIX-luc and a thymidine kinase-renilla luciferase construct. Additional plasmids were transfected as indicated and assays performed as in Damgaard et al. (2012).

Sucrose Gradient Sedimentation

Continuous 10%–40% sucrose gradients were generated using a GradientMaster 108 (Biocomp). Whole-cell lysate was subjected to velocity sedimentation on the sucrose gradients and fractioned before analysis by immunoblotting.

Generation of CRISPR/Cas9 Cell Lines

KO cell lines were created with the CRISPR/Cas9 KO (Santa Cruz) system containing gRNA, Cas9, and EGFP marker.

ACCESSION NUMBERS

The accession numbers for the coordinates and structure factors for the SPATA2 PUB domain and HOIP PUB- SPATA2 PIM structures are PDB: 5LJM and 5LJN, respectively.

SUPPLEMENTAL INFORMATION

Supplemental Information includes Supplemental Experimental Procedures, seven figures, and one table and can be found with this article online at http://dx.doi.org/10.1016/j.molcel.2016.08.001.

AUTHOR CONTRIBUTIONS

Conceptualization, M.G.-H. and D.K.; Investigation, P.R.E., D.L., M.H., K.B., J.W., S.M.V.F., B.K.F., S.H.M., and N.V.; Methodology, B.M.K. and J.C.C.; Writing – Original Draft, M.G.-H., D.K., P.R.E., and D.L.; Writing, M.G.-H., D.K., P.R.E., and D.L.; Funding Acquisition, M.G.-H. and D.K.

ACKNOWLEDGMENTS

This work was supported by the Ludwig Institute for Cancer Research Ltd. We thank Chris Johnson (MRC LMB) for biophysics support, and the beam line staff at DLS I02 for their assistance. Access to DLS was supported in part by the EU FP7 infrastructure grant BIOSTRUCT-X (contract no. 283570). We thank Roman Fischer, Rebecca Konietzny, and Simon Davis for expert help with the mass spectrometry analysis performed at the Advanced Proteomics Facility (headed by Roman Fischer), which is part of the TDI MS Laboratory. We thank Giovanni Stracquadanio (Ludwig institute for Cancer Research) for help with computational analysis of mass spectrometry data, TetraLogic Pharmaceuticals for compound A, and members of our groups for helpful suggestions and discussions. M.G.-H. is supported by a Wellcome Trust Fellowship (102894/Z/13/Z) and a Sapere Aude: Danish Council for Independent Research Starting Grant. Work in the D.K. lab is supported by the Medical Research Council (U105192732), the European Research Council (309756), and the Lister Institute for Preventive Medicine. M.G.-H. and D.K. are supported by the EMBO Young Investigator Programme. B.M.K. was funded by a John Fell Fund 133/075 and Wellcome Trust grant 097813/Z/11/Z. D.K. and B.M.K. are part of the DUB Alliance, which includes Cancer Research Technology and FORMA Therapeutics.

Received: May 16, 2016
Revised: July 14, 2016
Accepted: July 28, 2016
Published: August 30, 2016

REFERENCES

Bibby, J., Keegan, R.M., Mayans, O., Winn, M.D., and Rigden, D.J. (2012). AMPLE: a cluster-and-truncate approach to solve the crystal structures of small proteins using rapidly computed ab initio models. Acta Crystallogr. D Biol. Crystallogr. *68*, 1622–1631.

Boisson, B., Laplantine, E., Prando, C., Giliani, S., Israelsson, E., Xu, Z., Abhyankar, A., Israël, L., Trevejo-Nunez, G., Bogunovic, D., et al. (2012). Immunodeficiency, autoinflammation and amylopectinosis in humans with inherited HOIL-1 and LUBAC deficiency. Nat. Immunol. *13*, 1178–1186.

Boisson, B., Laplantine, E., Dobbs, K., Cobat, A., Tarantino, N., Hazen, M., Lidov, H.G., Hopkins, G., Du, L., Belkadi, A., et al. (2015). Human HOIP and LUBAC deficiency underlies autoinflammation, immunodeficiency, amylopectinosis, and lymphangiectasia. J. Exp. Med. *212*, 939–951.

Damgaard, R.B., Nachbur, U., Yabal, M., Wong, W.W., Fiil, B.K., Kastirr, M., Rieser, E., Rickard, J.A., Bankovacki, A., Peschel, C., et al. (2012). The ubiquitin ligase XIAP recruits LUBAC for NOD2 signaling in inflammation and innate immunity. Mol. Cell *46*, 746–758.

Damgaard, R.B., Fiil, B.K., Speckmann, C., Yabal, M., zur Stadt, U., Bekker-Jensen, S., Jost, P.J., Ehl, S., Mailand, N., and Gyrd-Hansen, M. (2013). Disease-causing mutations in the XIAP BIR2 domain impair NOD2-dependent immune signalling. EMBO Mol. Med. *5*, 1278–1295.

Draber, P., Kupka, S., Reichert, M., Draberova, H., Lafont, E., de Miguel, D., Spilgies, L., Surinova, S., Taraborrelli, L., Hartwig, T., et al. (2015). LUBAC-recruited CYLD and A20 regulate gene activation and cell death by exerting opposing effects on linear ubiquitin in signaling complexes. Cell Rep. *13*, 2258–2272.

Elliott, P.R., Nielsen, S.V., Marco-Casanova, P., Fiil, B.K., Keusekotten, K., Mailand, N., Freund, S.M., Gyrd-Hansen, M., and Komander, D. (2014). Molecular basis and regulation of OTULIN-LUBAC interaction. Mol. Cell *54*, 335–348.

Fiil, B.K., and Gyrd-Hansen, M. (2014). Met1-linked ubiquitination in immune signalling. FEBS J. *281*, 4337–4350.

Fiil, B.K., Damgaard, R.B., Wagner, S.A., Keusekotten, K., Fritsch, M., Bekker-Jensen, S., Mailand, N., Choudhary, C., Komander, D., and Gyrd-Hansen, M. (2013). OTULIN restricts Met1-linked ubiquitination to control innate immune signaling. Mol. Cell *50*, 818–830.

Gerlach, B., Cordier, S.M., Schmukle, A.C., Emmerich, C.H., Rieser, E., Haas, T.L., Webb, A.I., Rickard, J.A., Anderton, H., Wong, W.W., et al. (2011). Linear ubiquitination prevents inflammation and regulates immune signalling. Nature *471*, 591–596.

Haas, T.L., Emmerich, C.H., Gerlach, B., Schmukle, A.C., Cordier, S.M., Rieser, E., Feltham, R., Vince, J., Warnken, U., Wenger, T., et al. (2009). Recruitment of the linear ubiquitin chain assembly complex stabilizes the TNF-R1 signaling complex and is required for TNF-mediated gene induction. Mol. Cell *36*, 831–844.

(D) Intracellular flow cytometry analysis of IL-8 in SPATA2 KO U2OS/NOD2 cells transfected with Myc-SPATA2 variants as indicated. Cells were treated with Brefeldin A (5 μg/mL) and Monensin (2 μM) for 5 hr.
Data in (A), (B), and (D) represent mean ± SEM of at least three (A and B) or five (D) independent experiments. *p < 0.05, **p < 0.01; n.s., not significant.
(E) Schematic representation of LUBAC complexes with the proposed configuration for binding of SPATA2/CYLD or OTULIN.
Also see Figure S7.

Harhaj, E.W., and Dixit, V.M. (2012). Regulation of NF-κB by deubiquitinases. Immunol. Rev. *246*, 107–124.

Hitomi, J., Christofferson, D.E., Ng, A., Yao, J., Degterev, A., Xavier, R.J., and Yuan, J. (2008). Identification of a molecular signaling network that regulates a cellular necrotic cell death pathway. Cell *135*, 1311–1323.

Hrdinka, M., Fiil, B.K., Zucca, M., Leske, D., Bagola, K., Yabal, M., Elliott, P.R., Damgaard, R.B., Komander, D., Jost, P.J., and Gyrd-Hansen, M. (2016). CYLD limits Lys63- and Met1-linked ubiquitin at receptor complexes to regulate innate immune signaling. Cell Rep. *14*, 2846–2858.

Ikeda, F., Deribe, Y.L., Skånland, S.S., Stieglitz, B., Grabbe, C., Franz-Wachtel, M., van Wijk, S.J., Goswami, P., Nagy, V., Terzic, J., et al. (2011). SHARPIN forms a linear ubiquitin ligase complex regulating NF-κB activity and apoptosis. Nature *471*, 637–641.

Jiang, X., and Chen, Z.J. (2011). The role of ubiquitylation in immune defence and pathogen evasion. Nat. Rev. Immunol. *12*, 35–48.

Keusekotten, K., Elliott, P.R., Glockner, L., Fiil, B.K., Damgaard, R.B., Kulathu, Y., Wauer, T., Hospenthal, M.K., Gyrd-Hansen, M., Krappmann, D., et al. (2013). OTULIN antagonizes LUBAC signaling by specifically hydrolyzing Met1-linked polyubiquitin. Cell *153*, 1312–1326.

Kirisako, T., Kamei, K., Murata, S., Kato, M., Fukumoto, H., Kanie, M., Sano, S., Tokunaga, F., Tanaka, K., and Iwai, K. (2006). A ubiquitin ligase complex assembles linear polyubiquitin chains. EMBO J. *25*, 4877–4887.

Komander, D., Lord, C.J., Scheel, H., Swift, S., Hofmann, K., Ashworth, A., and Barford, D. (2008). The structure of the CYLD USP domain explains its specificity for Lys63-linked polyubiquitin and reveals a B box module. Mol. Cell *29*, 451–464.

Komander, D., Reyes-Turcu, F., Licchesi, J.D., Odenwaelder, P., Wilkinson, K.D., and Barford, D. (2009). Molecular discrimination of structurally equivalent Lys 63-linked and linear polyubiquitin chains. EMBO Rep. *10*, 466–473.

Kovalenko, A., Chable-Bessia, C., Cantarella, G., Israël, A., Wallach, D., and Courtois, G. (2003). The tumour suppressor CYLD negatively regulates NF-kappaB signalling by deubiquitination. Nature *424*, 801–805.

Li, H., Lim, K.S., Kim, H., Hinds, T.R., Jo, U., Mao, H., Weller, C.E., Sun, J., Chatterjee, C., D'Andrea, A.D., and Zheng, N. (2016). Allosteric activation of ubiquitin-specific proteases by β-propeller proteins UAF1 and WDR20. Mol. Cell *63*, 249–260.

Mrosek, M., Meier, S., Ucurum-Fotiadis, Z., von Castelmur, E., Hedbom, E., Lustig, A., Grzesiek, S., Labeit, D., Labeit, S., and Mayans, O. (2008). Structural analysis of B-Box 2 from MuRF1: identification of a novel self-association pattern in a RING-like fold. Biochemistry *47*, 10722–10730.

O'Donnell, M.A., Perez-Jimenez, E., Oberst, A., Ng, A., Massoumi, R., Xavier, R., Green, D.R., and Ting, A.T. (2011). Caspase 8 inhibits programmed necrosis by processing CYLD. Nat. Cell Biol. *13*, 1437–1442.

Reiley, W.W., Jin, W., Lee, A.J., Wright, A., Wu, X., Tewalt, E.F., Leonard, T.O., Norbury, C.C., Fitzpatrick, L., Zhang, M., and Sun, S.C. (2007). Deubiquitinating enzyme CYLD negatively regulates the ubiquitin-dependent kinase Tak1 and prevents abnormal T cell responses. J. Exp. Med. *204*, 1475–1485.

Ritorto, M.S., Ewan, R., Perez-Oliva, A.B., Knebel, A., Buhrlage, S.J., Wightman, M., Kelly, S.M., Wood, N.T., Virdee, S., Gray, N.S., et al. (2014). Screening of DUB activity and specificity by MALDI-TOF mass spectrometry. Nat. Commun. *5*, 4763.

Sahtoe, D.D., and Sixma, T.K. (2015). Layers of DUB regulation. Trends Biochem. Sci. *40*, 456–467.

Sato, Y., Goto, E., Shibata, Y., Kubota, Y., Yamagata, A., Goto-Ito, S., Kubota, K., Inoue, J., Takekawa, M., Tokunaga, F., and Fukai, S. (2015). Structures of CYLD USP with Met1- or Lys63-linked diubiquitin reveal mechanisms for dual specificity. Nat. Struct. Mol. Biol. *22*, 222–229.

Schaeffer, V., Akutsu, M., Olma, M.H., Gomes, L.C., Kawasaki, M., and Dikic, I. (2014). Binding of OTULIN to the PUB domain of HOIP controls NF-κB signaling. Mol. Cell *54*, 349–361.

Sowa, M.E., Bennett, E.J., Gygi, S.P., and Harper, J.W. (2009). Defining the human deubiquitinating enzyme interaction landscape. Cell *138*, 389–403.

Stieglitz, B., Haire, L.F., Dikic, I., and Rittinger, K. (2012). Structural analysis of SHARPIN, a subunit of a large multi-protein E3 ubiquitin ligase, reveals a novel dimerization function for the pleckstrin homology superfold. J. Biol. Chem. *287*, 20823–20829.

Takiuchi, T., Nakagawa, T., Tamiya, H., Fujita, H., Sasaki, Y., Saeki, Y., Takeda, H., Sawasaki, T., Buchberger, A., Kimura, T., and Iwai, K. (2014). Suppression of LUBAC-mediated linear ubiquitination by a specific interaction between LUBAC and the deubiquitinases CYLD and OTULIN. Genes Cells *19*, 254–272.

Tokunaga, F., Nakagawa, T., Nakahara, M., Saeki, Y., Taniguchi, M., Sakata, S., Tanaka, K., Nakano, H., and Iwai, K. (2011). SHARPIN is a component of the NF-κB-activating linear ubiquitin chain assembly complex. Nature *471*, 633–636.

Wagner, J.M., Roganowicz, M.D., Skorupka, K., Alam, S.L., Christensen, D., Doss, G., Wan, Y., Frank, G.A., Ganser-Pornillos, B.K., Sundquist, W.I., and Pornillos, O. (2016a). Mechanism of B-box 2 domain-mediated higher-order assembly of the retroviral restriction factor TRIM5α. eLife *5*, http://dx.doi.org/10.7554/eLife.16309.

Wagner, S.A., Satpathy, S., Beli, P., and Choudhary, C. (2016b). SPATA2 links CYLD to the TNF-α receptor signaling complex and modulates the receptor signaling outcomes. EMBO J., Published online June 15, 2016. http://dx.doi.org/10.15252/embj.201694300.

Yin, J., Schoeffler, A.J., Wickliffe, K., Newton, K., Starovasnik, M.A., Dueber, E.C., and Harris, S.F. (2015). Structural insights into WD-repeat 48 activation of ubiquitin-specific protease 46. Structure *23*, 2043–2054.

Zhao, G., Zhou, X., Wang, L., Li, G., Schindelin, H., and Lennarz, W.J. (2007). Studies on peptide:N-glycanase-p97 interaction suggest that p97 phosphorylation modulates endoplasmic reticulum-associated degradation. Proc. Natl. Acad. Sci. USA *104*, 8785–8790.

Molecular Cell, Volume *63*

Supplemental Information

SPATA2 Links CYLD to LUBAC, Activates CYLD, and Controls LUBAC Signaling

Paul R. Elliott, Derek Leske, Matous Hrdinka, Katrin Bagola, Berthe K. Fiil, Stephen H. McLaughlin, Jane Wagstaff, Norbert Volkmar, John C. Christianson, Benedikt M. Kessler, Stefan M.V. Freund, David Komander, and Mads Gyrd-Hansen

Figure S1

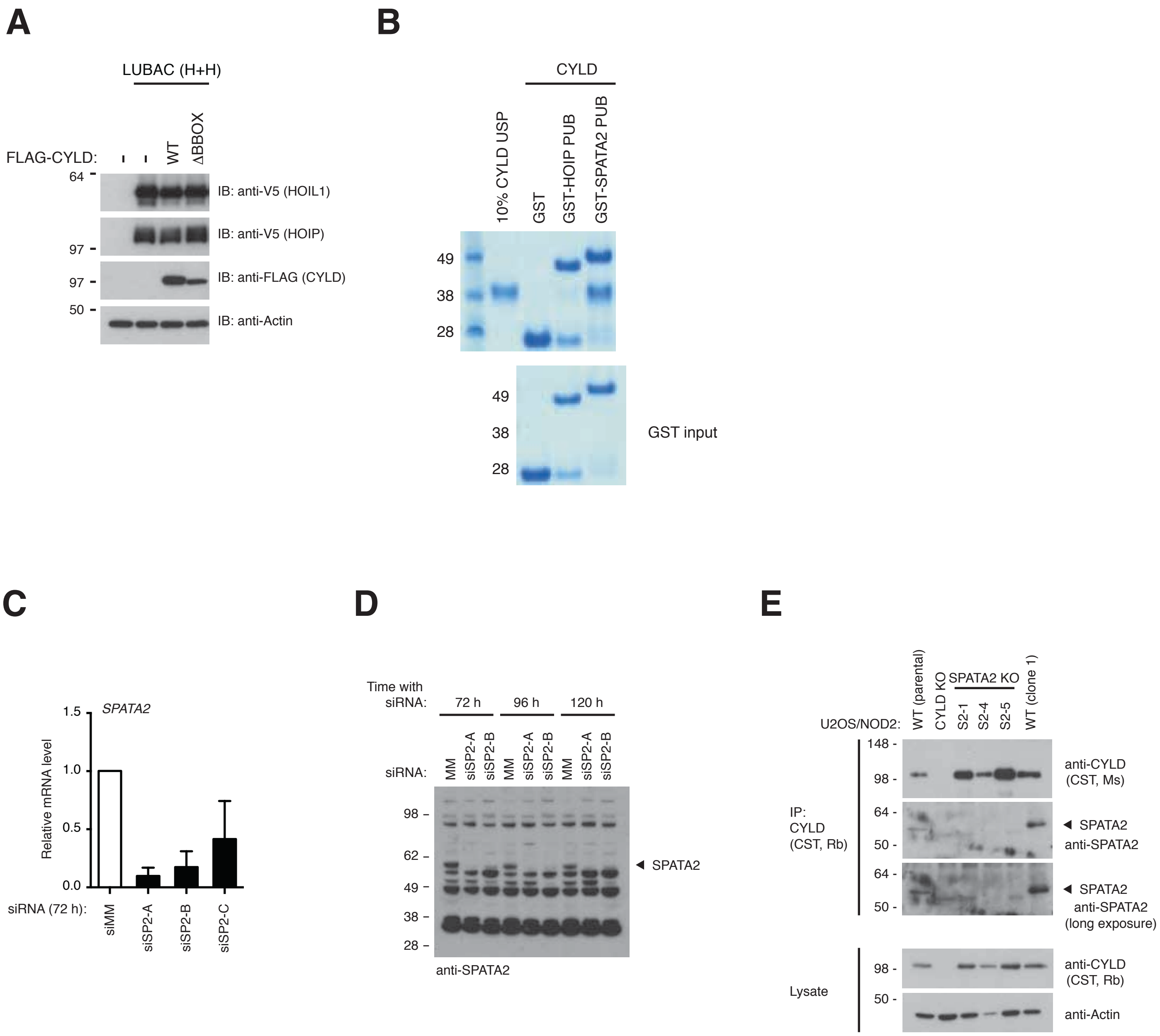

A
B
LUBAC (H+H)
FLAG-CYLD:
WT
ΔBBOX
64
97
97
50
IB: anti-V5 (HOIL1)
IB: anti-V5 (HOIP)
IB: anti-FLAG (CYLD)
IB: anti-Actin
CYLD
10% CYLD USP
GST
GST-HOIP PUB
GST-SPATA2 PUB
49
38
28
GST input
C
D
E
SPATA2
Relative mRNA level
1.5
1.0
0.5
0.0
siRNA (72 h):
siMM
siSP2-A
siSP2-B
siSP2-C
Time with siRNA:
72 h
96 h
120 h
siRNA:
MM
siSP2-A
siSP2-B
98
62
49
38
28
SPATA2
anti-SPATA2
U2OS/NOD2:
WT (parental)
CYLD KO
SPATA2 KO
S2-1
S2-4
S2-5
WT (clone 1)
148
98
64
50
IP: CYLD (CST, Rb)
anti-CYLD (CST, Ms)
anti-SPATA2
anti-SPATA2 (long exposure)
Lysate
anti-CYLD (CST, Rb)
anti-Actin

Figure S1:, related to Figure 1

(A) CYLD KO U2OS/NOD2 cell lysates from experiment shown in Figure 1C were analyzed by western blot analysis as indicated. (B) Coomassie stained GST pull-down experiments using GST, GST-HOIP PUB or GST-SPATA2 PUB and CYLD USP domain. (C-E) Characterization of SPATA2 antibody used in study and confirmation of knockout U2OS/NOD2 cell lines generated using CRISPR/Cas9. (C) Relative *SPATA2* mRNA levels following knockdown with *SPATA2* siRNA (SP2-A, SP2-B, and SP2-C) in U2OS/NOD2 cells. Values shown represents mean ± SEM from three independent experiments. (D) Western blot analysis of SPATA2 levels in U2OS/NOD2 cell lysates following knockdown by MM (control), SP2-A, and SP2-B siRNA for 72 h, 96 h, and 120 h (as indicated). (E) Immunoprecipitation and western blot analysis of endogenous CYLD in WT, CYLD KO, SPATA2 KO clones (S2-1, S2-4, and S2-5), and S3-5 (selected WT clone) to determine SPATA2 status.

Figure S2

A

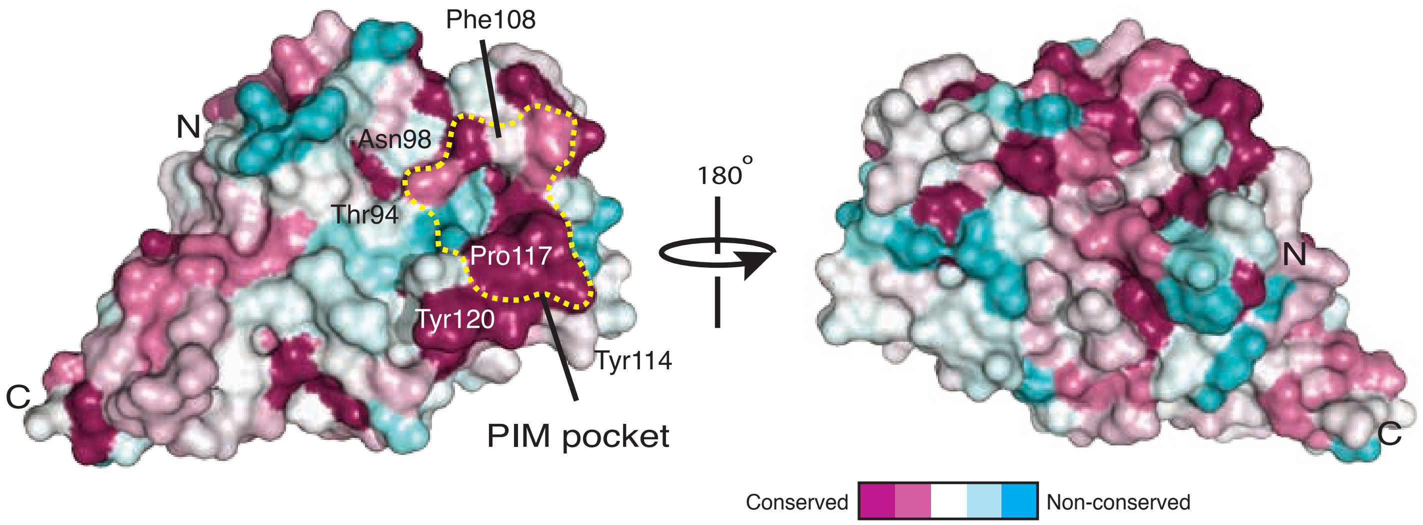

B

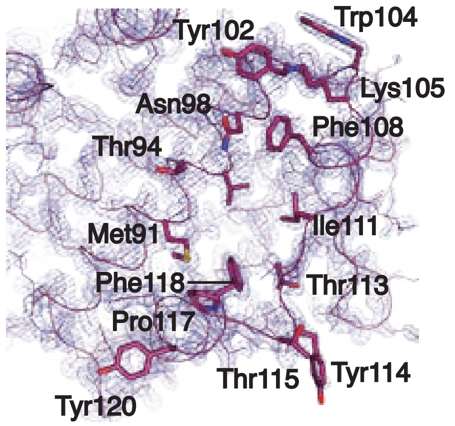

C

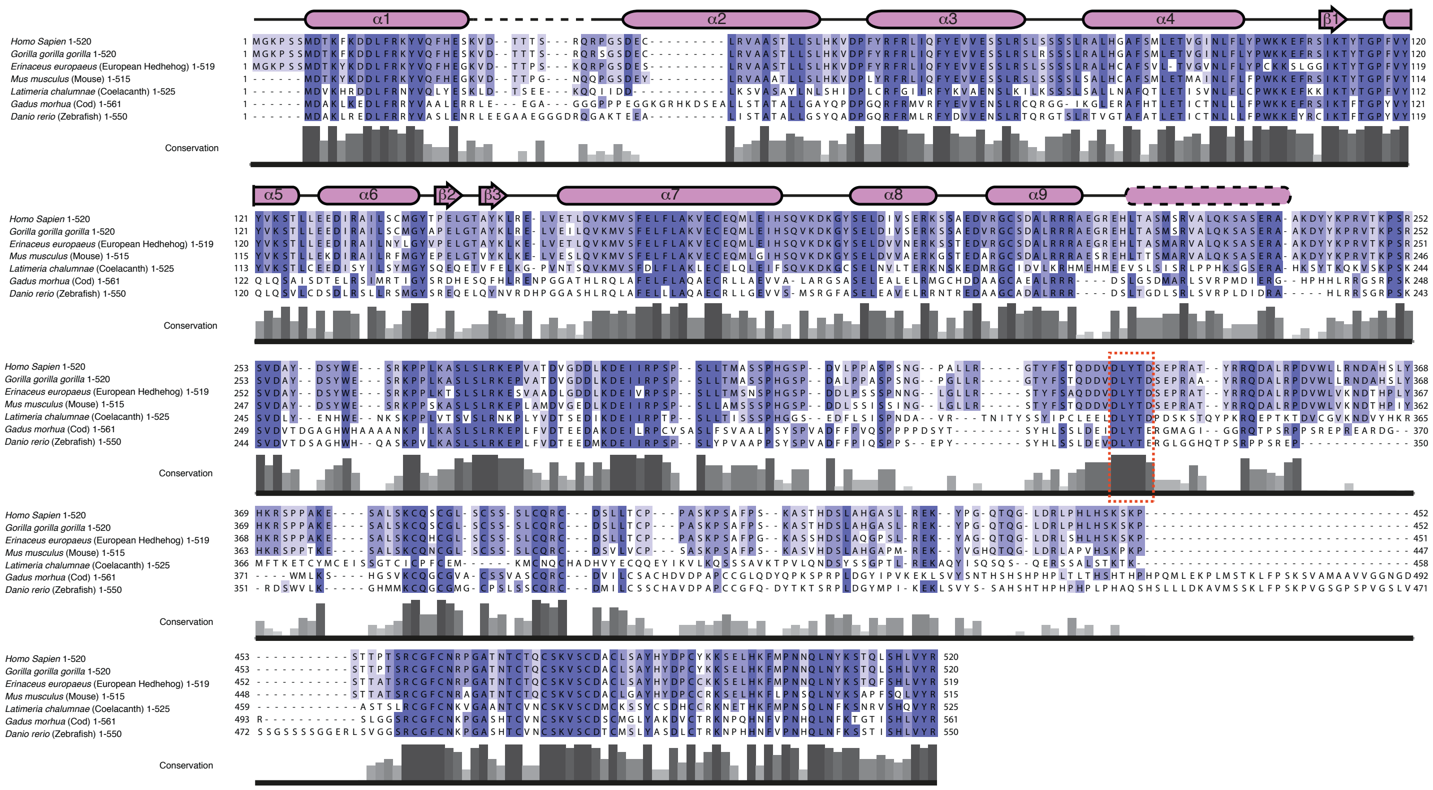

Figure S2:, related to Figure 2

(A) Surface conservation analysis of the SPATA2 PUB domain colored according to sequence conservation. The PIM pocket is highlighted, as are key residues around the PIM pocket and CYLD binding site. (B) A weighted 2|Fo|-|Fc| map contoured at 0.9 σ of the PIM pocket of SPATA2. (C) Sequence alignment of SPATA2 showing the sequences of distant orthologs. The sequence conservation between all SPATA2 orthologs is shown as bars. Secondary structure of the PUB domain is shown above the alignment. The PIM peptide is shown (red box). A predicted tenth alpha helix based upon secondary structure analysis is shown where the longer SPATA2 PUB domain (1-241) ends. This fragment behaves better during purification and is used in biophysical analysis.

Figure S3

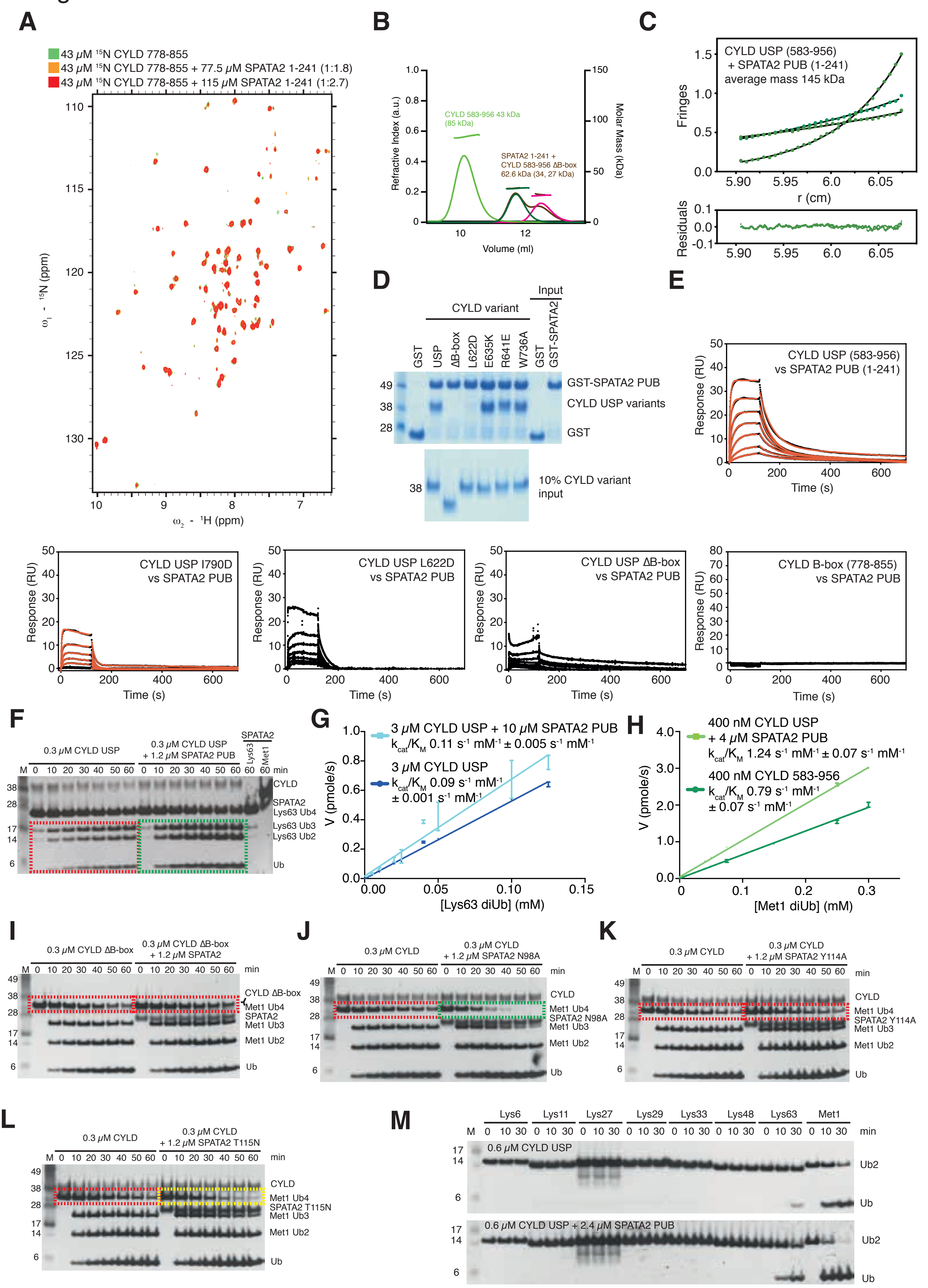

A
43 μM 15N CYLD 778-855
43 μM 15N CYLD 778-855 + 77.5 μM SPATA2 1-241 (1:1.8)
43 μM 15N CYLD 778-855 + 115 μM SPATA2 1-241 (1:2.7)
ω1 - 15N (ppm)
ω2 - 1H (ppm)
B
CYLD 583-956 43 kDa (85 kDa)
SPATA2 1-241 + CYLD 583-956 ΔB-box 62.6 kDa (34, 27 kDa)
Refractive Index (a.u.)
Molar Mass (kDa)
Volume (ml)
C
CYLD USP (583-956) + SPATA2 PUB (1-241) average mass 145 kDa
Fringes
r (cm)
Residuals
D
CYLD variant
Input
GST USP ΔB-box L622D E635K R641E W736A GST GST-SPATA2
GST-SPATA2 PUB
CYLD USP variants
GST
10% CYLD variant input
E
CYLD USP (583-956) vs SPATA2 PUB (1-241)
Response (RU)
Time (s)
CYLD USP I790D vs SPATA2 PUB
CYLD USP L622D vs SPATA2 PUB
CYLD USP ΔB-box vs SPATA2 PUB
CYLD B-box (778-855) vs SPATA2 PUB
F
0.3 μM CYLD USP
0.3 μM CYLD USP + 1.2 μM SPATA2 PUB
SPATA2
Lys63 Met1
M 0 10 20 30 40 50 60 0 10 20 30 40 50 60 60 60 min
CYLD
SPATA2
Lys63 Ub4
Lys63 Ub3
Lys63 Ub2
Ub
G
3 μM CYLD USP + 10 μM SPATA2 PUB
kcat/KM 0.11 s-1 mM-1 ± 0.005 s-1 mM-1
3 μM CYLD USP
kcat/KM 0.09 s-1 mM-1 ± 0.001 s-1 mM-1
V (pmole/s)
[Lys63 diUb] (mM)
H
400 nM CYLD USP + 4 μM SPATA2 PUB
kcat/KM 1.24 s-1 mM-1 ± 0.07 s-1 mM-1
400 nM CYLD 583-956
kcat/KM 0.79 s-1 mM-1 ± 0.07 s-1 mM-1
V (pmole/s)
[Met1 diUb] (mM)
I
0.3 μM CYLD ΔB-box
0.3 μM CYLD ΔB-box + 1.2 μM SPATA2
CYLD ΔB-box
Met1 Ub4
SPATA2
Met1 Ub3
Met1 Ub2
Ub
J
0.3 μM CYLD
0.3 μM CYLD + 1.2 μM SPATA2 N98A
CYLD
Met1 Ub4
SPATA2 N98A
Met1 Ub3
Met1 Ub2
Ub
K
0.3 μM CYLD
0.3 μM CYLD + 1.2 μM SPATA2 Y114A
CYLD
Met1 Ub4
SPATA2 Y114A
Met1 Ub3
Met1 Ub2
Ub
L
0.3 μM CYLD
0.3 μM CYLD + 1.2 μM SPATA2 T115N
CYLD
Met1 Ub4
SPATA2 T115N
Met1 Ub3
Met1 Ub2
Ub
M
Lys6 Lys11 Lys27 Lys29 Lys33 Lys48 Lys63 Met1
M 0 10 30 0 10 30 0 10 30 0 10 30 0 10 30 0 10 30 0 10 30 0 10 30 min
0.6 μM CYLD USP
0.6 μM CYLD USP + 2.4 μM SPATA2 PUB
Ub2
Ub

Figure S3:, related to Figure 3

(A) BEST-TROSY spectra of ^{15}N-labelled CYLD B-box (778-855) (green) mixed with 1:1.8 molar excess (orange) and 1:2.7 molar excess of SPATA2 PUB domain (red), showing no perturbations of B-box resonances, indicative of no or very weak binding. (B) SEC-MALS profile for CYLD USP (green), CYLD USP ΔB-box (blue), SPATA2 PUB (red) and CYLD USP ΔB-box mixed with SPATA2 PUB (brown). The separate elution profiles of the USP ΔB-box and SPATA2 PUB domain are in agreement with the elution profiles and molecular weights of individual components, confirming lack of complex formation. (C) Sedimentation equilibrium analytical ultracentrifugation analysis of the CYLD USP–SPATA2 PUB complex. 1 μM of complex was centrifuged at 5,700, 6,900 and 12,000 rpm at 20 °C until equilibration was reached. The fringe pattern is plotted against the position within the cell and the residuals are shown below. The calculated Mwt of the complex is 145 kDa, consistent with a 2:2 dimer (predicted Mwt 142 kDa). (D) Pull-down using GST-SPATA2 PUB and different CYLD variants. (E) SPR sensorgrams for CYLD variant binding to immobilized SPATA2 PUB. Fits of the raw data for determining the kinetic dissociation constant (K^{kin}_{d}) are shown in red. (F) DUB assay, similar to **Figure 3E**, following Lys63-linked tetra-Ub cleavage by CYLD in the absence or presence of SPATA2 PUB domain. The final two lanes demonstrate that SPATA2 does not contain any protease activity against Lys63- and Met1-linked tetraUb. A small amount of contaminating Lys63-linked di- and triUb are found in the Lys63-linked tetraUb sample at time zero and does not increase overtime unless CYLD is present. (G-H) Kinetic analysis using Lys63-linked diUb (G) or Met1-linked diUb (H) and a fixed concentration of 150 nM Lys63/Met1 FlAsH-tagged diUb. Initial rates of substrate cleavage are plotted against substrate concentration and fitted to yield CYLD activity in the absence or presence of SPATA2 PUB. Error bars represent standard deviation from the mean of measurements performed in triplicate. (I) DUB assay similar to **Figures 3E-F,** using CYLD USP ΔB-box. Removal of the B-box domain does not affect CYLD DUB activity. However, CYLD ΔB-box is unable to be activated by SPATA2 PUB. (J-L) DUB assays, using Met1-linked tetraUb and SPATA2 PUB mutant that still binds and activates CYLD (N98A, J), abolish CYLD binding and activation (Y114A, K) and have an intermediate effect on CYLD activation (T115N, L). (M) DUB assay using 0.6 μM CYLD USP and all eight linked diUb in the absence of (top) and the presence of 2.4 μM SPATA2 (bottom) showing an enhancement in CYLD activity by SPATA2 but no change in Ub linkage specificity by CYLD.

Figure S4

A

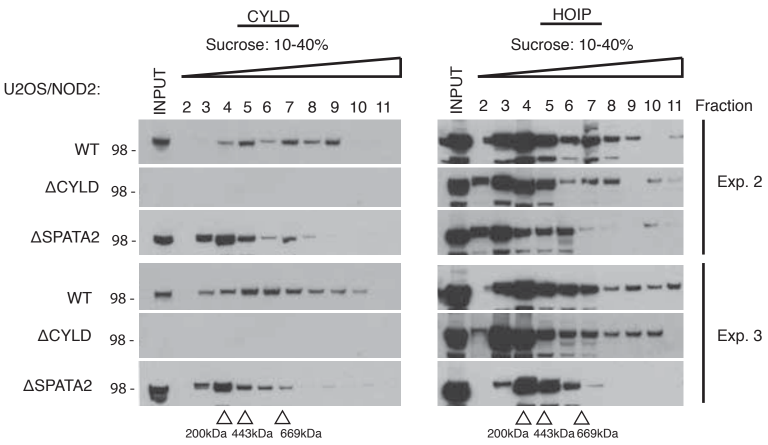

CYLD
HOIP
Sucrose: 10-40%
Sucrose: 10-40%
U2OS/NOD2:
INPUT
2 3 4 5 6 7 8 9 10 11
INPUT
2 3 4 5 6 7 8 9 10 11
Fraction
WT 98 -
ΔCYLD 98 -
ΔSPATA2 98 -
Exp. 2
WT 98 -
ΔCYLD 98 -
ΔSPATA2 98 -
Exp. 3
200kDa 443kDa 669kDa
200kDa 443kDa 669kDa

Figure S4:, related to Figure 4
(A) Sucrose centrifugation sedimentation (experiments two and three) and western blot analysis of lysates from WT, CYLD KO, and SPATA2 KO U2OS/NOD2 cells as in **Figure 4E**. The shown blots together with the blots in Figure 4E were subjected to image densitometry using ImageJ software. Raw values in each fraction were normalized and means ± SEM are depicted in Figure 4E, bottom.

Figure S5

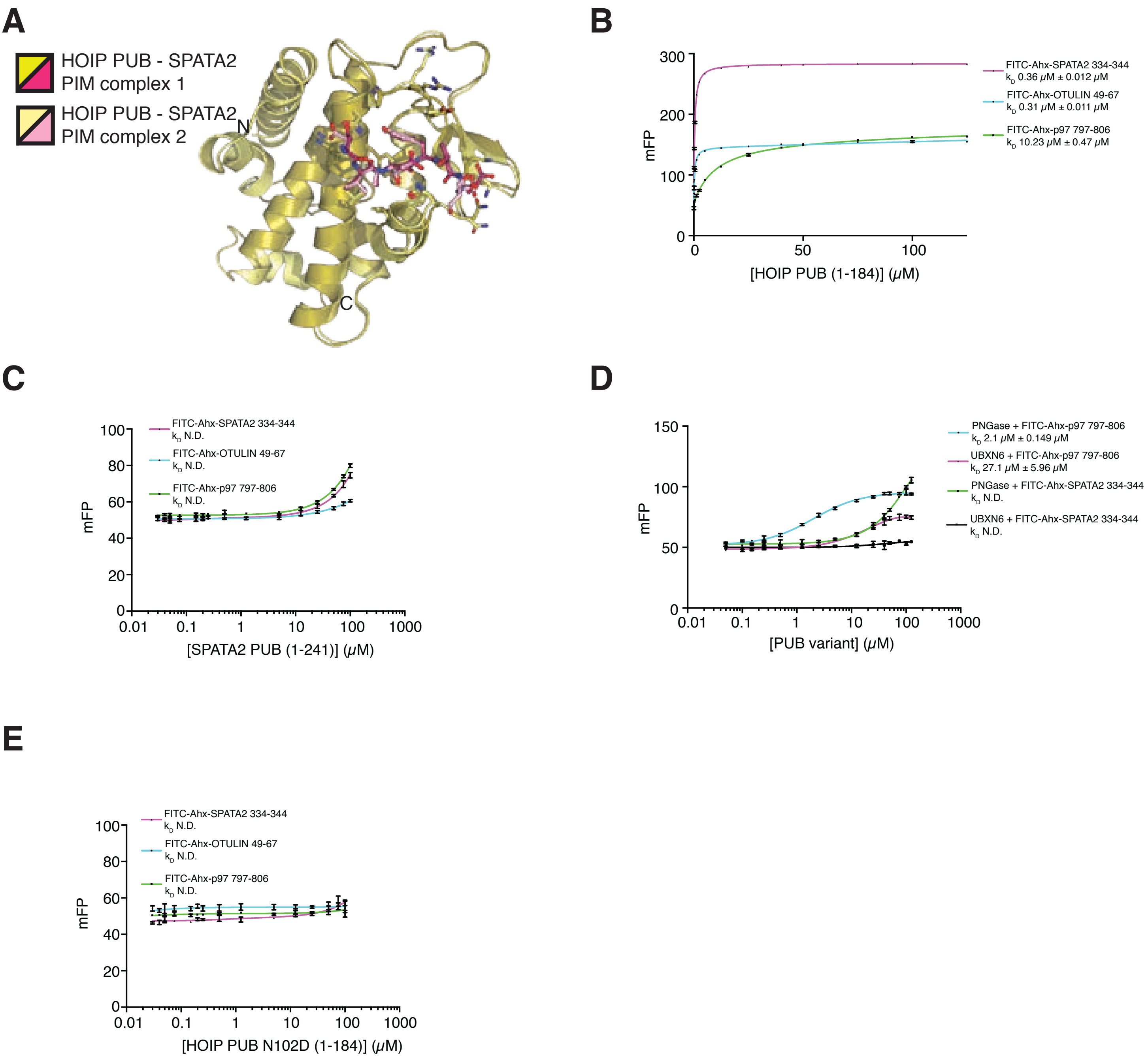

A
HOIP PUB - SPATA2 PIM complex 1
HOIP PUB - SPATA2 PIM complex 2
N
C
B
FITC-Ahx-SPATA2 334-344
k_D 0.36 μM ± 0.012 μM
FITC-Ahx-OTULIN 49-67
k_D 0.31 μM ± 0.011 μM
FITC-Ahx-p97 797-806
k_D 10.23 μM ± 0.47 μM
mFP
[HOIP PUB (1-184)] (μM)
C
FITC-Ahx-SPATA2 334-344
k_D N.D.
FITC-Ahx-OTULIN 49-67
k_D N.D.
FITC-Ahx-p97 797-806
k_D N.D.
mFP
[SPATA2 PUB (1-241)] (μM)
D
PNGase + FITC-Ahx-p97 797-806
k_D 2.1 μM ± 0.149 μM
UBXN6 + FITC-Ahx-p97 797-806
k_D 27.1 μM ± 5.96 μM
PNGase + FITC-Ahx-SPATA2 334-344
k_D N.D.
UBXN6 + FITC-Ahx-SPATA2 334-344
k_D N.D.
mFP
[PUB variant] (μM)
E
FITC-Ahx-SPATA2 334-344
k_D N.D.
FITC-Ahx-OTULIN 49-67
k_D N.D.
FITC-Ahx-p97 797-806
k_D N.D.
mFP
[HOIP PUB N102D (1-184)] (μM)

Figure S5:, related to Figure 5

(A) Superimposition of the two HOIP PUB – SPATA2 PIM structures from the asymmetric unit. (B) Affinity measurements using HOIP PUB domain against FITC-Ahx-p97 797-806 (green), FITC-Ahx-OTULIN 49-67 (cyan) and FITC-Ahx-SPATA2 334-344 (magenta). (C) Affinity measurements as in (B) using SPATA2 PUB domain. (D) Affinity measurements using either PUB domains of PNGase or UBXN6. (E) Mutation of the cornerstone residue Asn102 in HOIP abolishes all PIM binding, including SPATA2. All measurements were performed in triplicate and errors represent the standard deviation from the mean.

Figure S6

A

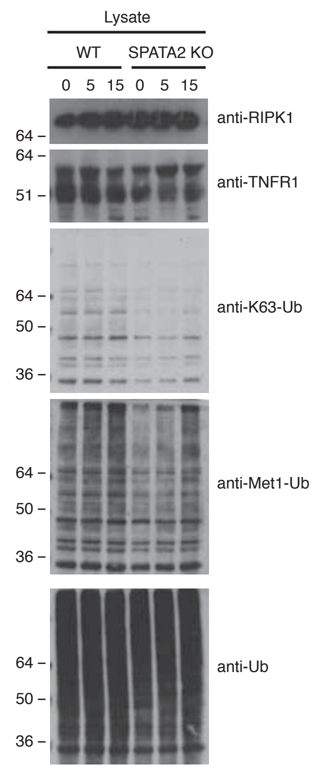
Lysate
WT
SPATA2 KO
0 5 15 0 5 15
anti-RIPK1
anti-TNFR1
anti-K63-Ub
anti-Met1-Ub
anti-Ub
64
64
51
64
50
36
64
50
36
64
50
36

Figure S6:, related to Figure 6

(A) WT and SPATA2 KO U2OS/NOD2 cell lysates from FLAG-TNF immunoprecipitation shown in **Figure 6A** were analyzed by western blot as indicated.

Figure S7

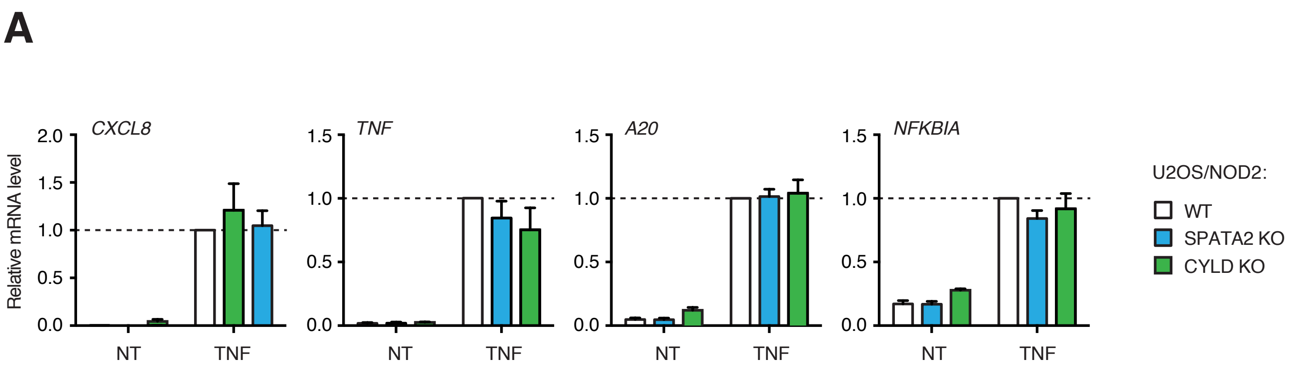
A
CXCL8
TNF
A20
NFKBIA
Relative mRNA level
2.0
1.5
1.0
0.5
0.0
NT
TNF
U2OS/NOD2:
WT
SPATA2 KO
CYLD KO

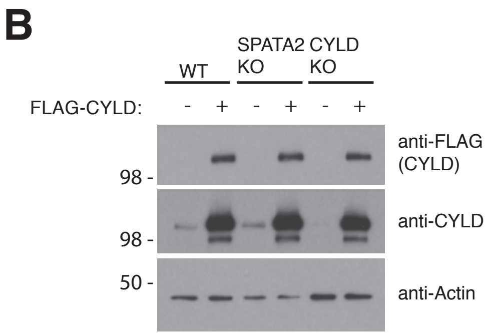
B
WT
SPATA2 KO
CYLD KO
FLAG-CYLD:
- + - + - +
anti-FLAG (CYLD)
anti-CYLD
anti-Actin
98 -
98 -
50 -

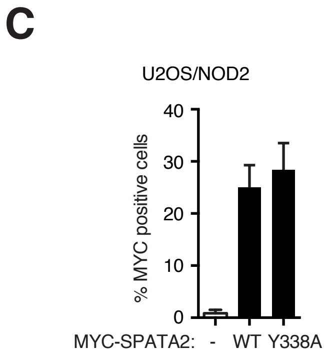
C
U2OS/NOD2
% MYC positive cells
40
30
20
10
0
MYC-SPATA2:
-
WT
Y338A

Figure S7:, related to Figure 7

(A) Relative levels of *CXCL8*, *TNF*, *A20*, and *NFKBIA* transcripts measured by qRT-PCR on cDNA from WT, CYLD KO, and SPATA2 KO U2OS/NOD2 cells treated with TNF (5ng/ml for 3 h). (B) WT, CYLD KO, and SPATA2 KO U2OS/NOD2 cell lysates from experiment shown in **Figure 7C** were analyzed by western blot analysis as indicated. (C) Intracellular flow cytometry analysis of Myc-positive cells in SPATA2 KO U2OS/NOD2 cells transfected with Myc-SPATA2 variants as indicated. Cells were treated with Brefeldin A (5 μg/ml) and Monensin (2 μM) for 5 h. Data represents mean ± SEM of three independent experiments.

Extended Experimental Procedures

Sequence analysis
Multiple alignment analysis shown in **Figure 5A** was performed with ClustalX on SPATA2 sequences (NCBI accession): Hs (Homo sapiens); Q9UM82, Pt (Pan troglodytes); H2QKJ9, Mam (Macaca mulatta); F6YDM2, Mm (Mus musculus); Q8K004, Rn (Rattus norvegicus); Q91XS7, Clf (Canis lupus familiaris); F1PY42, Bt (Bos taurus); E1BLR1, Eq (Equus caballus); F7CMQ6, Ss (Sus scrofa); A0A0B8RVG9, Xt (Xenopus tropicalis); F7EKQ2, Gg (Gallus gallus); Q5ZI97.

Plasmids and cloning
The following plasmids used in the study have been described previously; pcDNA3-HOIP-V5/His and pcDNA3-HOIL-1-V5/His (Haas et al., 2009). pcDNA3-HOIP PUB+NZF-V5/His and pcDNA3-HOIP N102D-V5/His, pCMV2-FLAG-CYLD, pCMV2-FLAG-CYLD(C601A), pGEX-6P-1-Ubiquillin-UBAx, and the NF-κB luciferase reporter plasmids pBIIX-Luc and TK-renilla-Luc (Hrdinka et al., 2016). CYLD ΔB-box was generated by overlap extension and splicing PCR using pCMV2-FLAG-CYLD as template. pcDNA3.1 Myc-SPATA was generated by PCR amplification from cDNA and insertion into pcDNA3.1 vector. All SPATA2 variants were created by PCR-based site-directed mutagenesis using pcDNA3.1 MYC-SPATA2 as template.
For biochemical and structural studies the coding sequence of full-length SPATA2 was amplified from a brain cDNA library (Clontech) using KOD HotStart DNA polymerase and the following forward and reverse primers: AAGTTCTGTTTCAGGGCCCGATGGGGAAGCCCAGTTCAATG and ATGGTCTAGAAAGCTTTATCTGTACACGAGATGGGAGAG respectively. The PCR product was cloned into pOPINK, which encodes a 3C cleavable N-terminal His6-GST-tag (Berrow et al., 2007) using Infusion HD cloning (Clontech) and was verified by DNA sequencing.

Antibodies and immunoprecipitation reagents
The following antibodies and reagents were used according to the manufacturers' instructions: mouse monoclonal anti-β-Actin (Chemicon Millipore, Billerica, MA), rat monoclonal anti-cIAP1 (Enzo Life Sciences, Exeter, UK), rabbit polyclonal anti-CYLD (Cell Signaling Technology, Danvers, MA), mouse monoclonal anti-CYLD (Santa Cruz Biotechnology, Santa Cruz, CA; used for immunoblotting in **Figure 1G**; **Figure S1E**), monoclonal mouse-anti-FLAG (Sigma-Aldrich, Gillingham, UK), rabbit polyclonal anti-HOIL-1/RBCK1 (Novus Biologicals, Littleton, CO), rabbit polyclonal anti-HOIP/RNF31 (Sigma-Aldrich), rabbit polyclonal anti-HOIP/RNF31 (Ubiquigent, Dundee, UK), rabbit polyclonal anti-IκBα, (Cell Signaling Technology), control rabbit IgG (Santa Cruz Biotechnology), rabbit polyclonal anti-OTULIN (Abcam, Cambridge, UK), rabbit monoclonal anti-RIPK1 (Cell Signaling Technology), rabbit polyclonal anti-RIPK2 (Santa Cruz Biotechnology), rabbit-polyclonal anti-SHARPIN (Proteintech, Rosemont, IL), rabbit polyclonal anti-SPATA2 (Bethyl Laboratories, Montgomery, TX), mouse monoclonal anti-SPATA2 (Santa Cruz Biotechnology used for immunoblotting in **Figure 1G** and **1H**) monoclonal rabbit anti-TNFR1 (Cell Signaling Technology)**,** mouse monoclonal anti-Tubulin (Clone DM1A, Sigma Aldrich), mouse monoclonal anti-Ubiquitin (Imgenex, San Diego, CA), mouse monoclonal anti-Ubiquitin (clone P4D1, Cell Signaling Technology), mouse monoclonal anti-V5 (AbD Serotec, Kidlington, UK)**.** HRP-conjugated secondary antibodies were from Bio-Rad, Hercules, CA (anti-rabbit), Dako, Glostrup, DK (anti-mouse), and R&D systems, Minneapolis, MN (anti-sheep). For immunoprecipitation, anti-HA-agarose conjugate (Clone HA-7, Sigma Aldrich), anti-FLAG affinity gel (Clone M2, Sigma Aldrich) and Protein A/G plus agarose (Santa Cruz Biotechnology) was used. For intracellular staining of IL-8 for flow cytometry the APC conjugated mouse anti-human IL-8 Antibody (clone E8N1, BioLegend, SanDiego, CA) was used.

Cell lines
NOD2-expressing U2OS-Flp-In™ T-REx™ (U2OS/NOD2) cells were cultured in DMEM GlutaMax (Gibco Life Technologies, Carlsbad, CA) supplemented with 10% (v/v) FBS (Sigma-Aldrich) and 1% (v/v) Penicillin-Streptomycin (Gibco Life Technologies) and transfected using Fugene 6 (Promega). Throughout the study, the U2OS/NOD2 cells were cultured and stimulated in the absence of doxycycline unless otherwise indicated. HCT116 cells were cultured in McCoy's 5A (modified) GlutaMax (Gibco®), supplemented with 10% FBS and 1% Penicillin-Streptomycin (Gibco®).

Purification of endogenous ubiquitin conjugates
Ubiquitin conjugates were purified from U2OS/NOD2 cells treated with 200 ng/ml L18-MDP (Invivogen, San Diego, CA) and/or 1 μM Compound A (a kind gift from Tetralogic Therapeutics) using GST-1xUBA^{ubq} ubiquitin affinity reagent (TUBE) (Fiil et al., 2013). Briefly, cells were lysed in TUBE lysis buffer (20 mM Na_2HPO_4, 20 mM NaH_2PO_4, 1% (v/v) NP-40, 2 mM EDTA) supplemented with 1 mM DTT (Sigma Aldrich), 5 mM N-Ethylmaleimide (NEM; Sigma Aldrich), cOmplete protease inhibitors (Roche Diagnostics, Burgess Hill,

UK), and PhosSTOP (Roche Diagnostics). TUBE (50 μg/ml) was added pre-bound to Glutathione Sepharose 4B beads (GE Healthcare) for at least 1 hr. Lysates were cleared by centrifugation, mixed with beads and incubated agitating at 4°C for a minimum of 2 hr. The beads were washed four times in 500 μl of ice-cold PBS 0.1% (v/v) Tween-20. The bound material was eluted with 1x lysis sample buffer.

Intracellular flow cytometry of IL-8

U2OS/NOD2 cells were stimulated with 200 ng/ml L18-MDP for 5 h in the presence of 5 μg/ml Brefeldin A (BioLegend, San Diego, CA) and 2 μM Monensin (BioLegend,) protein transport inhibitors. After stimulation, cells were washed with PBS, dissociated by Trypsin/EDTA solution (Gibco Life Technologies), and collected by centrifugation. Cells were fixed with IC Fixation Buffer (BioLegend) O/N at 4°C, washed with PBS, permeabilized using Perm/Wash Buffer containing 2% (v/v) FCS, 0.1% (w/v) saponin and 0.1% (w/v) NaN_3 in PBS and incubated in the Perm/Wash Buffer with anti-IL8/APC (100x) and/or anti-Myc/DyLight 488 (500x, MA1-21316-D488, Thermo-Fisher Scientific, Rockford, IL) for 1 hr at room temperature. The cells were washed in Perm/wash buffer and analyzed by FACS Canto Flow Cytometer (BD Biosciences, San Jose, CA) and data processed using FlowJo software (TreeStar, Ashland, OR).

Immunoprecipitation

Immunoprecipitation of FLAG-CYLD, HOIP-V5, Myc-SPATA2 was from U2OS/NOD2 cells. Cells were transfected and treated as indicated. Cells were lysed in IP buffer (TBS with 0.5% NP40) supplemented with 5 mM NEM, 1 mM DTT, cOmplete protease inhibitor cocktail (Roche) and PhosSTOP (Roche) for 30 min on ice. Lysates were cleared by centrifugation and incubated at 4°C overnight with anti-FLAG affinity gel, or anti-V5-coupled beads. Beads were washed five times in 500 μL of ice-cold IP buffer and bound material eluted with 2x LDS. DTT was added to 1 mM and heated for 10 mins at 70 °C. For immunoprecipitation of endogenous CYLD and SPATA2 proteins from U2OS/NOD2 cells, confluent 10 cm plates were lysed in IP lysis buffer or TUBE lysis buffer supplemented with 5 mM NEM, cOmplete protease inhibitor cocktail and PhosSTOP and incubated 1-2 h with anti-CYLD (D1A10, Cell Signaling), anti-SPATA2 (A302-494A, Bethyl), or control rabbit IgG and then overnight with Protein A/G Sepharose. The immunoprecipitated material was washed with lysis buffer and eluted from the beads with Laemmli sample buffer. For Immunoprecipitation of endogenous SHARPIN, cells were lysed in buffer containing 25 mM HEPES (pH 7.4), 150 mM KCl, 2 mM $MgCl_2$, 1 mM EGTA, 0.5% Triton X-100, 5 mM NEM, 1 mM DTT, cOmplete protease inhibitor cocktail and PhosSTOP for 30 min on ice. After sedimentation of the cell debris, 5% of the supernatant was taken as input control. The remaining supernatant was incubated with anti-SHARPIN antibody (rabbit IgG served as negative control) and Protein A/G plus agarose for 4-6 hours at 4°C. After washing in lysis buffer, proteins were eluted from the resin by heating in 1.5x Laemmli sample buffer. For immunoprecipitation of the TNFR1 complex, 2 x 15cm plates of U2OS/NOD2 cells were treated with FLAG-TNF (Enzo Life Sciences, 100 ng/ml) for given time points and cells lysed in TNF lysis buffer (30 mM Tris HCl (pH 7.4), 120 mM NaCl, 2 mM EDTA, 2 mM KCl, 1% Triton X-100, cOmplete protease inhibitor cocktail and PhosSTOP) (Haas et al, 2009). Anti-FLAG affinity gel was added and incubated for a minimum of 2h at 4°C. Beads were washed five times in 500 μl of ice-cold IP buffer and bound material eluted with FLAG peptide (Sigma-Aldrich) at 100 μg/ml. Lysates were analyzed by immunoblotting.

Mass spectrometry analysis of CYLD interactome

Proteomic Sample Preparation. CYLD knock-out U2OS/NOD2 cells were transfected with FLAG-CYLD WT, FLAG-CYLD ΔBBOX, or control empty vector using Fugene 6. After 24 h cells were lysed in 0.5% NP-40/TBS lysis buffer and CYLD was immunoprecipitated using anti-FLAG Sepharose for 4 h at 4C. The immunoprecipitated material was eluted using FLAG peptide (Sigma Aldric) and was further prepared as previously described (Fischer et al., 2012). In brief, protein samples were reduced and alkylated in solution and subjected to chloroform/methanol precipitation. The precipitate was resuspended in 6 M urea in 100 mM Tris at a pH of 7.4, digested with elastase at 37 °C overnight, and desalted using C18 Sep Pak column cartridges (Waters, Elstree, UK).

Sample analysis. After drying down in vacuum, samples were resuspended in buffer A (98% H_2O, 2% acetonitrile, 0.1% formic acid) and analyzed by nano–liquid chromatography tandem mass spectrometry (nano–LC-MS/MS) in technical duplicates as described previously (Fischer and Kessler, 2015). In brief, samples were separated using a nanoUPLC (Easy spray C18 column with a 75 μm × 500 mm, 2.1 μm particle size; Thermo Fisher Scientific, Bremen, Germany) coupled to a Q Exactive tandem mass spectrometer (Thermo Fisher Scientific). MS data was acquired with a resolution of 70,000 at m/z 200 and selecting the Top 15 precursor ions. Ion target in MS1 was $3x10^6$ and $5x10^5$ in MS2 mode. Ions were accumulated for up to 100 ms in MS1 and 128 ms in MS2.

Proteomic Data Analysis. For label free quantitation, raw data was imported into Progenesis QI (Waters) Data was aligned and features detected using default settings. MS2 spectra of the combined data were converted to

.mgf files using the 200 most abundant peaks per MS2 spectrum, followed by identification with Mascot v2.5 (10 ppm precursor and 0.05 Da fragment mass tolerance, UniProt Swiss-Prot human database (retrieved 08/12/2013), 1% FDR, peptide score threshold of 20). Carbamidomethylation (C) was selected as fixed and deamidation (N, Q) and Oxidation (M) were selected as variable modifications. Quantitative data was re-normalized to all significantly identified peptides meeting the above mentioned selection criteria. For each replicate, the enrichment of proteins was calculated relative to the abundance in the IP control. The average of enrichment from each replicate is plotted in the figure using the ggplot2 package (www.ggplot2.org) and the R software environment (www.R-project.org).

RNA isolation, cDNA synthesis and qRT-PCR
Total RNA was isolated using an RNeasy Mini Kit (Qiagen) and DNase digestion was performed on-column with the RNase-Free DNase Set (Qiagen) according to the manufacturer's protocol. Total RNA was reverse transcribed with RevertAid™ Reverse Transcriptase (Thermo-Fisher Scientific) and mixture of anchored oligo(dT)$_{20}$ primers and random pentadecamers in the presence of RiboLock (Thermo-Fisher Scientific). qPCR was performed using SYBR Select Master Mix (Applied Biosystems). cDNA was amplified with the following primer pairs:
Hypoxanthine phosphoribosyltransferase (*HPRT*; used as reference for normalization):
5'-AGCCAGACTTTGTTGGATTTG-3' and 5'-TTTACTGGCGATGTCAATAGG-3',
SPATA2: 5'- TCGCTCAGCTCCTCTAGC-3' and 5'- AGGGCCCGTGTAGGTCTT-3', *TNF*: 5' - TGCTGCAGGACTTGAGAAGA-3' and 5' -GAGGAAGGCCTAAGGTCCAC-3',
CXCL8: 5'-TCTGGCAACCCTAGTCTGCT-3' and 5' -AAACCAAGGCACAGTGGAAC -3, *A20*: 5' - ATGCACCGATACACACTGGA-3' and 5' -GGATGATCTCCCGAAACTGA-3', *NFKBIA*: 5' - GCTGATGTCAATGCTCAGGA-3' and 5' -CCCCACACTTCAACAGGAGT-3'.

Transient RNAi knock-down
U2OS/NOD2 cells were reverse transfected with siRNA oligonucleotides (final concentration 35 nM siRNA oligo) using Lipofectamine RNAiMAX (Invitrogen Life Technologies) according to the manufacturer's instructions. For checking protein levels following *SPATA2* siRNA knockdown (**Figure S1C**), an additional forward transfection of the same siRNA was performed 12 hr following reverse transfection (indicated time points are from reverse transfection). The following siRNA oligonucleotides (Sigma-Aldrich) were used for RNAi-mediated knockdown:
CYLD: SASI_Hs01_00012965, GAAGAAUAUGUUUAGAUAU[dT][dT]
HOIP: GGCGUGGUGUCAAGUUUAA[dT][dT]
SPATA2(SP2-A): SASI_Hs01_00108531, CACCUUCACUCCAAAUCCA[dT][dT]
SPATA2(SP2-B): SASI_Hs01_00108532, CGAGUGUGAGCAGAUGCUA[dT][dT]
SPATA2(SP2-C): SASI_Hs01_00108533, GUGACCAAGCCCUCGAGGU[dT][dT]
Mismatched control (siMM): Mission siNEG Ctrl 1 (Cat. #SIC001)

Luciferase reporter assays
WT, CYLD KO and SPATA2 KO U2OS/NOD2 cells were co-transfected with the NF-κB luciferase reporter construct pBIIX-luc and a thymidine kinase-renilla luciferase construct for normalization of transfection efficiency. Cells were co-transfected with additional plasmids as indicated and assays were performed as previously described (Damgaard et al., 2012). Individual experiments were performed in duplicate.

Sucrose gradient sedimentation
Whole-cell lysate was prepared in IP buffer. Continuous 10-40% (w/v) sucrose gradients in a physiological salt solution (150 mM NaCl, 50 mM Tris pH 7.4, 5 mM EDTA, 1 mM PMSF) were generated using a Gradient Master™ 108 (Biocomp, Fredericton, Canada) and 500 µg of protein lysate was subjected to velocity sedimentation at 36,000 rpm (Optima™ L-100 XP (SW41 rotor), Beckman-Coulter), 4 degrees, for 16 hours. 13 fractions were collected and protein was precipitated with trichloracetic acid at a concentration of 8.4%, incubated on ice for 60 min, and centrifuged at 14,000g for 5 min. The resulting pellet was washed 2x with acetone and resuspended in 2x Laemmli buffer.
To approximate the hydrodynamic radii of individual protein complexes, a mixture of purified, native gel filtration standards (Gel Filtration Markers Kit, MWGF1000, Sigma) was subjected to sucrose gradient sedimentation. Fractions were collected and processed as described above. The sedimentation pattern of each protein standard was determined by SDS-PAGE and subsequent silver staining (Silver Stain for Mass Spectrometry, Pierce).
For densitometry analysis, the intensity of each fraction, minus background signal, was quantified in ImageJ. The resulting values were normalised and plotted relative to the highest intensity value in each blot. Data shown representative of three biological replicates. Data represents mean ± SEM.

Generation of knockout cells by CRISPR/Cas9

To generate knockout U2OS/NOD2 cells, cells were transfected using Fugene HD with CRISPR/Cas9 KO Plasmid (Santa Cruz Biotechnology) containing gRNA, Cas9 and EGFP marker. For generation of SPATA2 KO, sc-406210 was used; for CYLD KO, sc-400882 was used.

After 36 h top 10 % GFP-positive cells were sorted by Flow Cytometry and cloned by limiting dilution to obtain single cell clones. Individual clones were validated by western blotting with SPATA2 or CYLD specific antibodies and, for SPATA2 KO clones, co-immunoprecipitation with endogenous CYLD (**Figure 1H; Figure S1E**). The generation of HCT116 HOIP KO cells was previously described (Hrdinka et al., 2016).

Cell transfection

U2OS/NOD2 cells were transiently transfected using Fugene 6 transfection reagent (Promega, Madison, WI) according to manufacturer's instructions.

Statistical analysis

Statistical analysis was performed using Prism 5 or 6 (GraphPad Software Inc). Two-way ANOVA was used to determine statistical significance, except in **Figure 1C** where the two-tailed Student's test was used.

Protein expression and purification

His6-GST-SPATA2 PUB domain constructs were expressed in Rosetta2 (DE3) pLacI cells. Cells were grown at 30 °C in 2xTY medium supplemented with 30 μg/ml kanamycin and 34 μg/ml chloramphenicol to an OD600 of 0.6-1.0. The culture was cooled to 16 °C prior to overnight induction with 400 μM IPTG. Cells were resuspended and lysed by sonication in lysis buffer (20 mM Tris pH 7.4, 300 mM NaCl, 50 mM imidazole, 2 mM β-mercaptoethanol, 10 % (v/v) glycerol, lysozyme, DNaseI (Sigma), 1 mM PMSF and protease inhibitor cocktail (Roche). SPATA2 was purified by immobilised metal affinity chromatography using a HisTrap column (GE Life Sciences). Pooled fractions were dialysed overnight into cation exchange chromatography buffer (20 mM MES pH 6.0, 50 mM NaCl, 4 mM DTT and 10% (v/v) glycerol). For biophysical and structural analysis the His6-GST-tag was cleaved by overnight incubation with 3C protease during dialysis. GST-SPATA2 and SPATA2 PUB domain constructs were purified by cation exchange chromatography (sulphopropyl (SP), GE Life Sciences). Eluted fractions were subjected to size exclusion chromatography (HiLoad 16/60 Superdex 75, GE Life Sciences) in buffer containing 20 mM Tris pH 7.4, 600 mM NaCl, 4 mM DTT. The resultant fractions were judged to be 95-99% pure following SDS-PAGE analysis and flash frozen. HOIP PUB domain was expressed and purified according to (Elliott et al., 2014). Owing to proteolysis of SPATA2 C-terminal of the PUB domain within *E. coli*, the SPATA2 PUB-PIM sequence (7-356) was coexpressed with the HOIP PUB domain that had been sub-cloned into the pETDuet vector (Novagen). GST-SPATA2 (7-356) and His6-HOIP (1-184) were purified using HisTrap affinity and then anion exchange chromatography in buffer containing 20 mM Tris pH 8.5, 10 % (v/v/) glycerol and 4 mM DTT, which enable the removal of excess HOIP before finally being purified by size exclusion chromatography (HiLoad 16/60 Superdex 75, GE Life Sciences). CYLD USP was expressed in sf9 cells and purified according to (Komander et al., 2008) with the exception that 10 %(v/v) glycerol was included in the anion exchange buffer (20 mM Tris pH 8.5, 4 mM DTT, 10 % (v/v) glycerol).

GST pull-down assays

30 μg GST, GST-HOIP PUB (1-184) and GST-SPATA2 PUB (1-241) variants were bound to 25 μl glutathione resin (packed volume) (GE Life Sciences) and incubated for 1 hr at 4 °C in 450 μl buffer (20 mM Tris pH 7.4, 600 mM NaCl, 4 mM DTT). Resin were washed in 3 x 450 μl buffer and incubated with 30 μg CYLD USP variants (in slight molar excess) in 200 μl buffer for 1 hr at 4 °C. Resin were washed in 4 x 450 μl to remove non-bound protein before the resin were resuspended in 50 μl SDS loading buffer prior to SDS-PAGE analysis.

Analytical size exclusion chromatography binding studies

Binding studies with purified HOIP, SPATA2 and CYLD variants were performed on an AKTA Micro system (GS Life Sciences) using a Superdex 75 PC 3.2/30 column equilibrated in: 20 mM Tris pH 7.4, 400 mM NaCl, 2 mM DTT. For studies involving HOIP and SPATA2 PUB domains, 50 μM of each PUB domain was mixed with 50 μM CYLD at room temperature for 10 minutes. Fractions containing protein were mixed with SDS loading buffer prior to SDS-PAGE analysis.

Multi-angle light scattering

The mass of SPATA2 and CYLD complexes in solution was determined by size exclusion multi-angle light scattering (SEC-MALS) using either a Superdex 75 10/300 or Superdex 200 10/300 (GE Life Sciences) connected to a Wyatt Heleos II 18 angle light scattering instrument coupled to a Wyatt Optilab rEX online refractive index detector. Protein samples (100 μl of 2 mg/ml, with the exception of the CYLD B-box 3 mg/ml

and SPATA2 CYLD complex (50 μM) 3.3 mg/ml) were loaded at 0.5 ml/min in buffer containing 20 mM Tris pH 7.4, 200 mM NaCl, 2 mM DTT. A BSA run at 2 mg/ml was used to determine inter-detector delay and peak broadening effects. The protein concentration was determined from the excess differential refractive index based on 0.186 RI per 1 g/ml. The concentration and observed scattering intensities were used to calculate the absolute molecular mass using the ASTRA6 software (Wyatt technology). Partial dissociation of each complex results in a lower observed molecular weight, and the complex fraction can be calculated by dividing the observed molecular weight by the expected/calculated molecular weight. An additional 10% error can be assumed from SEC-MALS measurements.
We estimate the stoichiometry of the trimeric HOIP–SPATA2–CYLD complex as 2:2:2 due to the lack of unbound protein in the SEC MALS run that was performed with equimolar amounts of protein. The lower observed molecular weight (170 kDa as compared to 208 kDa) likely originates from partial complex dissociation, which is already observed in individual binary complexes (see complex fraction), and is likely more pronounced in the trimeric complex.

Crystallization, data collection and refinement

Conventional methods to obtain crystals of the SPATA2 PUB domain failed to yield any crystals, during screening of different SPATA2 PUB domain constructs and crystallisation conditions. Serendipitously, crystals of the SPATA2 PUB domain were obtained from a construct (7-219) that had been purified by size exclusion chromatography (HiLoad 16/60 Superdex 75, GE Life Sciences) in buffer containing 20 mM MES pH 6.0, 200 mM NaCl, 4 mM DTT. The resultant fractions could not be concentrated beyond ~2 mg/ml however, after incubation at 4 °C over a period of a week crystals were observed in the concentrator. Crystals were transferred to a solution containing 20 mM MES pH 6.0, 200 mM NaCl, 4 mM DTT and 30 % (v/v) glycerol prior to cryo-cooling. Crystals of the HOIP PUB domain (5-180) in complex with SPATA2 PIM (334-344) were grown by sitting-drop vapour with 1.5 molar excess of SPATA2 PIM peptide. HOIP SPATA2 PIM was mixed with reservoir containing 1.7-1.9 M $(NH_4)_2SO_4$, 50 mM sodium cacodylate pH 6.4-7.0, 15 mM MgCl2 in a 1:2 ratio. Crystals were transferred into a solution containing 2M lithium sulphate prior to cryo-cooling.

Diffraction data were collected at Diamond Light source beamline I02. Diffraction images were processed using xia2 (Winter, 2010) and manually scaled using AIMLESS (Evans and Murshudov, 2013). Owing to the low sequence homology between SPATA2 and the HOIP/PNGase PUB domains (25% sequence identity) and slight deviation of core helices relative to one another, conventional molecular replacement was not sufficient for determining the SPATA2 PUB structure. The *ab initio* phase determining programme AMPLE was used to determine the structure of the SPATA2 PUB domain through placement of short poly-Ala idealised helices (Bibby et al., 2012). Manual inspection of the search models and additional molecular replacement of idealised helices using PHASER (McCoy et al., 2007) resulted in suitable phases that allowed automatic building of the resultant electron density map using ARP/wARP (Langer et al., 2008). The structure of the HOIP-SPATA2 PIM peptide complex was determined by molecular replacement using PHASER (McCoy et al., 2007) and the HOIP PUB domain (PDB ID 4OYK) with the OTULIN PIM peptide deleted. Iterative rounds of model building and refinement were performed with COOT (Emsley et al., 2010) and PHENIX (Adams et al., 2011) respectively. In the case of the HOIP PUB SPATA2 PIM complex, unambiguous electron density could be built into for the SPATA2 PIM peptide after initial rounds of refinement. Data collection and refinement statistics can be found in **Table 1**. All structure figures were generated with Pymol (www.pymol.org).

DUB assays

Qualitative gel-based DUB assays were performed as in (Komander et al., 2009). Briefly, CYLD USP was diluted in 25 mM Tris pH 7.4, 200 mM NaCl and 5 mM DTT and activated at 21 °C in the absence or presence of SPATA2 PUB for 15 min. 10 μM tetraUb or 4 μM diUb was incubated with indicated amounts of CYLD in 50 mM Tris pH 7.4, 100 mM NaCl, 4 mM DTT at 25 °C. Time points were taken and mixed with SDS sample buffer to stop the reaction. Samples were resolved on 4-12% SDS-PAGE gradient gels and visualised by silver staining (Biorad).

Fluorescence polarisation binding assays

Serial dilutions of either HOIP/SPATA2 PUB domain variants into FP assay buffer: 20 mM Tris pH 7.4, 300 mM NaCl, 2 mM DTT were prepared and 10 μl of this was aliqutoed into a 384-well low volume plate (Corning) containing 10 μl of 100 nM FITC-Ahx PIM peptides of either OTULIN (49-67), p97 (797-806) or SPATA2 (334-344). Fluorescence polarisation was recorded on a PheraStar plate reader (BMG Labtech) using an optics module with λex = 485 nm and λem = 520 nm. Polarisation values were plotted against PUB concentration and fitted to a one-site binding model using Graphpad Prism 5.

Fluorescence polarisation cleavage assays

Change in fluorescence polarisation upon cleavage of the peptide/isopeptide bond between the distal and proximal ubiquitin moieties of Met1/Lys63 diUb were used to derive Michaelis-Menten rates. Reactions were performed in a black 384-well low volume plate (Corning) and measured on a PheraStar plate reader (BMG Labtech) using an optics module with λex = 485 nm and λem = 520 nm. Assays were performed based upon (Keusekotten et al., 2013). Briefly, Met1/Lys63 diUb were serially diluted into FlAsH buffer (20 mM Tris pH 7.4, 200 mM NaCl, 2 mM β-mercaptoethanol, 0.04 mg/ml BSA) and contained a fixed concentration of 300 nM FlAsH-tagged Met1/Lys63 diUb. To each well, 10 µl of substrate was mixed with 10 µl of CYLD USP (583-956) and the change in fluorescence polarisation was recorded at 25 °C. Observed fluorescence polarisation values were converted to percentage of substrate cleavage by comparing to baseline values of FlAsH-tagged monoUb. All measurements were corrected by subtracting changes in fluorescence polarisation of FlAsH-tagged Met1/Lys63 diUb alone and all measurements were performed in triplicate. Initial rates of substrate cleavage were calculated using GraphPad Prism 5. Initial rates were plotted against substrate concentration at a fixed CYLD concentration. The K_M of CYLD USP for Met1- and Lys63-diUb is greater than the highest substrate concentrations used in the assay, preventing direct determination of Michaelis-Menten parameters. Therefore, the DUB activity (k_{cat}/K_M) was calculated from a linear fit of substrate concentration plotted against initial rate and corrected against amount of CYLD USP used in the assay. To enable efficient initial rates, 3 µM CYLD USP +/- 10 µM SPATA2 PUB was used for Lys63 diUb cleavage and 0.4 µM CYLD USP +/- 4 µM SPATA2 PUB was used for Met1 diUb cleavage.

Nuclear Magnetic Resonance Spectroscopy
His6-SUMO-tagged CYLD B-box (778-855) was expressed in 2M9 medium supplemented with ^{15}N NH_4Cl and purified using standard HisTrap and anion exchange protocols described above. BEST-TROSY spectra (Solyom et al., 2013) were acquired at 298K on Bruker Avance2+ 700 MHz spectrometer equipped with a cryogenic triple TCl probe. Data processing and analysis were performed with Topspin3.0 (Bruker) and Sparky (http://www.cgl.ucsf.edu/home/sparky/).

Analytical Ultracentrifugation
Equilibrium sedimentation experiments for a complex of SPATA2 PUB and CYLD USP were performed on an Optima XL-I analytical ultracentrifuge (Beckmann) using An50Ti rotors. Sample volumes of 110 µl with protein concentrations of 1 µM in 20 mM HEPES, pH 7.5, 200 mM NaCl, 4 mM DTT were loaded in 12 mm 6-sector cells and centrifuged at 5700, 6900, and 12000 rpm until equilibrium was reached at 20 ˚C. At each speed, comparison of several scans was used to judge whether or not equilibrium had been reached. Data were processed and analysed using UltraSpin software (http://www.mrc-lmb.cam.ac.uk/dbv/ultraspin2/) and SEDPHAT (Schuck, 2003). The partial-specific volumes (v-bar), solvent density and viscosity were calculated using SEDNTERP (Dr. Thomas Laue, University of New Hampshire.

Surface Plasmon Resonance
SPR was performed using a Biacore T200 using CM5-sensor chips (GE Healthcare). Both reference control and analyte channels were equilibrated 20 mM HEPES, pH 7.5, 200 mM NaCl, 4 mM DTT. SPATA2 PUB was immobilised onto the chip surface *via* amide coupling using the supplied kit (GE Healthcare) to reach an RU value of between 100 and 300 for separate experiments. SPR runs were performed with analytes injected for 120 s followed by a 600 s dissociation in a 1:2 dilution series with initial concentrations of: 2 µM for CYLD USP WT and I790D; 55.2 µM for CYLD L622D; 63 µM for CYLD ΔB-Box; and 484 µM for CYLD B-box (778-855). After reference and buffer signal correction, sensorgram data were fitted using KaleidaGraph (Synergy Software) and Prism (Graphpad Prism).
The kinetic rate constants of dissociation were measured by fitting dissociation data at time t (R_{dissoc}) using a single-exponential function:

$$R_{dissoc} = R_o \exp^{-(k_{off}t)} + RI + Dt \quad (1)$$

where k_{off} is the dissociation rate constant, R_{o} is maximum change in resonance, RI is the bulk resonance change and D is a linear drift term. The rate constants of association were obtained by fitting the observed change in resonance signal (R_{assoc}) at time t using the following equation:

$$R_{assoc} = \left(\frac{k_{on}CR_{\max}}{k_{on}C + k_{off}}\right)\left[1 - \exp^{-(k_{on}C + k_{off})t}\right] + RI + Dt \quad (2)$$

where k_{on} is the association rate constant, C is the analyte concentration and $R_{\max}$ is the maximum change in resonance. The affinity for the interactions were calculated from the ratios of the microscopic rate constants:

$$K_d = \frac{k_{off}}{k_{on}} \tag{3}$$

For CYLD USP WT and I709D, the equilibrium response (R_{eq}) data were fitted using a single site interaction model to determine K_d:

$$R_{eq} = \left(\frac{CR_{max}}{C + K_d}\right) + N_s C + B \tag{4}$$

where C is the analyte concentration and R_{max} is the maximum response at saturation, N_s is a linear non-specific binding term and B is the background resonance. Data were transformed to fraction bound with the fitted values using:

$$f_b = \left(\frac{R - B}{R_{max} + N_s C}\right) \tag{5}$$

For L622D and ΔBox, as saturation was not reached, data were transformed using parameters fitted for WT.

Supplemental references

Adams, P.D., Afonine, P.V., Bunkoczi, G., Chen, V.B., Echols, N., Headd, J.J., Hung, L.W., Jain, S., Kapral, G.J., Grosse Kunstleve, R.W., *et al.* (2011). The Phenix software for automated determination of macromolecular structures. Methods *55*, 94-106.

Berrow, N.S., Alderton, D., Sainsbury, S., Nettleship, J., Assenberg, R., Rahman, N., Stuart, D.I., and Owens, R.J. (2007). A versatile ligation-independent cloning method suitable for high-throughput expression screening applications. Nucleic Acids Res *35*, e45.

Bibby, J., Keegan, R.M., Mayans, O., Winn, M.D., and Rigden, D.J. (2012). AMPLE: a cluster-and-truncate approach to solve the crystal structures of small proteins using rapidly computed ab initio models. Acta Crystallogr D Biol Crystallogr *68*, 1622-1631.

Elliott, P.R., Nielsen, S.V., Marco-Casanova, P., Fiil, B.K., Keusekotten, K., Mailand, N., Freund, S.M., Gyrd-Hansen, M., and Komander, D. (2014). Molecular Basis and Regulation of OTULIN-LUBAC Interaction. Mol Cell *54*, 335-348.

Emsley, P., Lohkamp, B., Scott, W.G., and Cowtan, K. (2010). Features and development of Coot. Acta Crystallogr D Biol Crystallogr *66*, 486-501.

Evans, P.R., and Murshudov, G.N. (2013). How good are my data and what is the resolution? Acta Crystallogr D Biol Crystallogr *69*, 1204-1214.

Fiil, B.K., Damgaard, R.B., Wagner, S.A., Keusekotten, K., Fritsch, M., Bekker-Jensen, S., Mailand, N., Choudhary, C., Komander, D., and Gyrd-Hansen, M. (2013). OTULIN restricts Met1-linked ubiquitination to control innate immune signaling. Mol Cell *50*, 818-830.

Fischer, R., and Kessler, B.M. (2015). Gel-aided sample preparation (GASP)--a simplified method for gel-assisted proteomic sample generation from protein extracts and intact cells. Proteomics *15*, 1224-1229.

Fischer, R., Trudgian, D.C., Wright, C., Thomas, G., Bradbury, L.A., Brown, M.A., Bowness, P., and Kessler, B.M. (2012). Discovery of candidate serum proteomic and metabolomic biomarkers in ankylosing spondylitis. Mol Cell Proteomics *11*, M111 013904.

Haas, T.L., Emmerich, C.H., Gerlach, B., Schmukle, A.C., Cordier, S.M., Rieser, E., Feltham, R., Vince, J., Warnken, U., Wenger, T., *et al.* (2009). Recruitment of the linear ubiquitin chain assembly complex stabilizes the TNF-R1 signaling complex and is required for TNF-mediated gene induction. Mol Cell *36*, 831-844.

Hrdinka, M., Fiil, B.K., Zucca, M., Leske, D., Bagola, K., Yabal, M., Elliott, P.R., Damgaard, R.B., Komander, D., Jost, P.J., *et al.* (2016). CYLD Limits Lys63- and Met1-Linked Ubiquitin at Receptor Complexes to Regulate Innate Immune Signaling. Cell Rep *14*, 2846-2858.

Keusekotten, K., Elliott, P.R., Glockner, L., Fiil, B.K., Damgaard, R.B., Kulathu, Y., Wauer, T., Hospenthal, M.K., Gyrd-Hansen, M., Krappmann, D., *et al.* (2013). OTULIN antagonizes LUBAC signaling by specifically hydrolyzing Met1-linked polyubiquitin. Cell *153*, 1312-1326.

Komander, D., Lord, C.J., Scheel, H., Swift, S., Hofmann, K., Ashworth, A., and Barford, D. (2008). The structure of the CYLD USP domain explains its specificity for Lys63-linked polyubiquitin and reveals a B box module. Mol Cell *29*, 451-464.

Komander, D., Reyes-Turcu, F., Licchesi, J.D., Odenwaelder, P., Wilkinson, K.D., and Barford, D. (2009). Molecular discrimination of structurally equivalent Lys 63-linked and linear polyubiquitin chains. EMBO Rep *10*, 466-473.

Langer, G., Cohen, S.X., Lamzin, V.S., and Perrakis, A. (2008). Automated macromolecular model building for X-ray crystallography using ARP/wARP version 7. Nature protocols *3*, 1171-1179.

McCoy, A.J., Grosse-Kunstleve, R.W., Adams, P.D., Winn, M.D., Storoni, L.C., and Read, R.J. (2007). Phaser crystallographic software. J Appl Crystallogr *40*, 658-674.

Solyom, Z., Schwarten, M., Geist, L., Konrat, R., Willbold, D., and Brutscher, B. (2013). BEST-TROSY experiments for time-efficient sequential resonance assignment of large disordered proteins. J Biomol NMR *55*, 311-321.

Winter, G. (2010). xia2: an expert system for macromolecular crystallography data reduction. Journal of Applied Crystallography *43*, 186-190.
